# Supplementary material for: Open-source automated chemical vapor deposition system for the production of two- dimensional nanomaterials
Source: PLoS One. 2019 Jan 16;14(1):e0210817. doi: 10.1371/journal.pone.0210817 (PMC6334948; doi:10.1371/journal.pone.0210817)
Supplement: S2 Appendix — (PDF) [file pone.0210817.s002.pdf]

Notes on CVD Construction

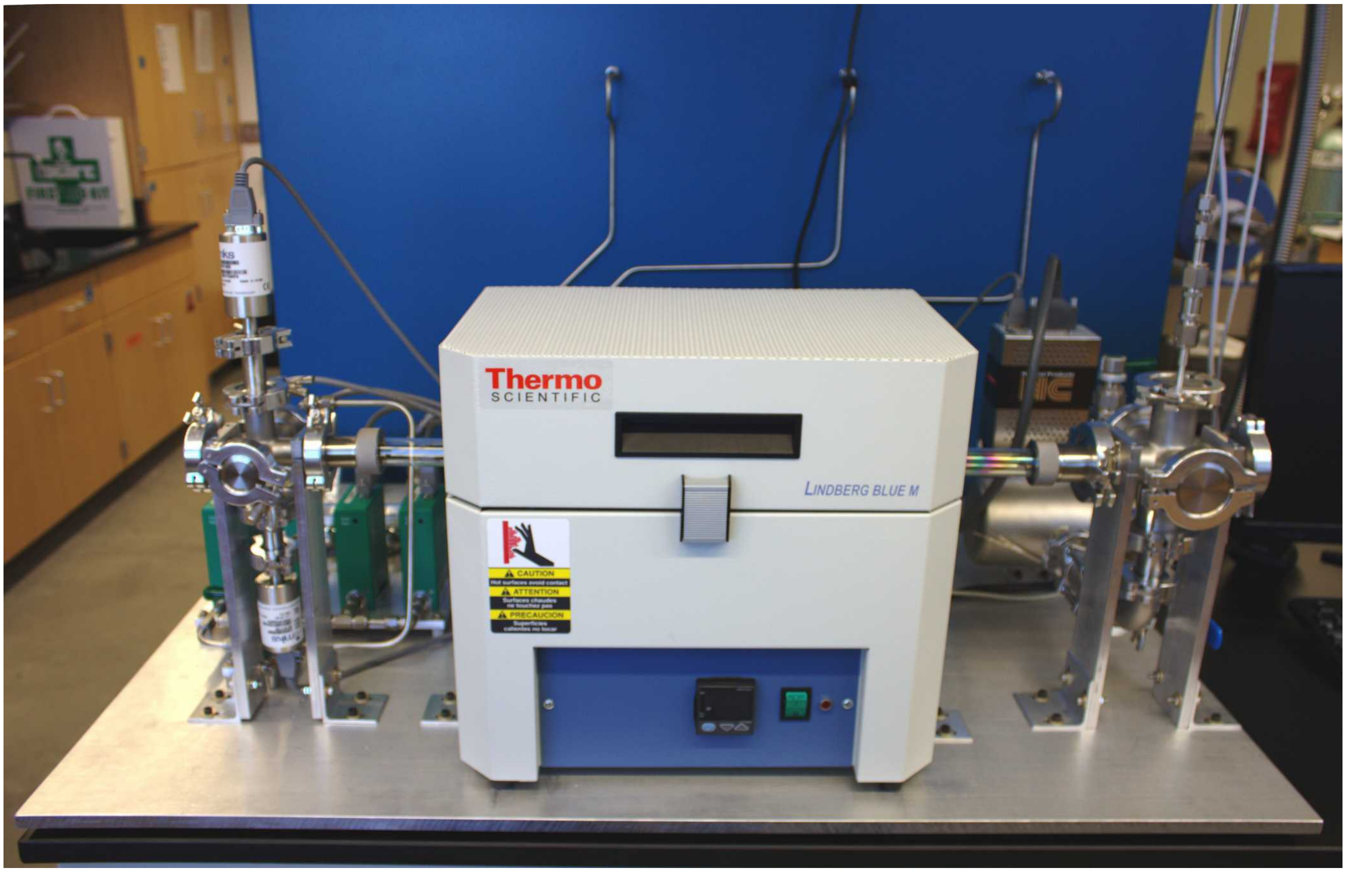

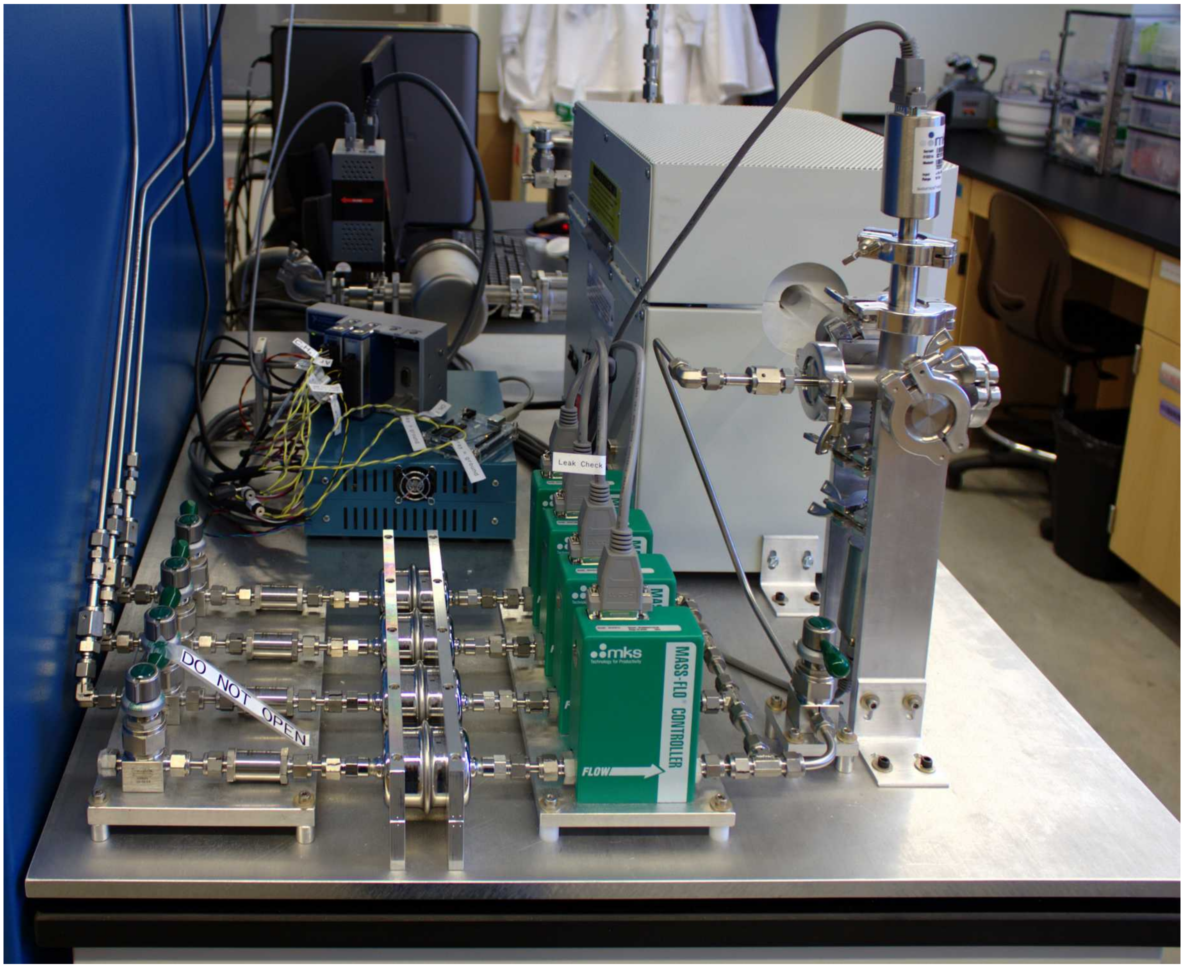

General comments

- This document was written to be a general guide and is a technical reference that can replace OEM manuals and instructions.
- Part IDs shown in red. See each subsystem's tab in the parts list spreadsheet for the supplier and part number of each part ID.
- See Swagelok instructions for ¼” Swagelok tube adapters. In general, tubes were cut to required length, deburred, rinsed with IPA (inside and outside part to be inserted into tube adapter), fit parts together without tightening to verify spacing, then tube adapters were tightened 1 and ¼ turns beyond hand tightening. Tube adapter connections should not be broken once made.
- ¼” tubing is hard and it can be difficult to get a clean cut when using a handheld tube cutter. To remedy this we placed one side of the ¼” tube to be cut in the chuck of a cordless drill, tightened a pipe cutter onto the tube, cycled the cutter once around to make the initial groove, then ran the drill while slowly tightening the pipe cutter until the tube was severed. Tube was held in place with cardboard or some other stiff material that would not wrap around the tube as it turned.
- Do not over-tighten KF cast clamps. The wing nuts are made of a soft metal and their threads can be destroyed when too much torque is applied. A cast clamp only needs to be snug for a seal to form between the O-ring and the flanges it bridges.
- Place plastic covers on open KF flanges to avoid dust accumulation in CVD.
- Corners and edges of base and support aluminum pieces should be filed and sanded to get rid of sharp edges.
- Support structure drawings are provided in the “Drawings/” Folder of the supplementary material. Some details of the gas filter line valve supports were modified during fabrication. Also, see the exhaust manifold support drawing for details on the cap screw holes on the top of the support as reference for the intake manifold and exhaust line supports.

Order of assembly

1. Obtain baseplate, file/sand edges and corners, install feet, put in place.
2. Manufacture solid angle supports for furnace, drill mounting holes in side of furnace (this may void the furnace's warranty, but mounting is necessary to keep furnace from being bumped during growth and breaking the reaction tube), mount supports onto furnace (do not mount to base plate yet).
   - Be sure that furnace internals are not damaged during drilling. Controller face should be removed so that the inside of the furnace can be observed.
3. Place furnace on base plate, assemble reaction tube assembly and place into furnace (be careful with reaction tube assembly as ends of quartz tube can easily chip).
4. Determine height of intake and exhaust manifold supports (determined by height of reaction tube assembly in furnace), manufacture supports, mount manifolds into supports, attach intake and exhaust manifolds to reaction tube (with reaction tube in furnace).
5. Assemble exhaust line, mount to exhaust manifold to determine height of exhaust line supports, manufacture exhaust line supports, mount exhaust line (and butterfly valve, but do not place butterfly valve itself into supports) in supports and connect to exhaust manifold.
6. Manufacture supports for gas filter lines, MFCs, gas mixing line, mount gas filter line valves, MFCs, gas mixing line valve.
7. Cut ¼” tubing for gas filter lines and gas mixing line (except the long piece in gas mixing line – part 7), assemble gas filter lines and gas mixing line (except parts 7 through 12) with MFCs in place **without tightening ¼” tube adapter connections**. VCR connections to the UHP gas filters can be tightened during this step.
8. Finalize location of subsystems.
   - Verify that both ends of the quartz tube are inside of the quick disconnects so that quartz tube assembly can easily be removed when changing tubes.
   - Furnace should be able to slide forward on its supports (with the supports disconnected from the base plate) so that a quartz tube can be passed through it without hitting the intake or exhaust manifolds. This is so that new tubes can be baked along their entire length before they are installed.
   - Ensure that bellows can be connected to the end of the exhaust line.
   - Ensure that the elbows in the gas delivery lines (part 3) will fit between the diaphragm valves (part 5) and whatever is behind the CVD.
9. Drill, tap holes in base plate.
   - From gas filter lines to gas mixing line: Once subsystem position has been chosen, a marker is used to mark the base plate through the holes in the support plates. An automatic center punch is then used in the middle of each of the marks, alignment of the dimples with the support mounting holes is double checked, then holes are drilled

using a vertical drill guide and the resulting holes are tapped.
  - Mounting KF supports to base plate: Once the desired positions of the intake manifold, furnace, exhaust manifold and exhaust line are chosen, marks and center punch dimples are made on the base plate through the center of the solid angles slots in the base of the KF supports. The marked spots are then drilled and tapped.
  - With all holes drilled and tapped, verify that mounting bolts fit into their respective holes, then empty base plate and clean up aluminum waste on and under base plate.

10. Mount support structures.
    - Mount gas filter line support to the base plate (with valves mounted – verify that flow directions are correct), place MFC support plate onto base plate (with MFCs mounted – again, verify flow directions are correct), assemble filter lines between valves and MFCs **without tightening ¼” tube adapter fittings**, mount MFC base plate, assemble gas mixing line up to gas mixing line valve (**without tightening ¼” tube adapter fittings**), mount gas mixing line valve support to base plate.
    - Assemble intake manifold, reaction tube, furnace, exhaust manifold, and exhaust line and mount to the base plate.
11. Tighten ¼” tube adapter fittings from the valves in the gas filter lines (gas filter lines, part 5) to the valve in the gas mixing line (gas mixing line, part 6)
12. Assemble gas mixing line, parts 9-12, attach to intake manifold, place elbow (part 8) onto part 10 **without tightening tube adapter fitting.**
13. Cut, bend, and clean long tube in gas mixing line (gas mixing line, part 7), verify fit from valve (part 6) to elbow (part 8), tighten ¼” tube adapter fittings on elbow, valve.
14. Install pressure gauge assemblies onto top and bottom of intake manifold. Place the pressure gauge with the lowest full range on the top.
    - One of the intake manifold supports will likely have to be removed for cast clamps to be installed on pressure gauge assemblies.
15. Install vent valve.
    - Flow arrow should point toward the exhaust manifold.
16. Install snorkel manifold.
17. Install overpressure relief line.
    - Flow arrow on overpressure relief valve should point away from the exhaust manifold.
18. Install remaining blank flanges onto intake and exhaust manifolds.
19. Drill holes through back of gas cabinet for bulkhead reducers (gas delivery lines, part 8), install bulkhead reducers.
20. Cut, clean part 4 of the gas filter lines, tighten the ¼” tube adapter connections on the diaphragm valves only (**not on the elbows**).
21. Assemble parts 11-14 of the gas delivery lines with parts 1-3 of the gas filter lines, tighten ¼” tube adapter and VCR fittings (**only for these parts, do not attach the elbows (part 3) to part 4 of the gas filter lines**).

22. Cut, bend, attach ¼” gas delivery line tubes (gas delivery lines, part 10).
    - Tube fabrication order: first bend gooseneck, then 90º bend, then 45º bend, then dry fit tube into bulkhead reducer, line up tube with straight union (part 11) while assembly from the previous step is placed on but not attached to part 4 of the gas filter lines. When alignment is verified, mark and cut tube so that the lower end fits into the straight union.
    - Determining the location of bends in the tube can be difficult. Placing and marking sticky notes onto the surface behind them can help with planning.
    - Do not run tubes in a straight line from the bulkhead reducer to the straight union so that there will be some play in the locations of the ends of the gas delivery lines.
23. Assemble parts 1-8 of gas delivery lines.
    - Tighten the tube adapter fittings on the elbow (part 3) last.
24. Construct power supplies.
    - Consult your institution's engineering staff.
    - Could be contained in a single box to save on space.
    - Device power requirements given on the 'Power supplies' tab of the parts list spreadsheet.
25. Fabricate cables.
    - For device pinouts, see appendix A.
    - Cables should be plugged into respective devices before they are cut to ensure the correct connector is preserved.
    - Cable color/pinout tables in appendix A should be verified with a multimeter.
    - MFC: Only one leak check cable necessary as gas lines need only be checked for leaks once.
26. Assemble cDAQ for data acquisition and control.
27. Connect devices to power, cDAQ, control PC via cables.
28. Install drivers.
    - cDAQ
    - USB to RS485 adapter
29. Install vacuum pump.

(For steps 30 through 32, see Appendix B: Control Program Setup)

30. Verify functionality of LabView device driver subVIs according to the instructions in Appendix B. Device driver subVIs are located in the “Programs/sub-VIs/” folder.
    - Butterfly valve

- Furnace
- MFCs
- Pressure gauges

31. Setup Manual Controls VI according to the instructions in Appendix B. ManualControls.vi is located in the “Programs/” folder.
    - Set furnace temperature, set pressure, set flow rates, stop program, check log file.
32. Setup the Read & Run Recipe VI according to the instructions in Appendix B. Read&RunRecipe.vi is located in the “Programs/” folder.
33. Conduct leak check of system, gas delivery lines.
    - Most subsystems can be isolated via valves.
    - Gas delivery/filter lines can be checked by unplugging all MFC cables except the 'Leak Check' cable, setting the leak check cable to open, then plugging it into the MFC of the gas line to check. Valve on gas cylinder should be turned off during gas delivery line leak check.
    - System should easily get below 50 mTorr, but your results may vary.
34. **CVD is ready for use.**
35. For notes on recipe requirements, see Quick.csv in “Programs/Recipes/” folder in the supplementary material. Quick.csv can be run as-is via Read&RunRecipe.vi as an example.
36. After a CVD run is terminated, use the Excel macro in Appendix C to quickly graph the settings and recorded data from the run. Log files will be located in “Programs/CVD Run Logs/”. This folder will also contain a copy of the recipe used in the case of an automatic run. These recipes can be used as-is to control the CVD and will record the file paths of the original recipes in their notes.

Subsystem 1: Gas delivery lines

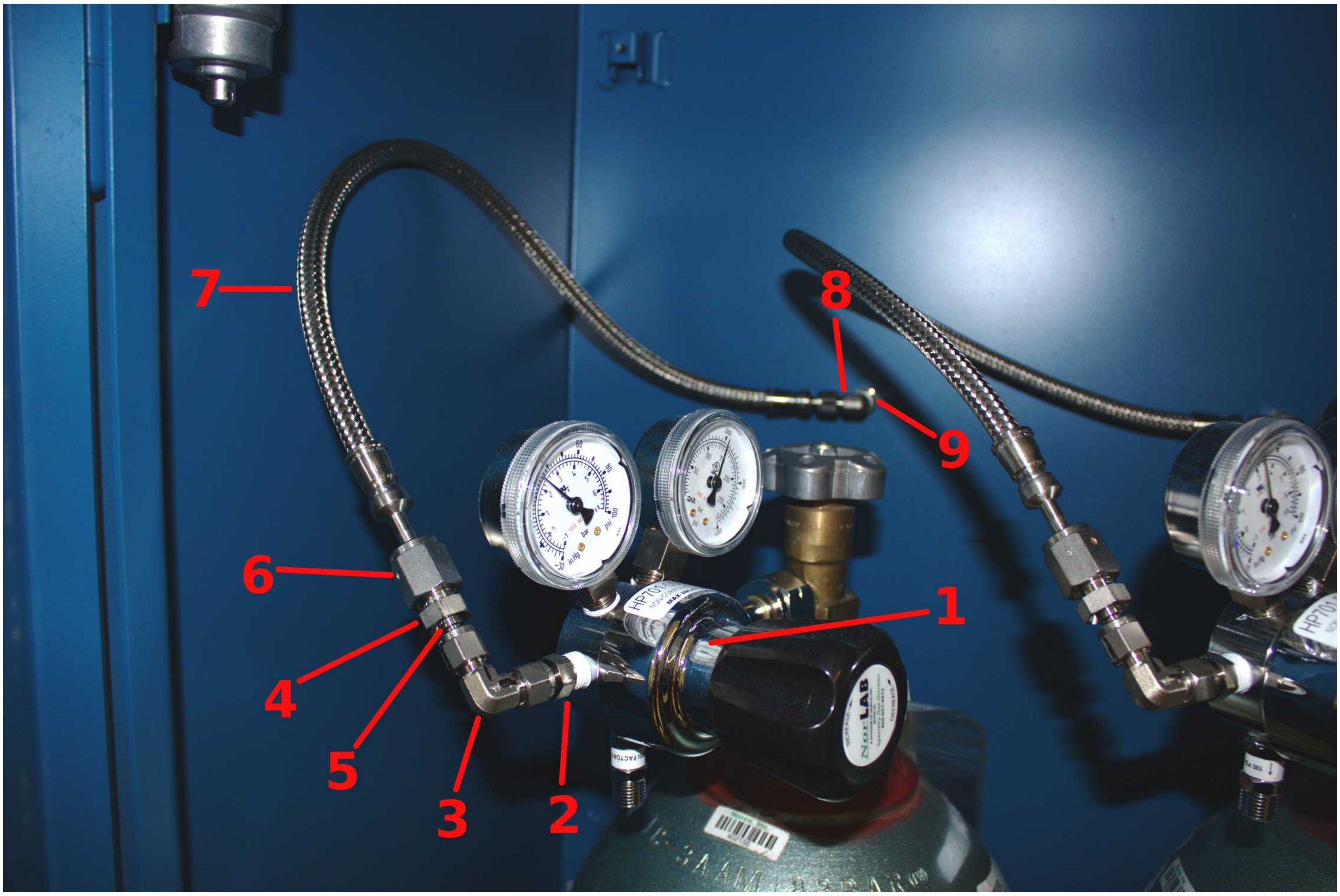

Comments:

- Teflon tape needed between parts 1 and 2.
- Leave tube adapter connections loose until alignment and spacing is verified.
- Hole must be drilled in gas cabinet wall for bulkhead reducer (part 9).
- To change gas, VCR connection is disconnected and then regulator is detached from cylinder. When regulator is attached to a new cylinder, VCR gasket must be replaced.

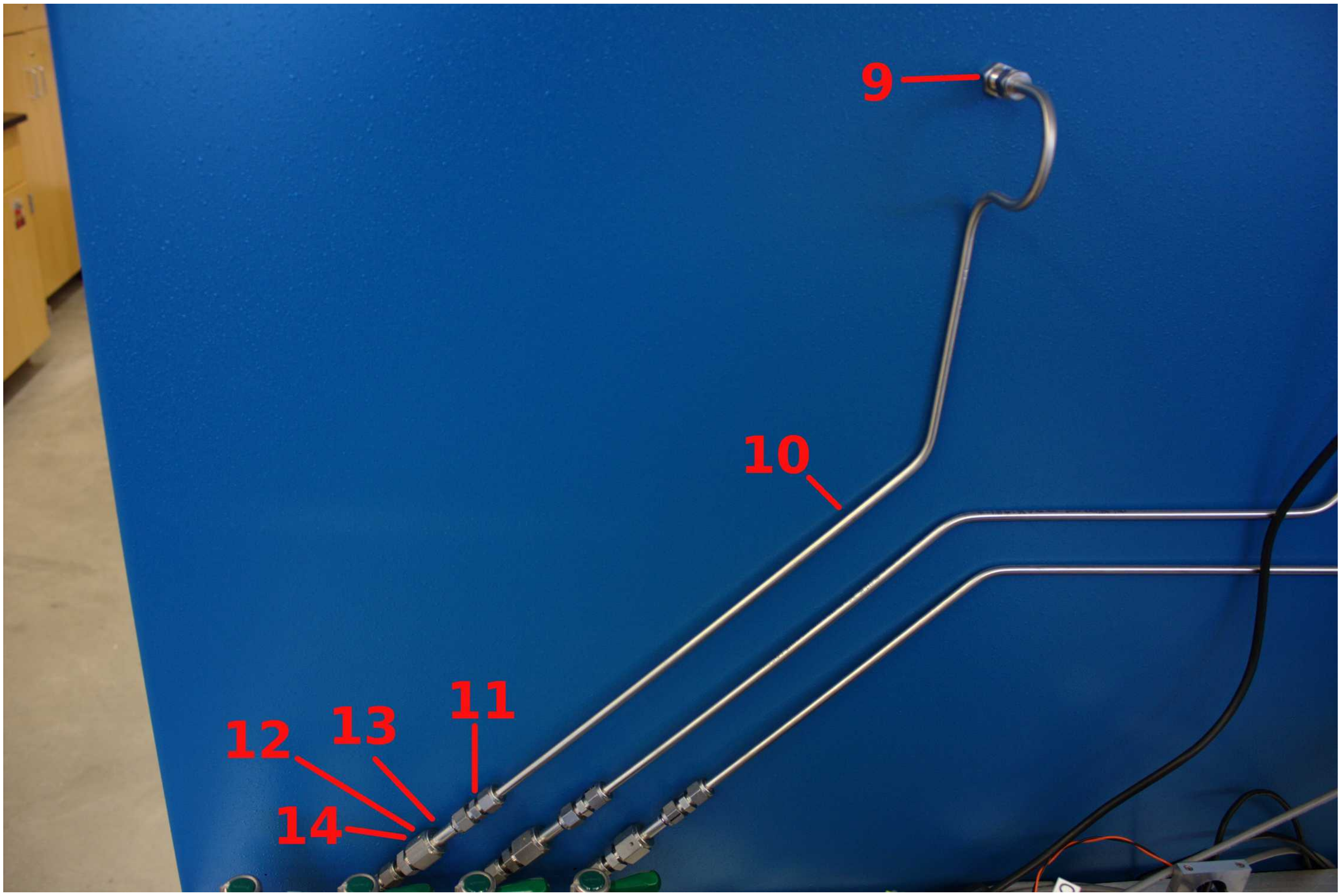

Comments:

- Leave tube adapter connections loose until alignment and spacing is verified.
- Pipe bender needed to make clean bends in tube.
- To disconnect CVD from gas lines, use VCR connection. VCR gasket must be replaced to reattach.

Subsystem 2: Gas filter lines

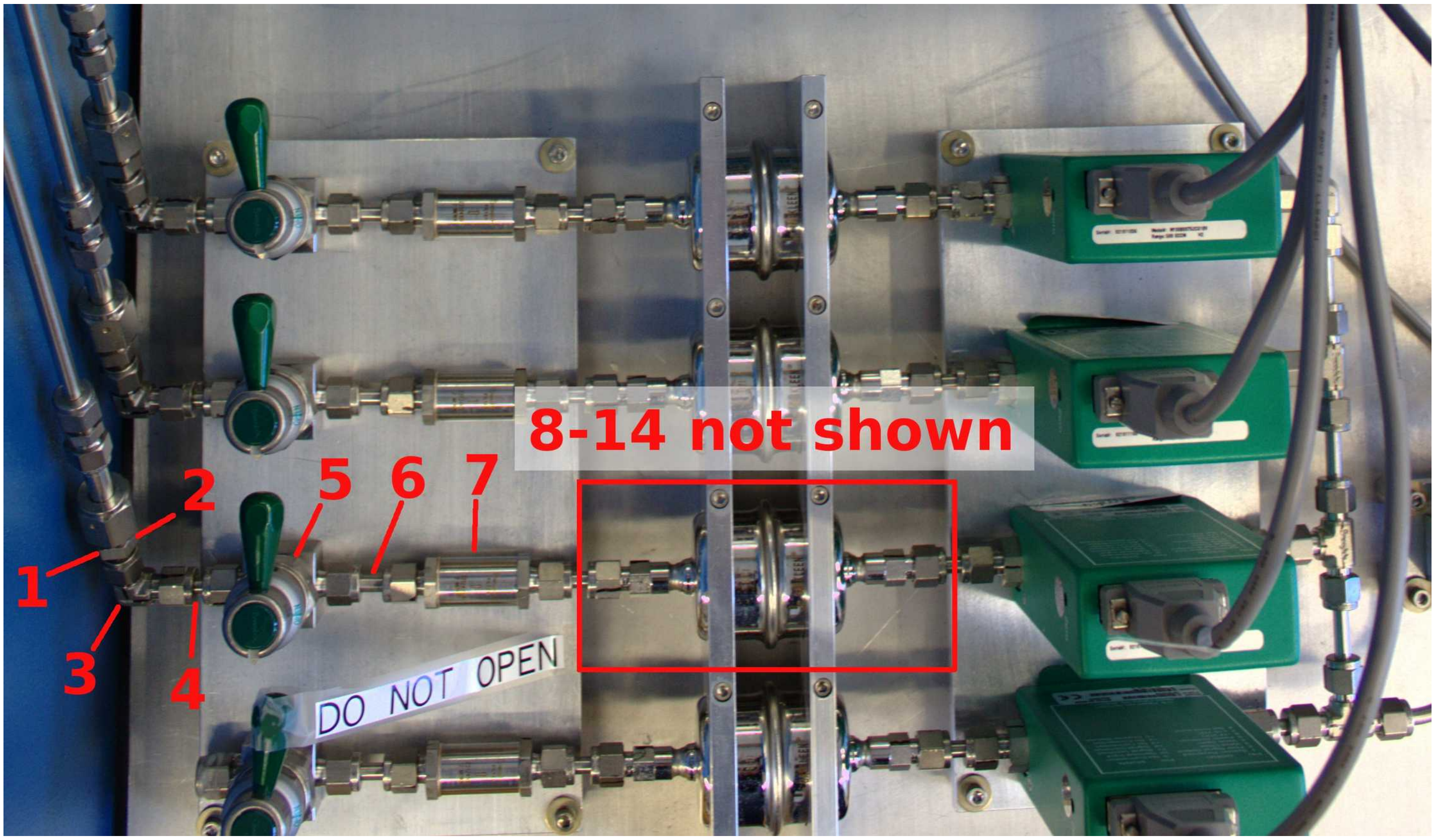

Comments:

- Leave tube adapter connections loose until alignment and spacing is verified.
- Gaskleen filters are shown, but parts list uses Swagelok UHP filters as they are cheaper.
- Diaphragm valves are mounted on support plate, see: *Subsystem 19: Base and supports – Gas filter lines*

Subsystem 3: Mass flow controllers

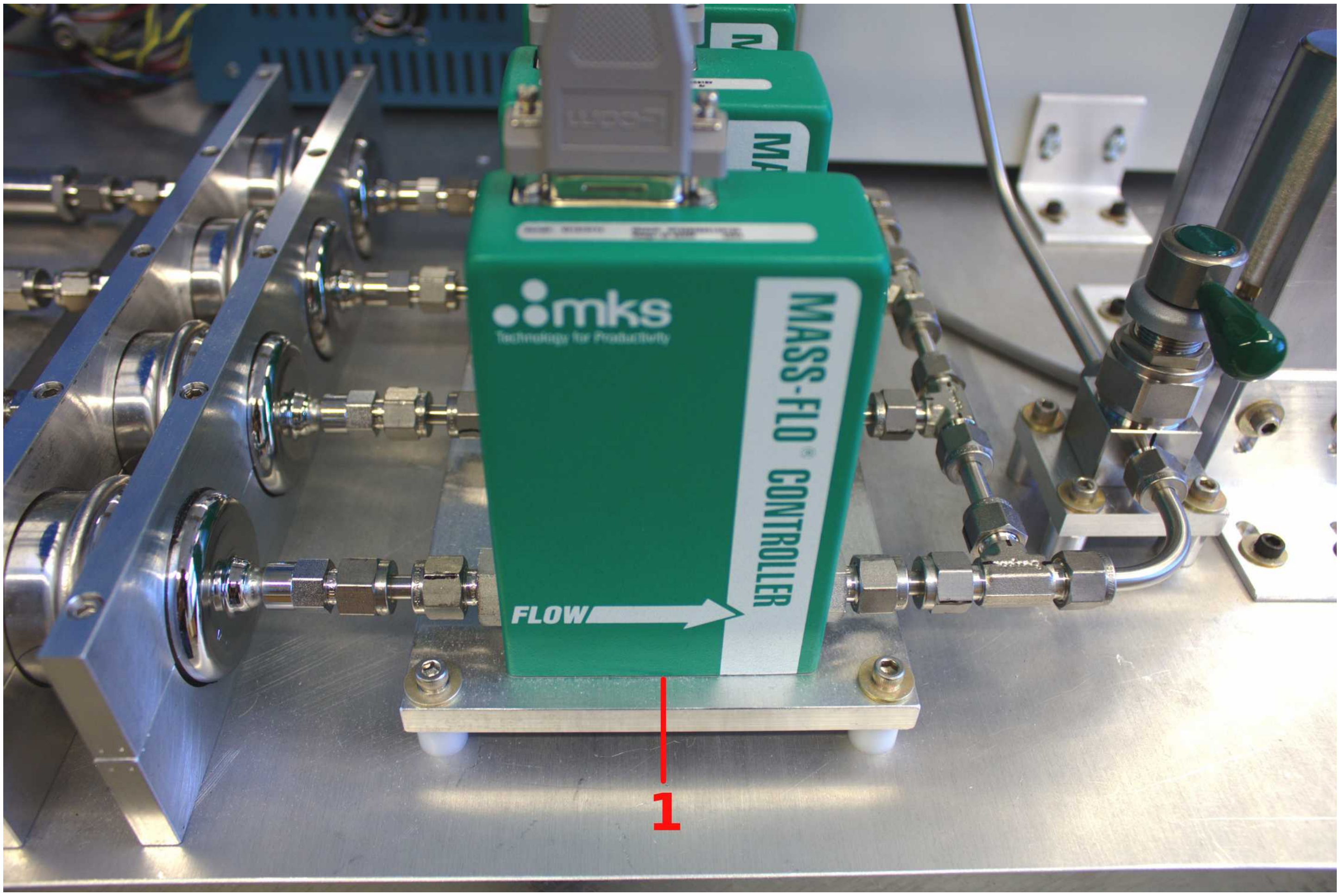

Comments:

- Support: *Subsystem 19: Base and supports – Mass flow controllers*
- Cables: *Subsystem 16: Cables – Mass flow controllers*

Subsystem 4: Gas mixing line

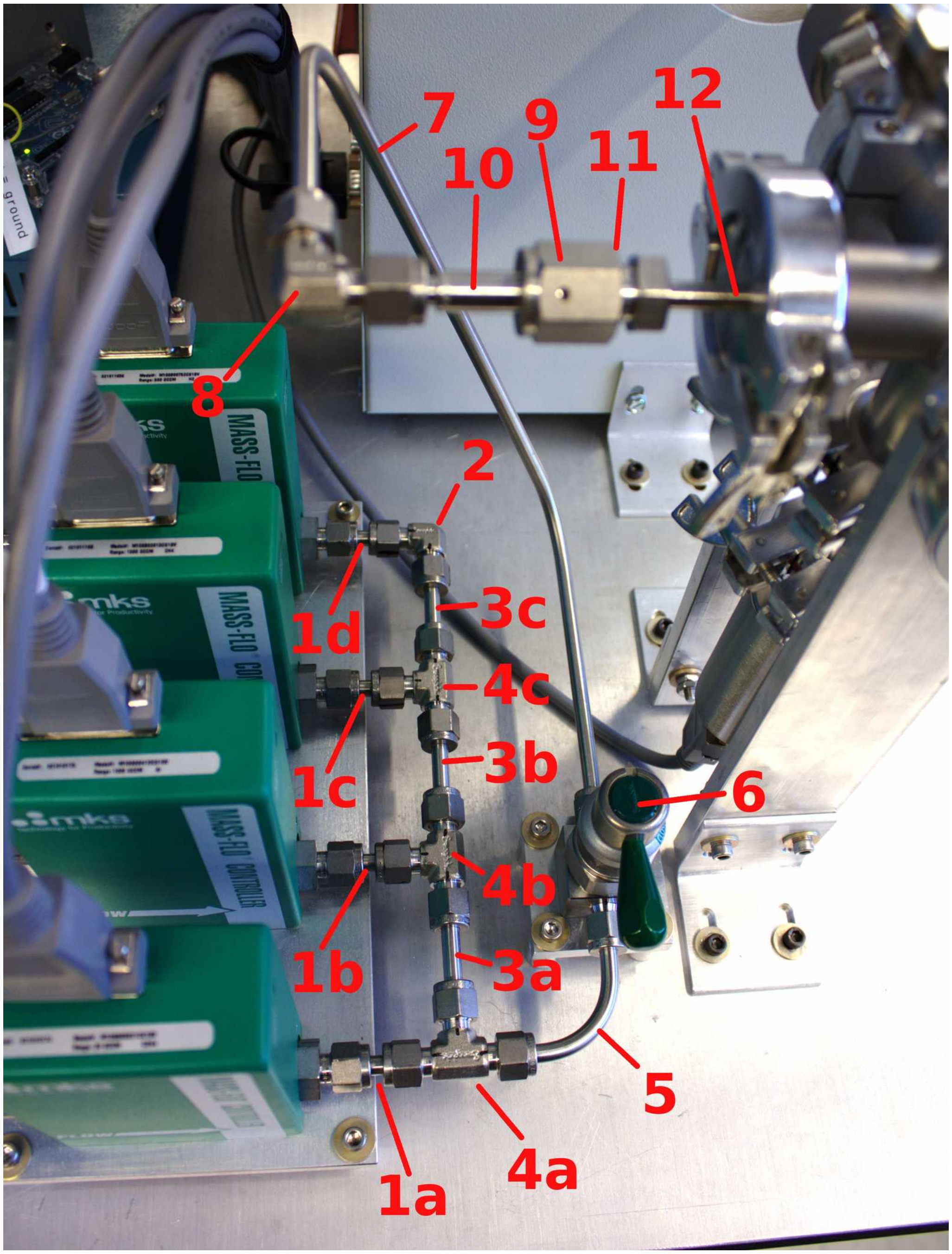

Comments:

- Leave tube adapter connections loose until alignment and spacing is verified.
- Diaphragm valve used for locating leaks.
- To disconnect gas mixing line from intake manifold, use either VCR connection or VCR to KF25 adapter. VCR gasket must be replaced to reattach in the former case.
- Support: *Subsystem 19: Base and supports – Gas mixing line*

Subsystem 5: Intake manifold

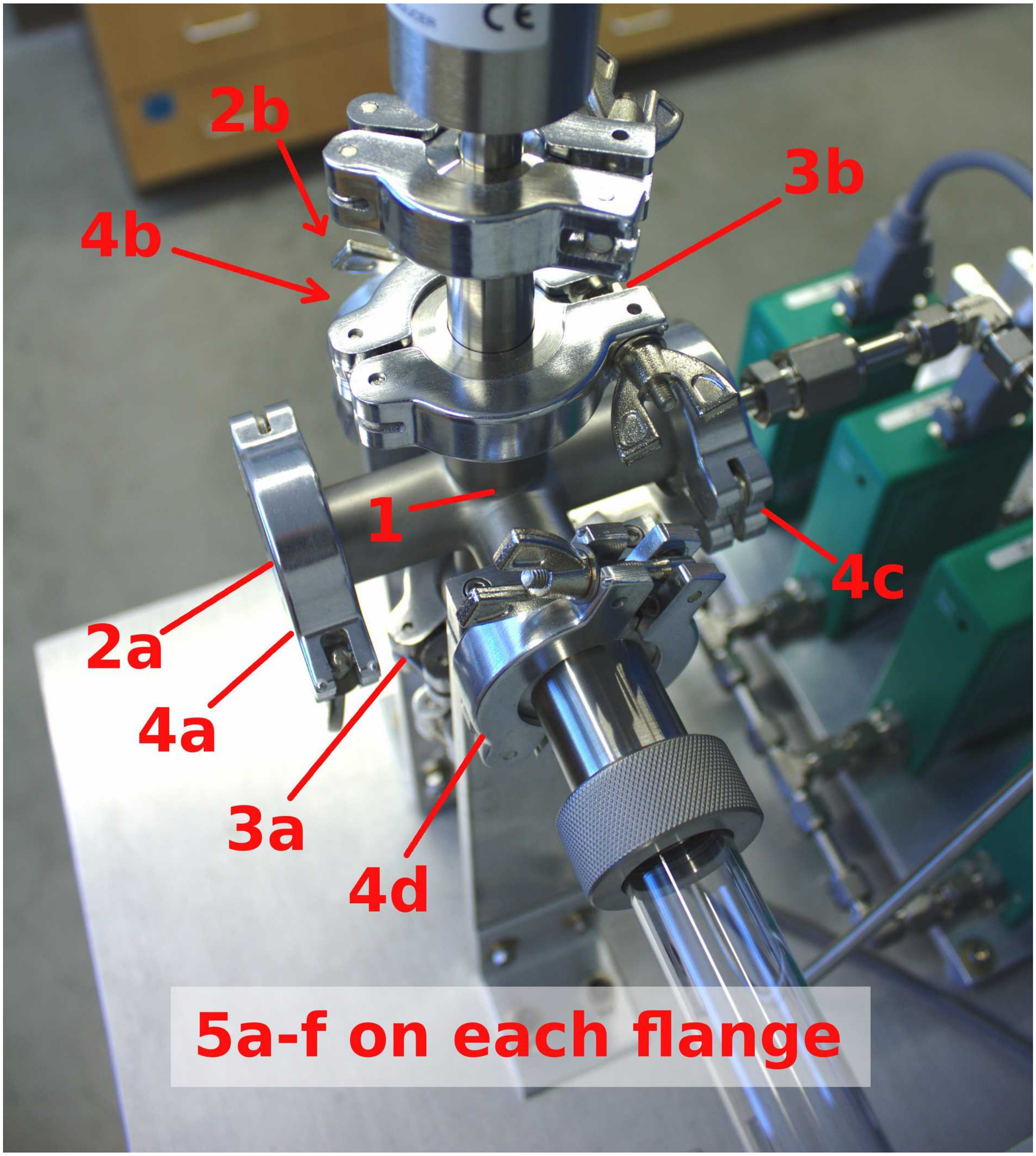

Comments:

- Blank flanges are for possible expansion of system.
- Supports: *Subsystem 19: Base and supports – Intake manifold*

Subsystem 6: Pressure gauges

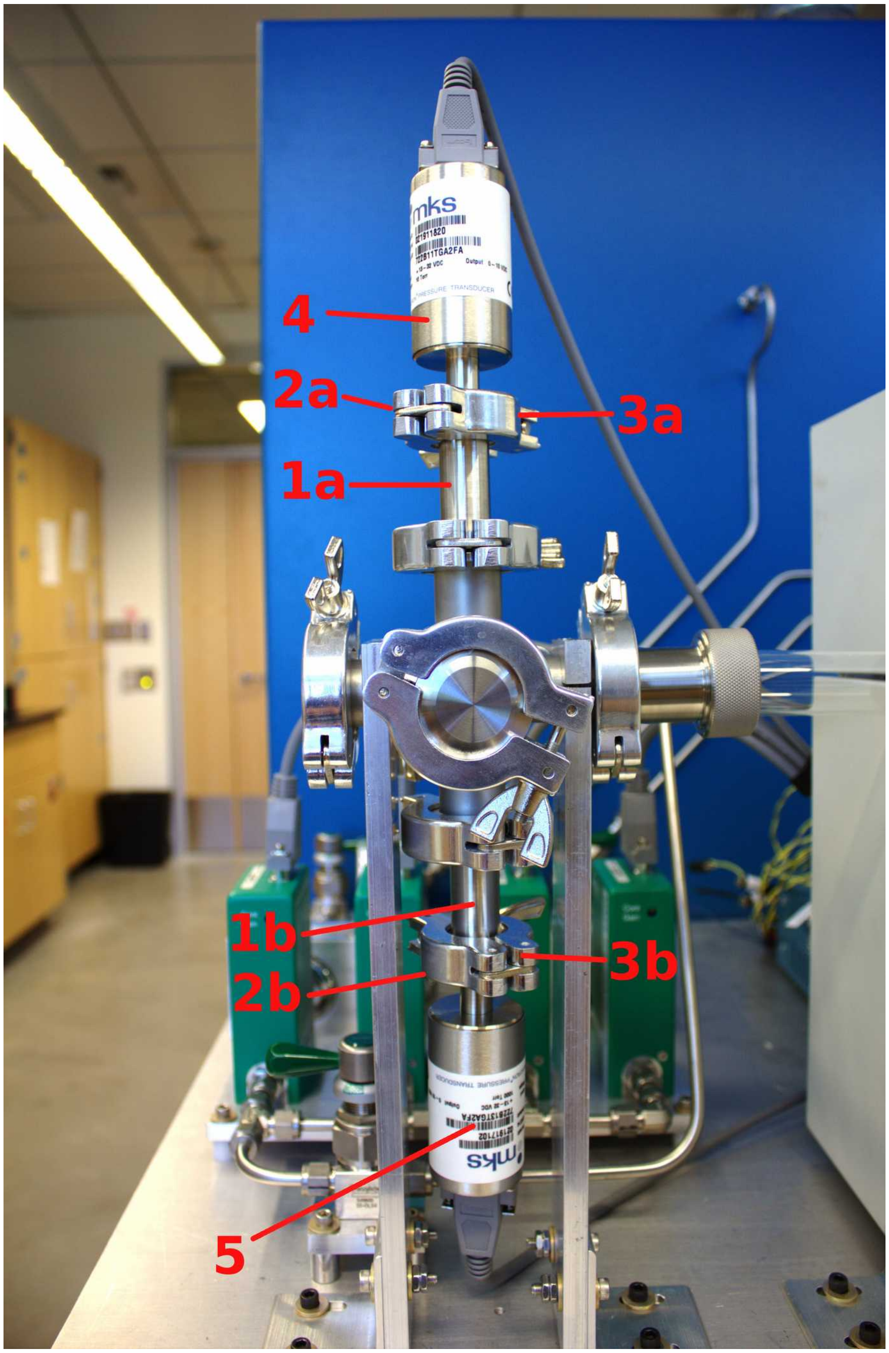

Comments:

- Pressure gauges unnecessary for atmospheric pressure CVD variant.
- Cables: *Subsystem 16: Cables – Pressure gauges*

Subsystem 7: Reaction tube

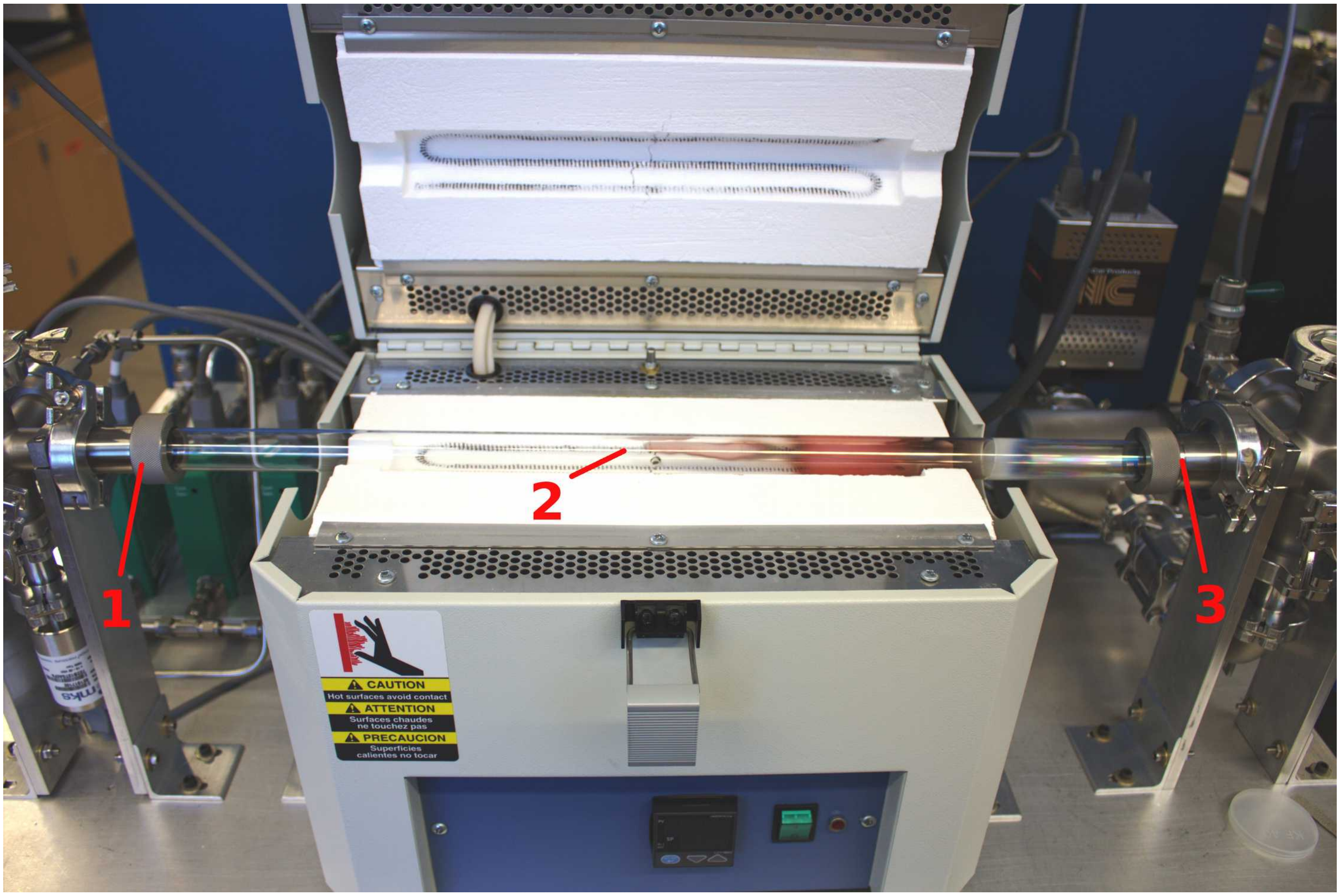

Comments:

- Tubes with fire polished ends are preferred to avoid cracking.
- To remove tube: Loosen quick disconnects (parts 1 and 3), disconnect KF25 cast clamps from quick disconnects, slide quick disconnects toward middle of tube until reaction tube assembly can be lifted away from furnace, slide quick disconnects off of tube.
- To install tube: Do the above in reverse. It might be easier to disassemble each quick disconnect and then place one piece onto the quartz tube at a time as the O-ring can bind when a quick disconnect slides. Be sure that O-ring directly contacts the part of the disconnect with the KF25 flange instead of the nut.
- Avoid over-tightening quick disconnect nut. Once the O-ring is pressed flat against the quartz tube, a seal is formed.

Subsystem 8: Furnace

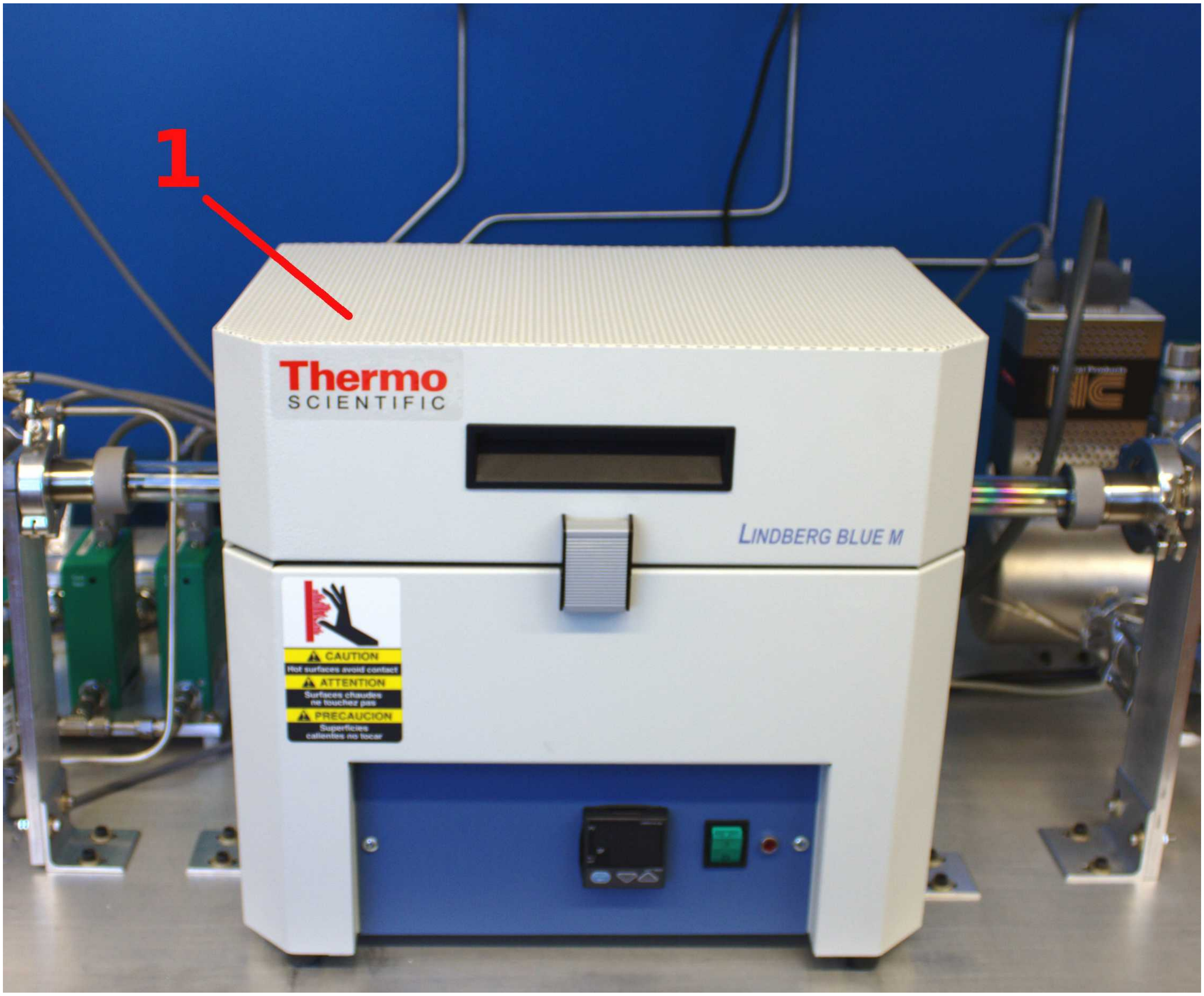

Comments:

- Fumehood may be desired for use of this furnace as insulation degassing may occur when the furnace is hot.

Subsystem 9: Exhaust manifold

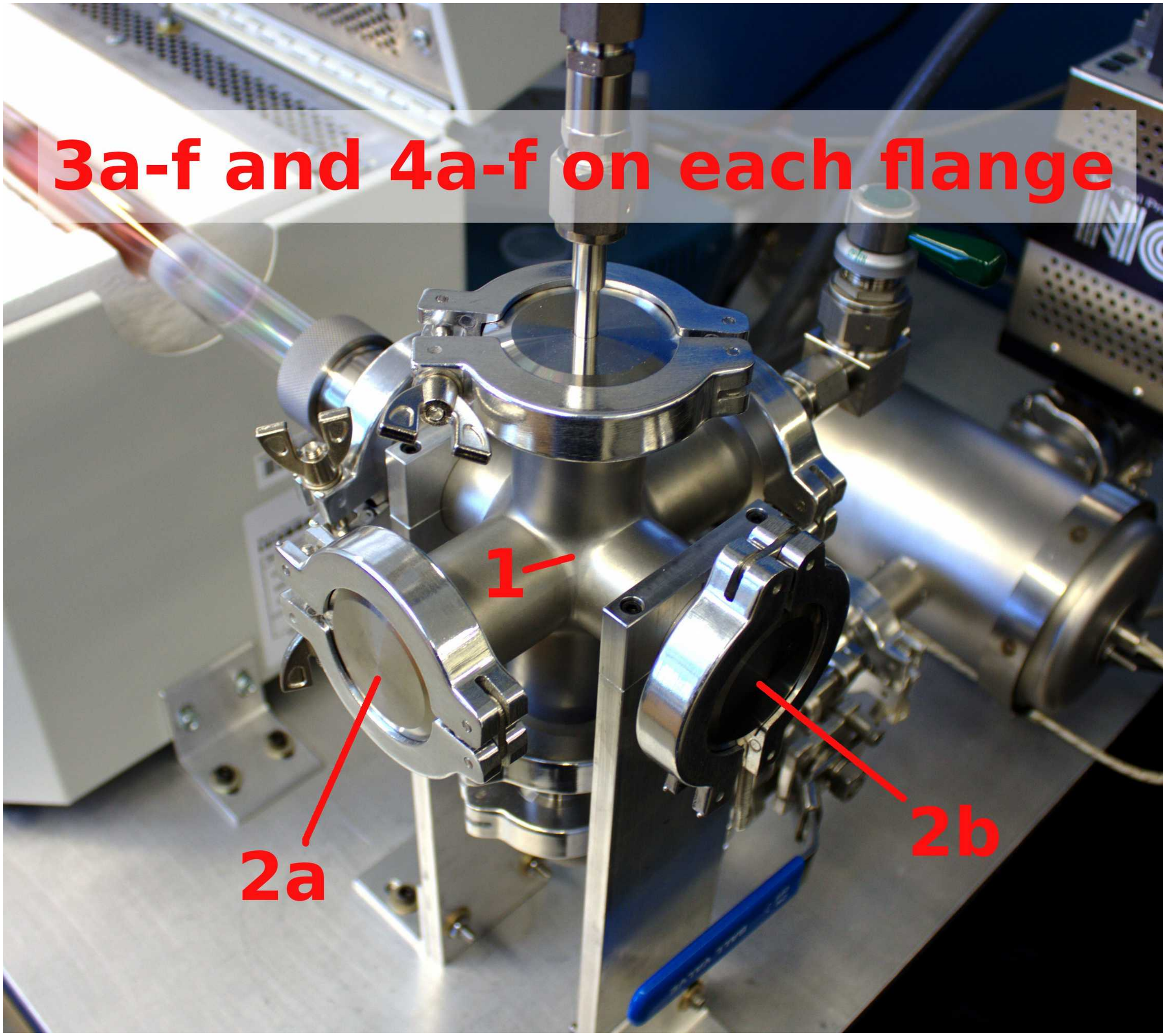

Comments:

- Blank flange in line with the tube (part 2b) is used for sample insertion and retrieval.
- Remaining blank flange (part 2a) reserved for future system additions.

Subsystem 10: Overpressure relief

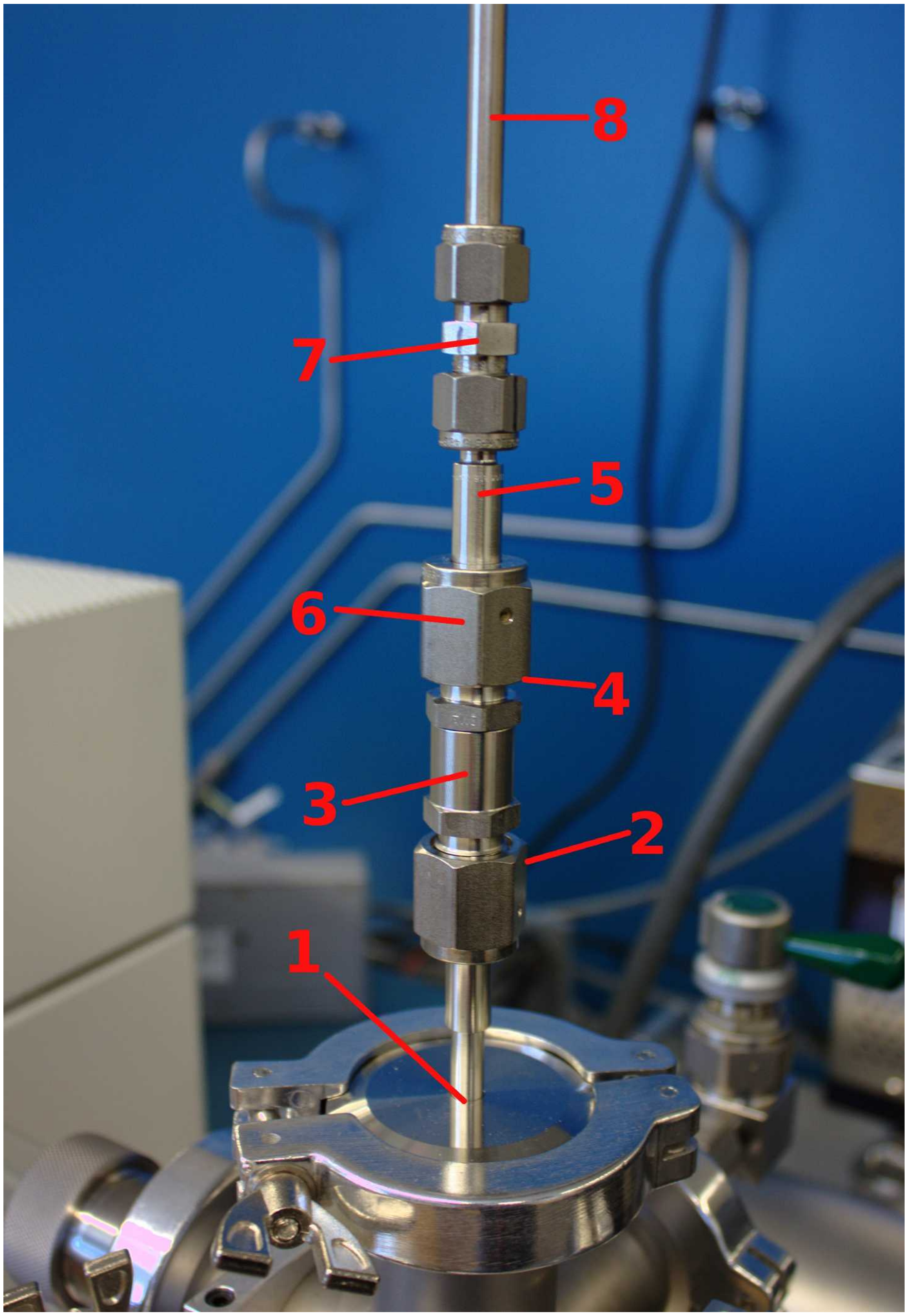

Comments:

- Overpressure relief valve should not be used for hot exhaust as it can become clogged, compromising system seal.

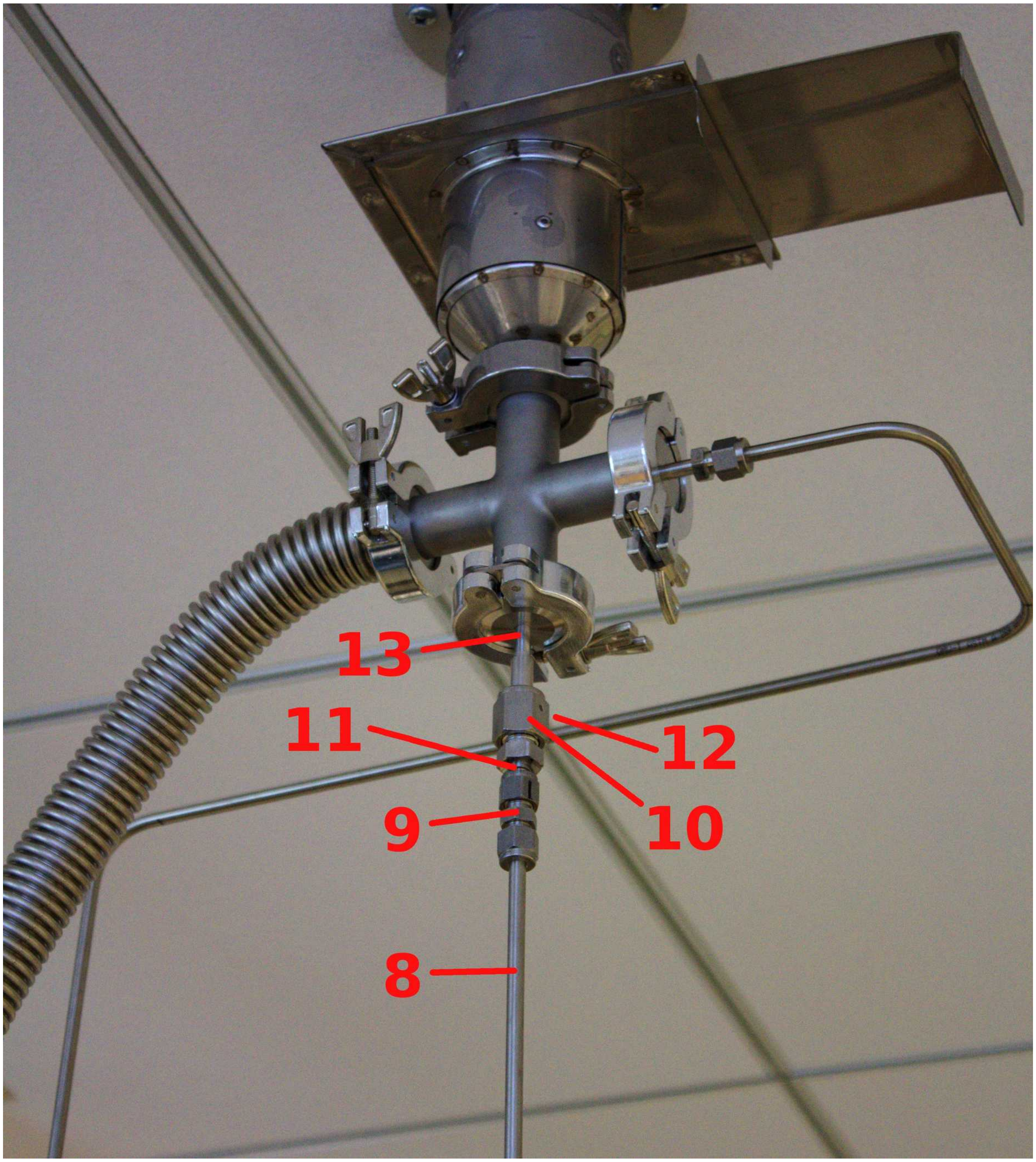

Comments:

- Overpressure relief line should not go to a vacuum line as that would make CVD runs above 1 psi impossible. Instead it should go to an exhaust snorkel that is only slightly below atmospheric pressure.

Subsystem 11: Vent valve

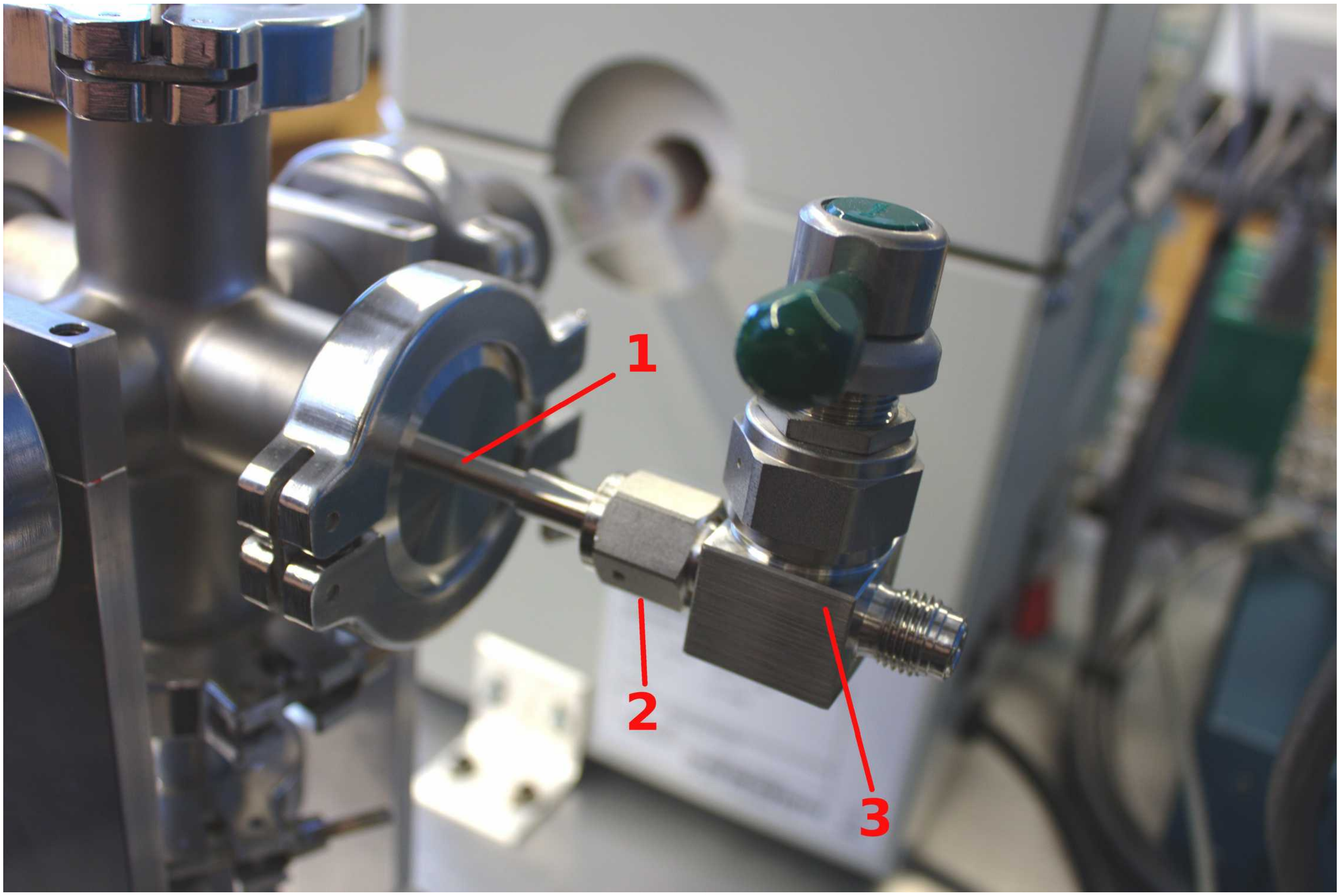

Comments:

- Vent valve is only rarely used as CVD will generally be refilled with argon to atmospheric pressure at the end of runs.

Subsystem 12: Exhaust line

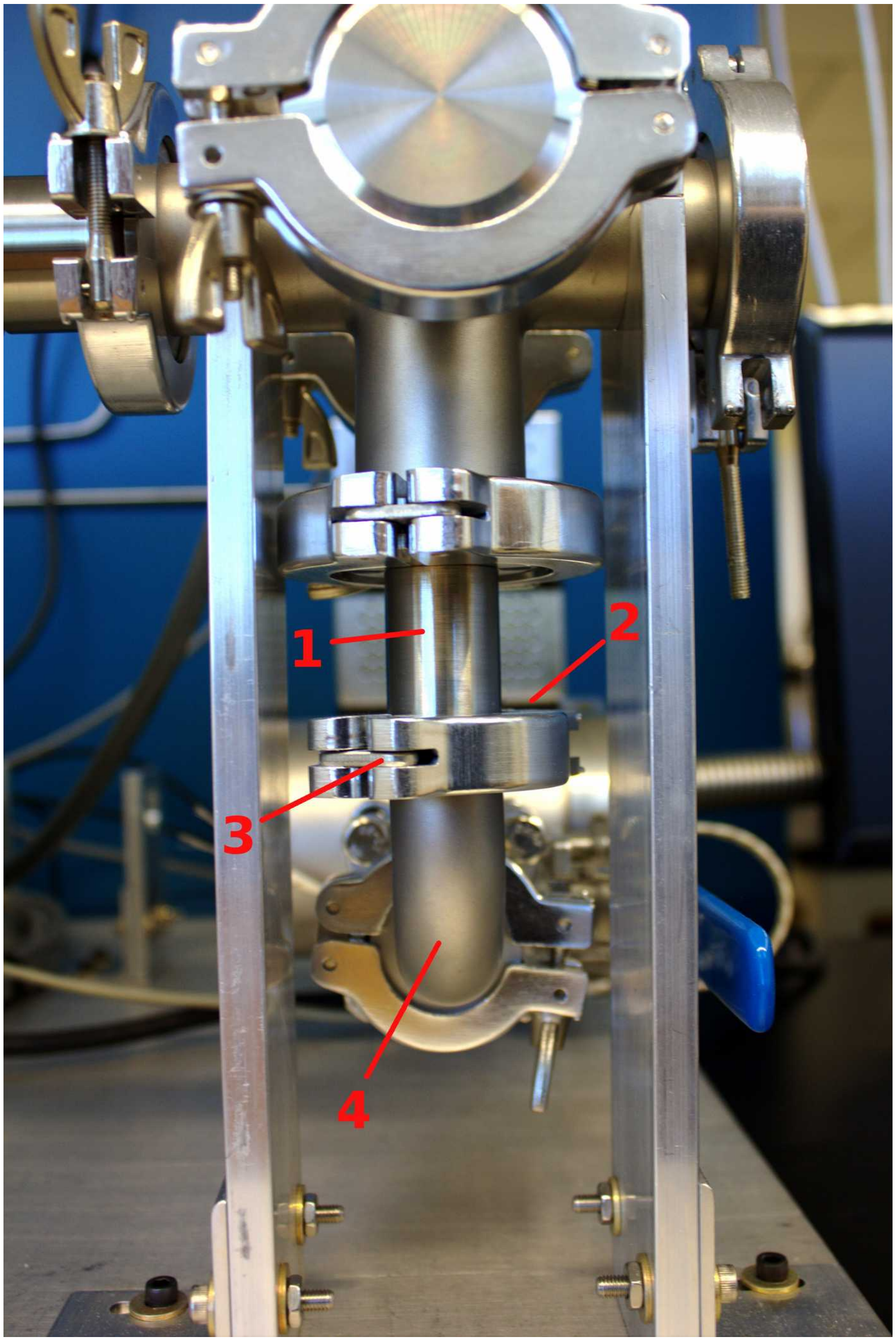

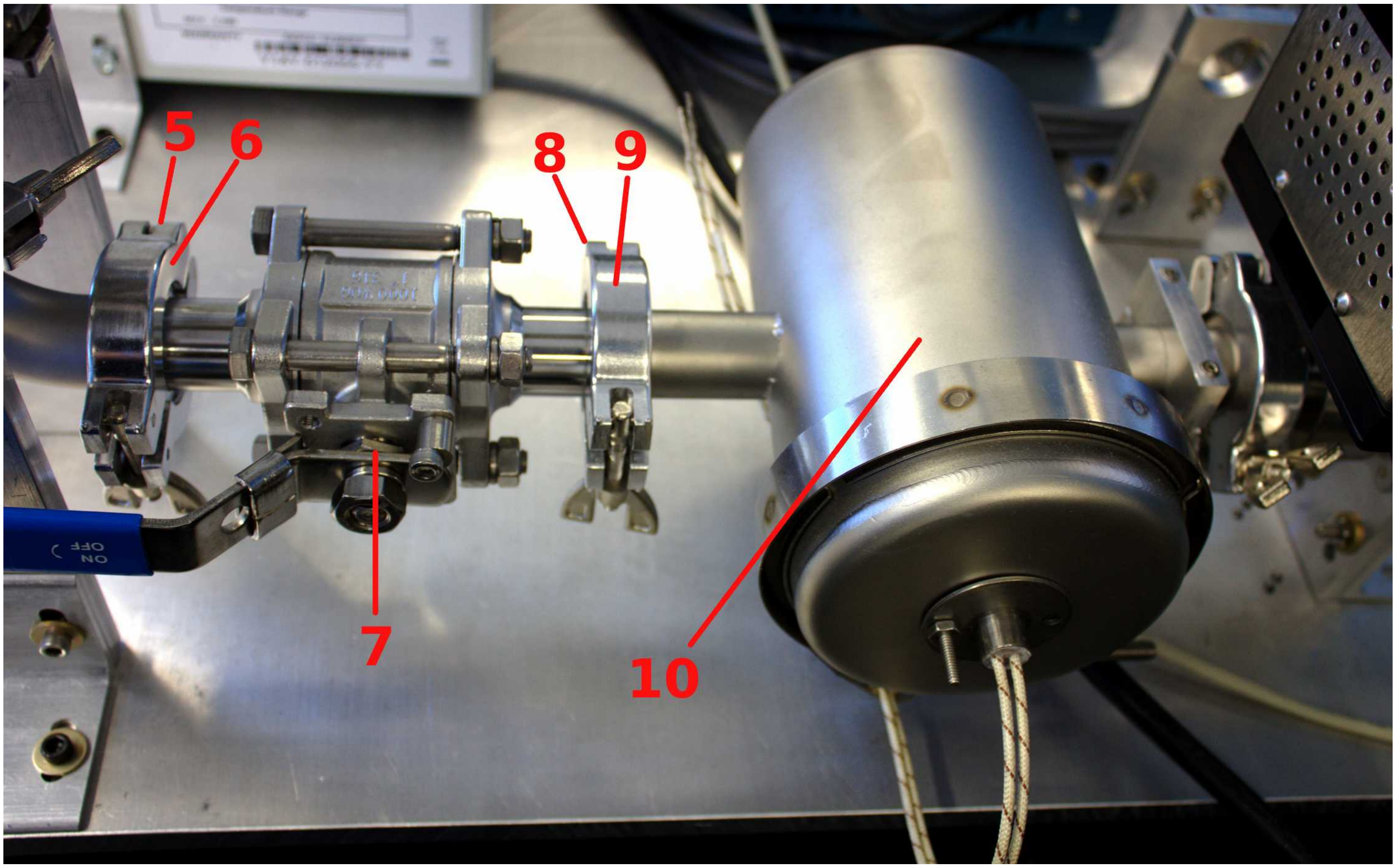

Comments:

- Ball valve (part 7) useful for locating leaks.
- Avoid accumulating incompatible wastes in molecular sieve (part 10).

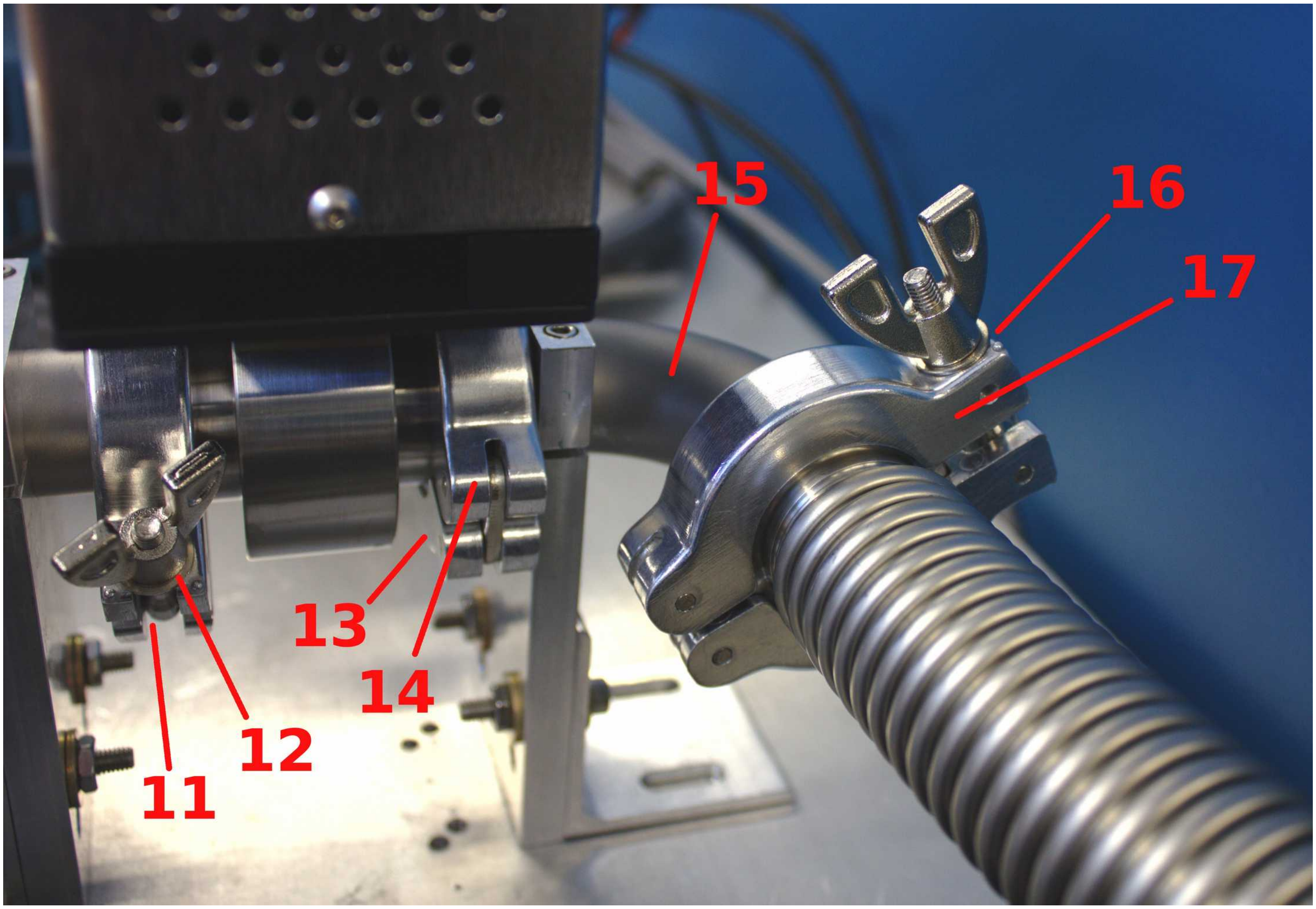

Comments:

- Supports: *Subsystem 19: Base and supports – Exhaust line*

Subsystem 13: Butterfly valve

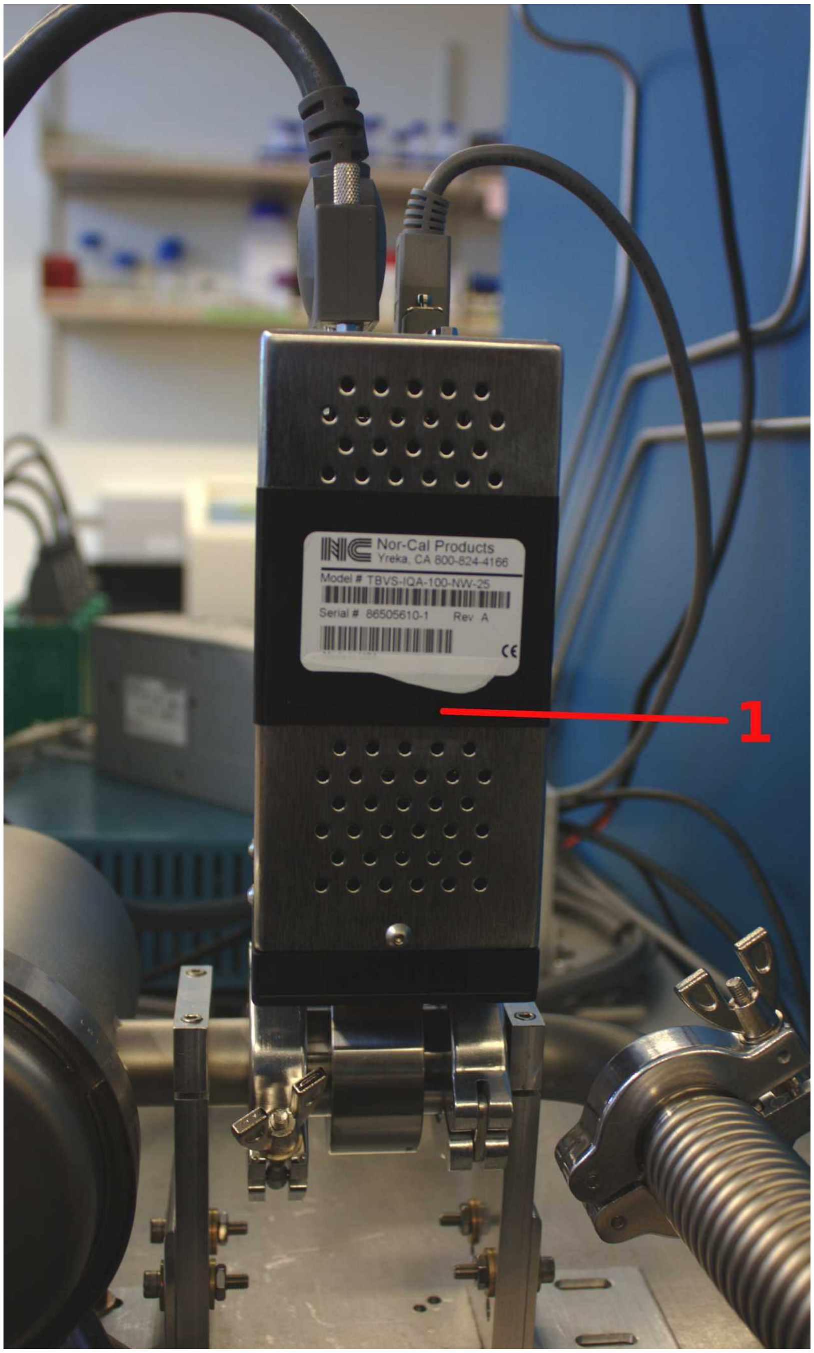

Comments:

- Butterfly valve unnecessary for atmospheric pressure CVD variant.
- 'Open', 'Close' buttons will generally not work after valve has previously been remotely controlled without being power cycled.
- Cables: *Subsystem 16: Cables – Butterfly valve*

Subsystem 14: Vacuum pump

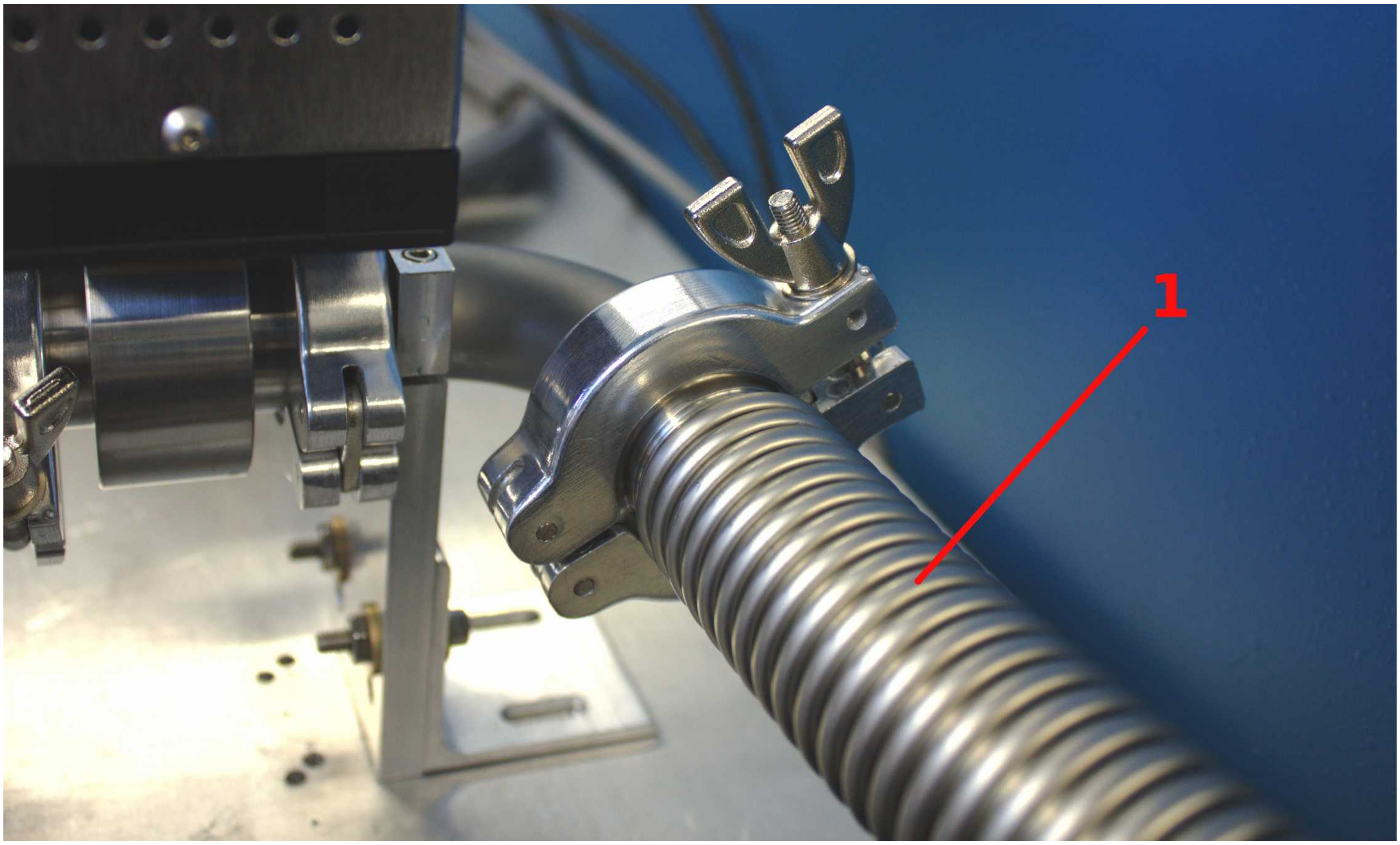

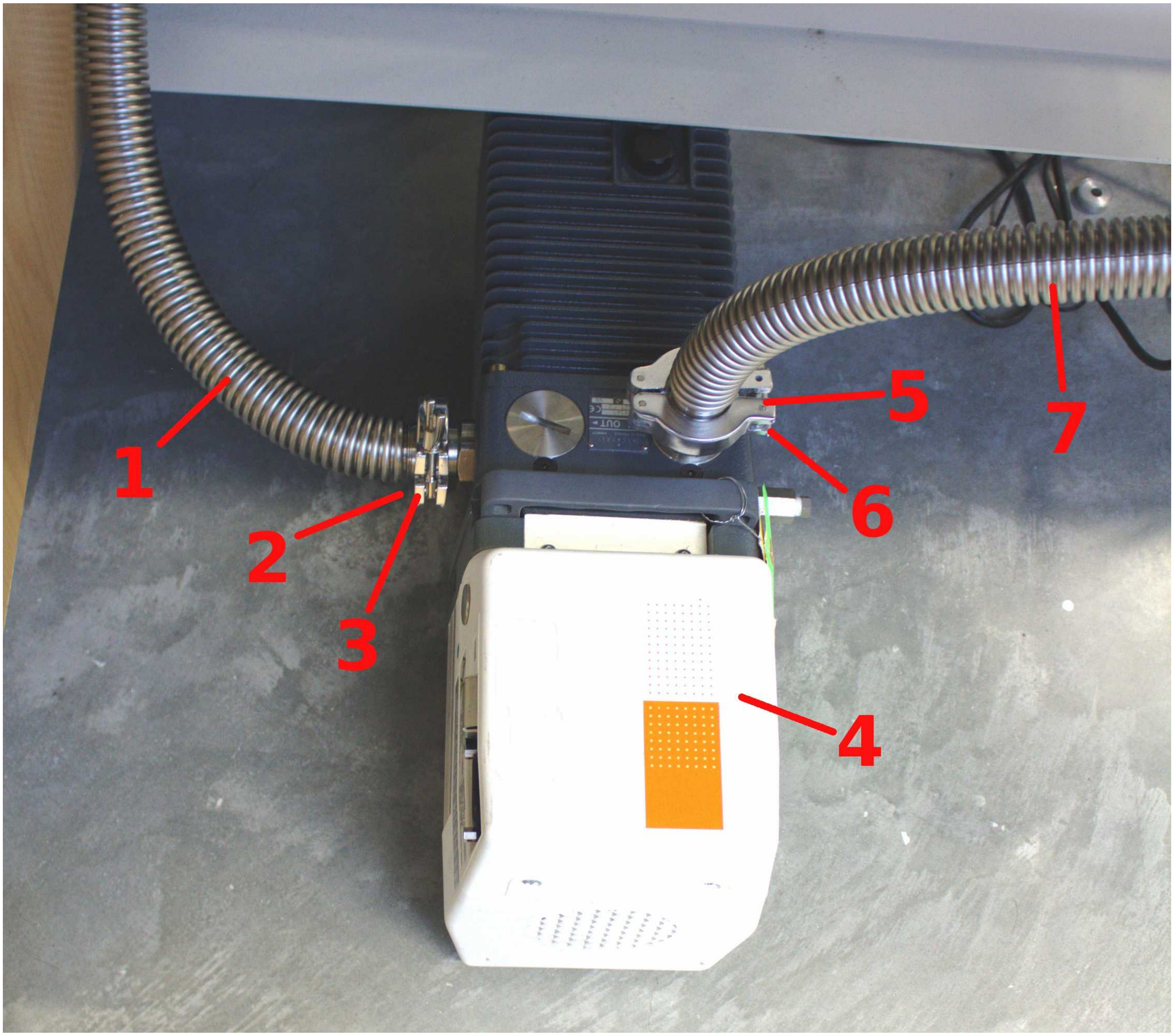

Comments:

- Molecular sieve in exhaust line and butterfly valve will generally prevent back-flow of vacuum pump fluid aerosol.

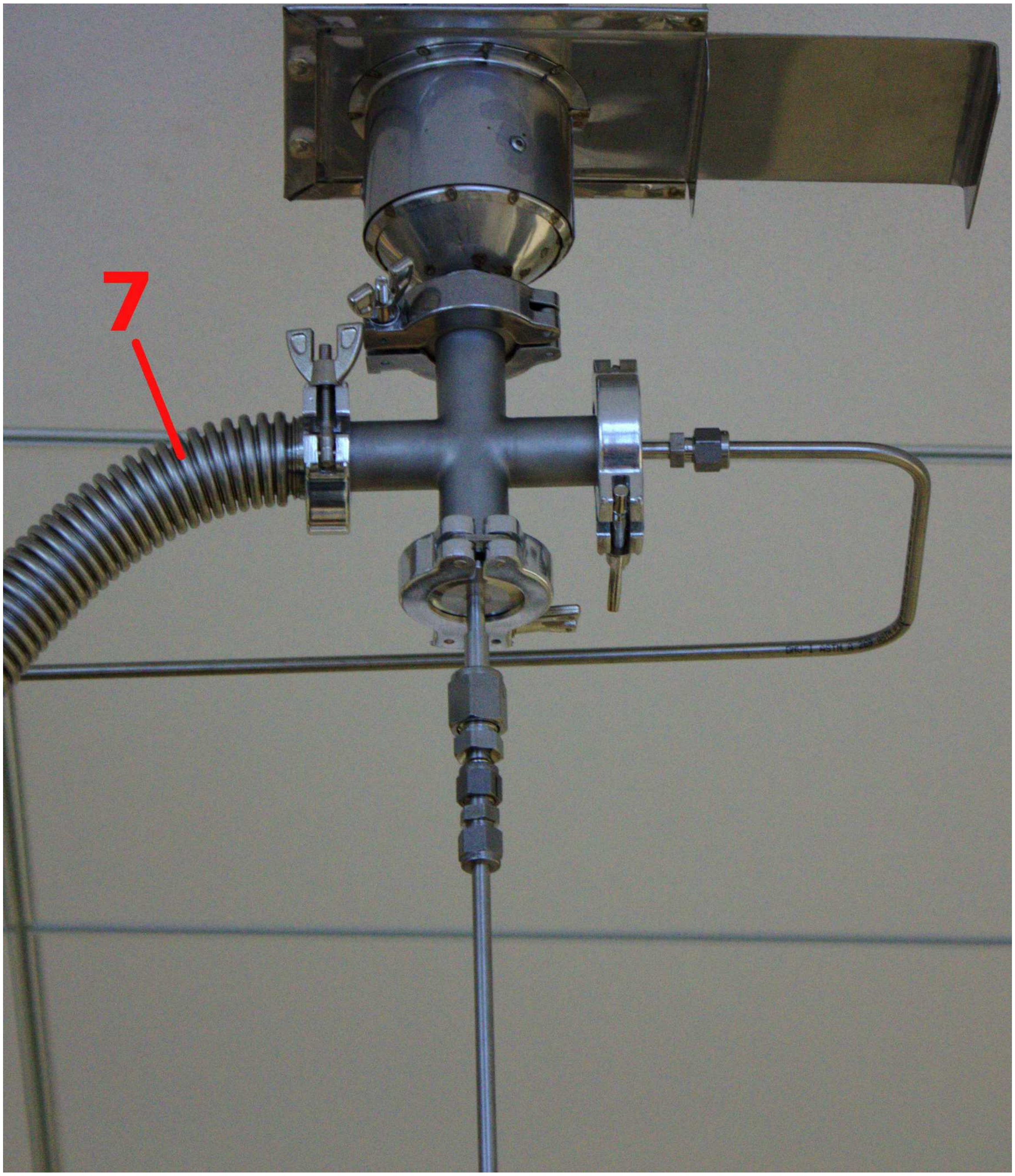

7

Subsystem 15: Snorkel manifold

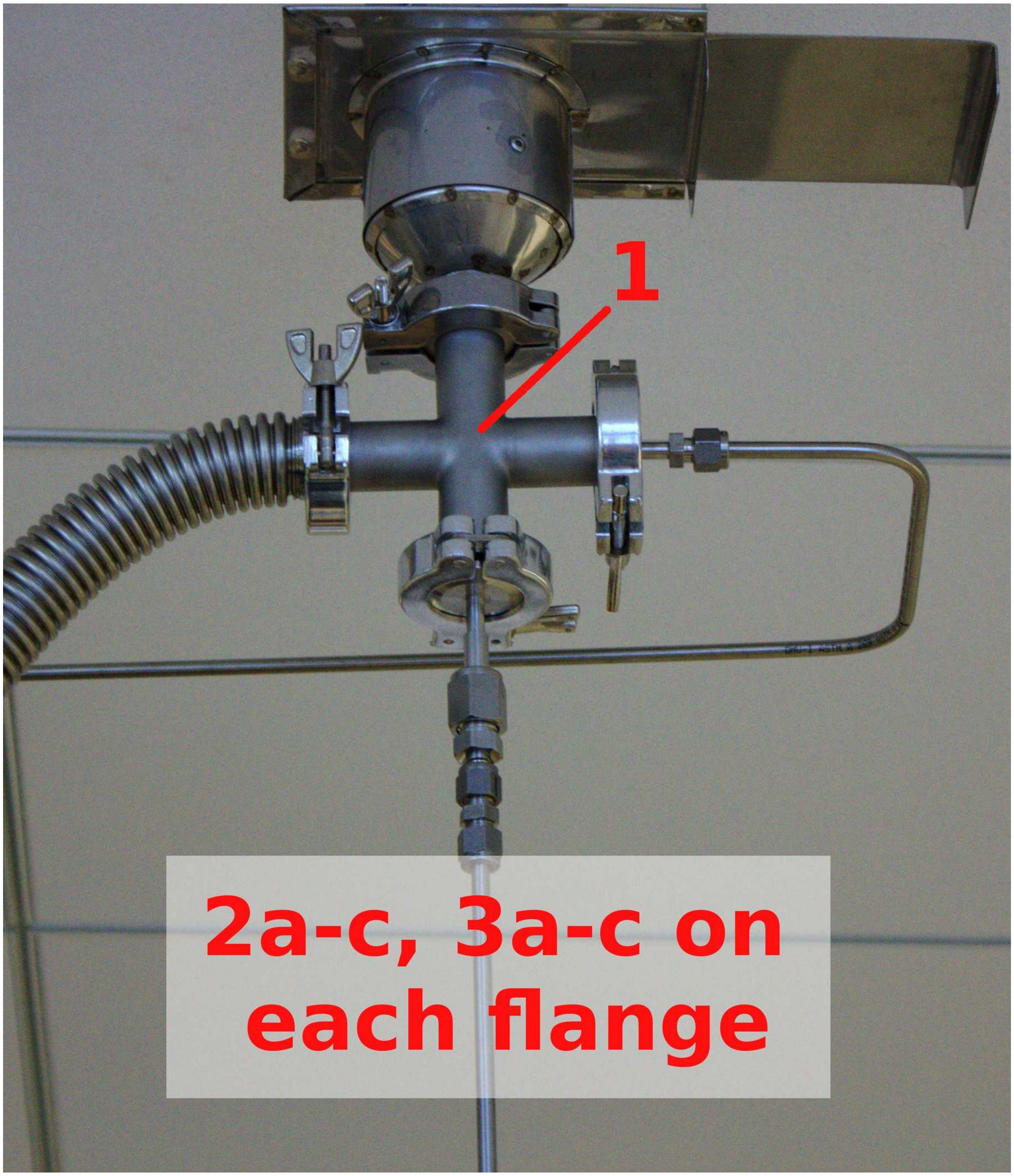

Comments:

- Exhaust snorkel should be only slightly below atmospheric pressure as overpressure relief valve will pop at a 1 psi differential.
- 4-way cross shown above instead of the Tee indicated in the parts list. ¼” line on the right side is exhaust from a different machine.

Subsystem 16: Cables – Mass flow controllers

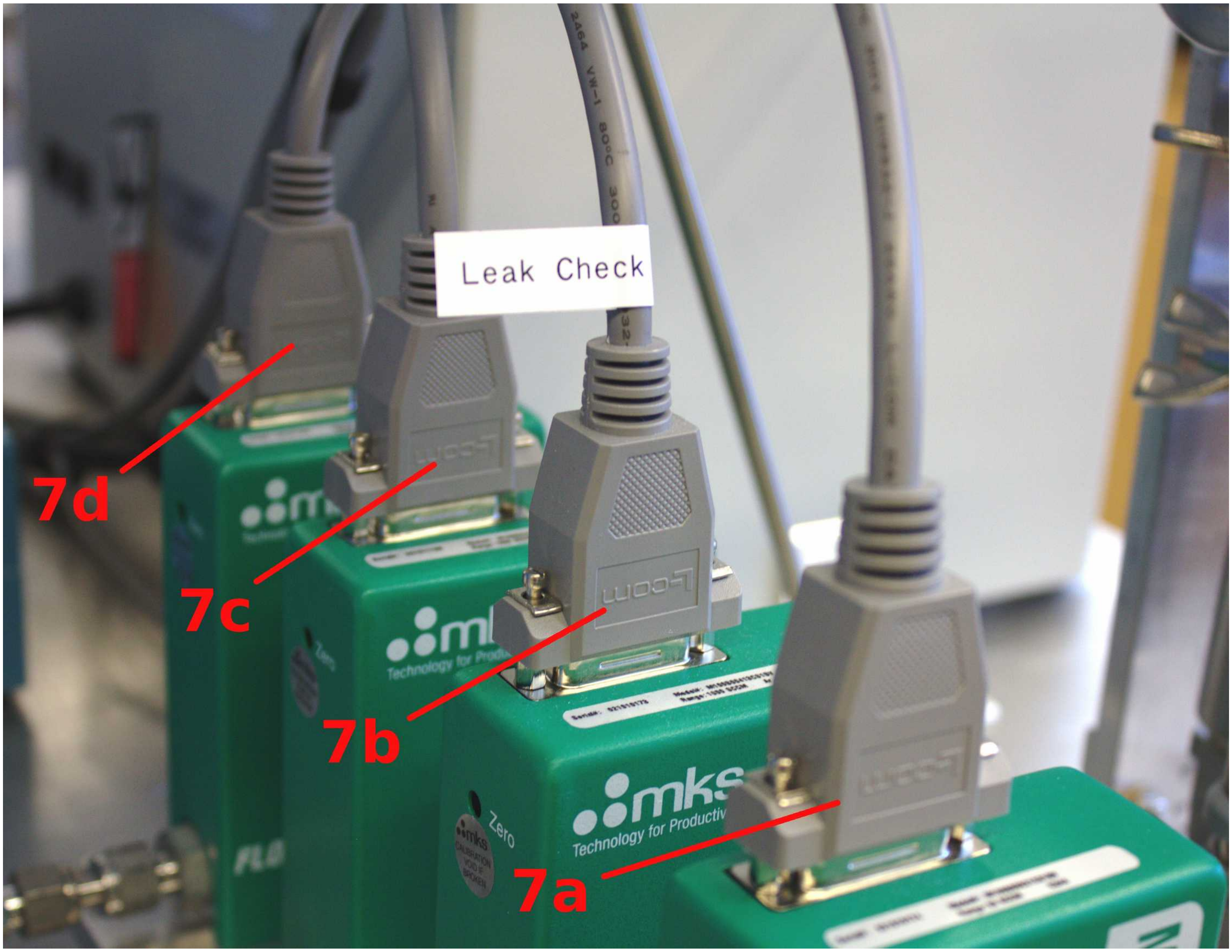

Comments:

- 'Leak Check' cable has additional wires on other end that can be connected to set the MFC valve to open or closed. This allows one to use the vacuum pump and pressure gauges to check each gas line for leaks all the way back to the cylinder.
    - To check a gas line for leaks: Unplug all MFC cables (for MFCs that are normally closed), attach leak check cable to MFC of gas line to be checked, close cylinder valve for that gas line, set MFC to open via cable, verify seal of gas line by checking if the same minimum system pressure can be attained with the MFC open as with it closed. Alternatively, the rate of increase of pressure can be checked by closing the butterfly valve.

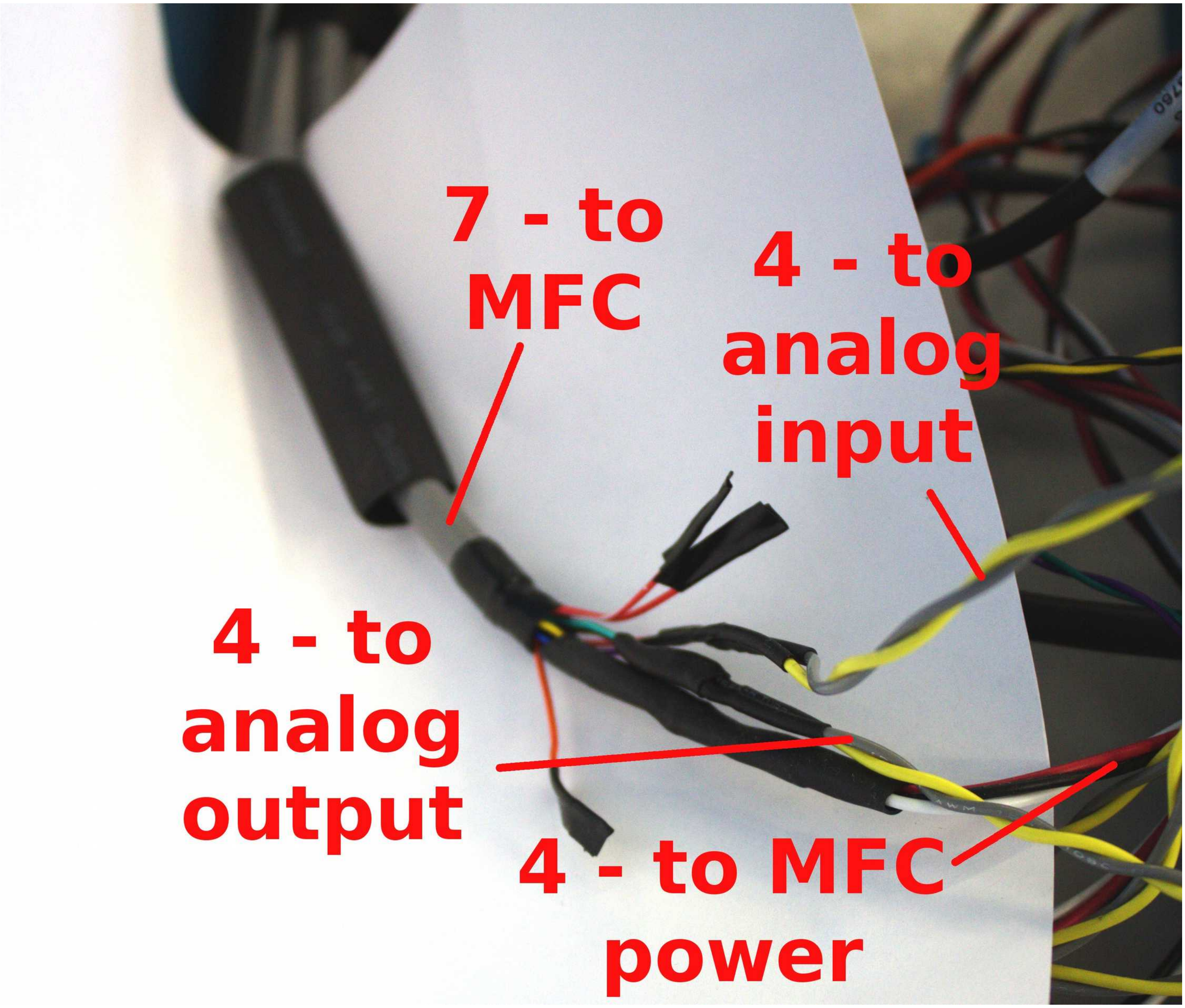

Comments:

- See the wiring diagram of your specific MFC for required cable pinout. For MKS M100B013##CS1BV mass flow controllers, see *Appendix A: Mass flow controller cables*

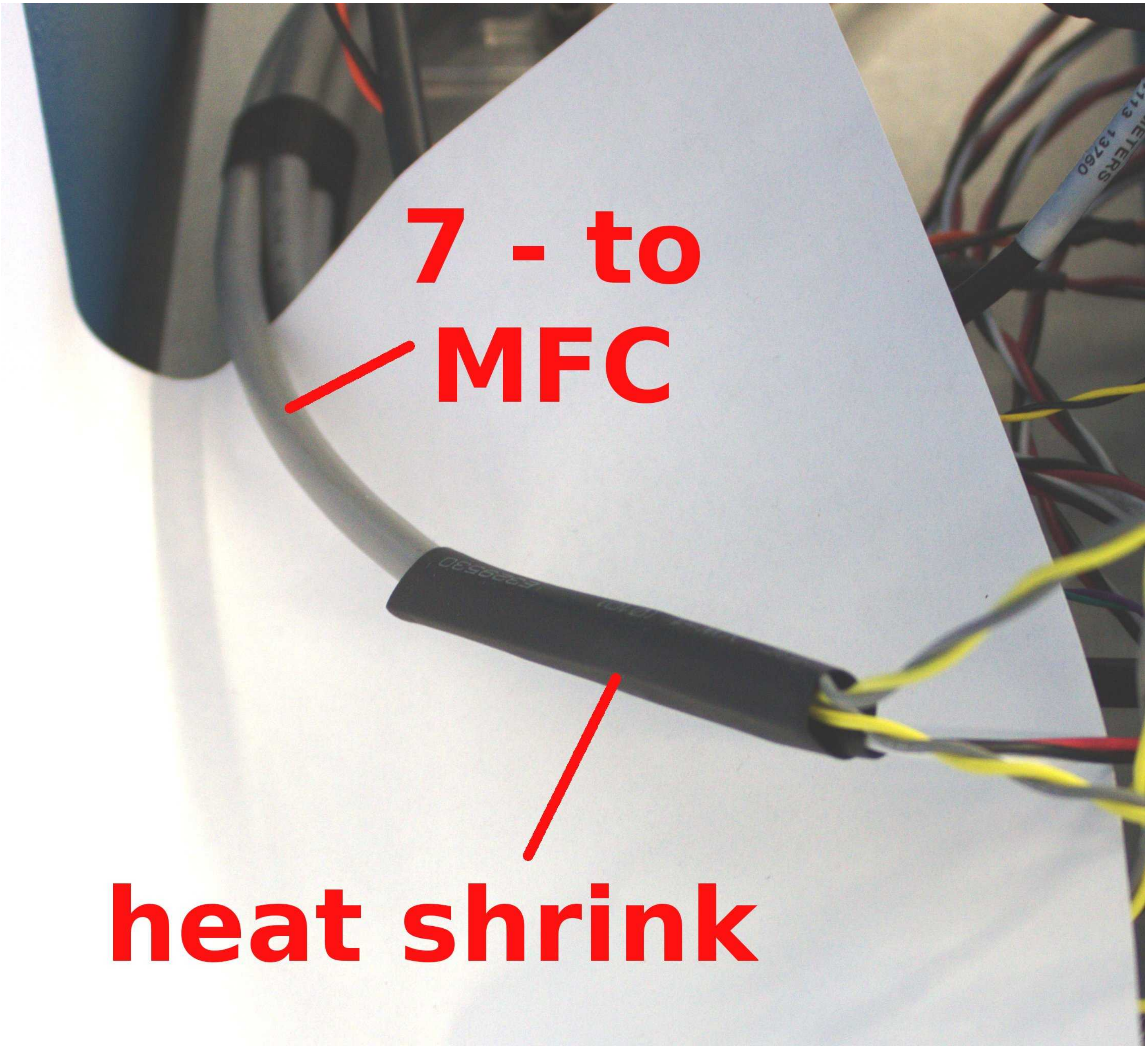

Comments:

- Regular cable.

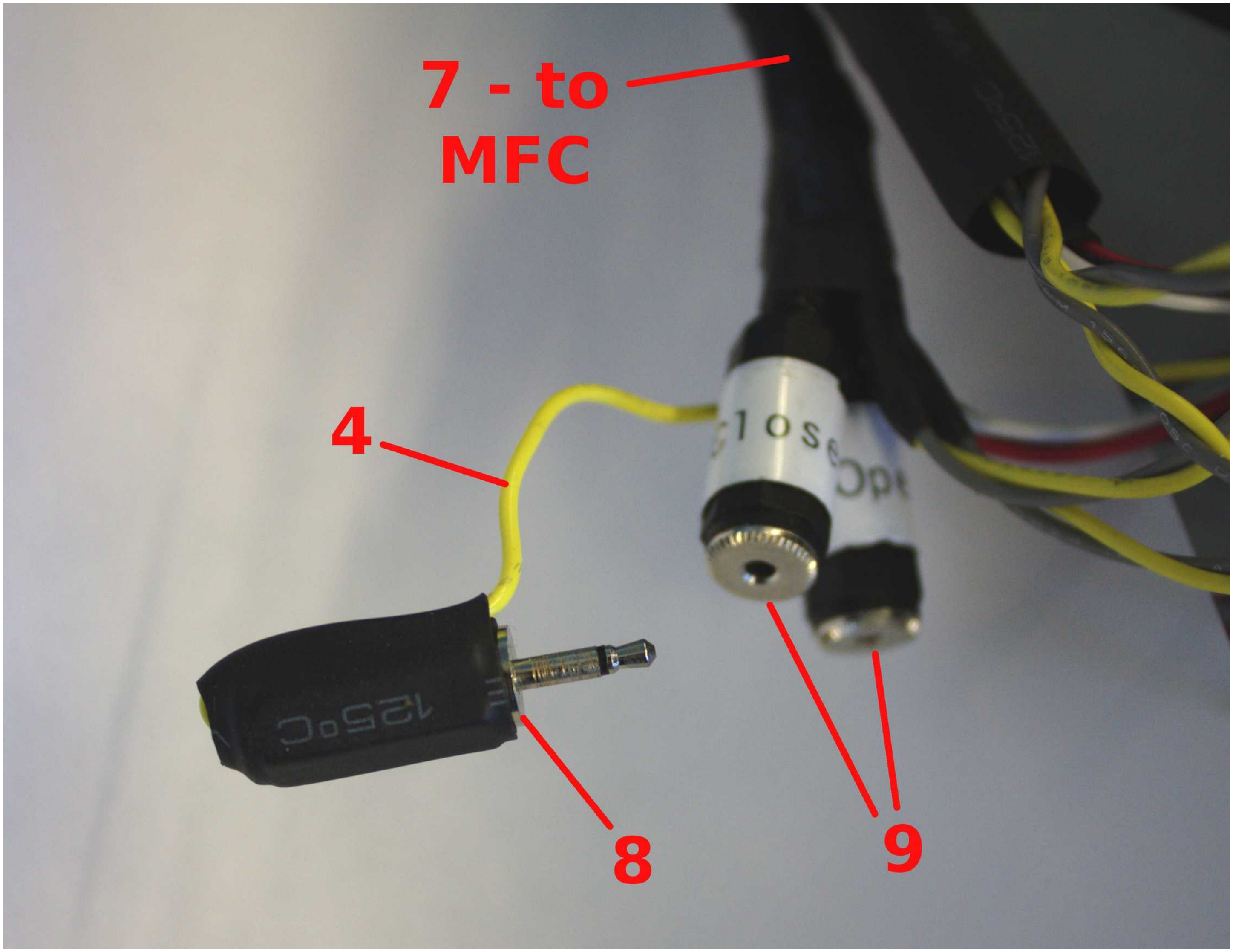

Comments:

- 'Leak Check' cable.

Subsystem 16: Cables – Pressure gauges

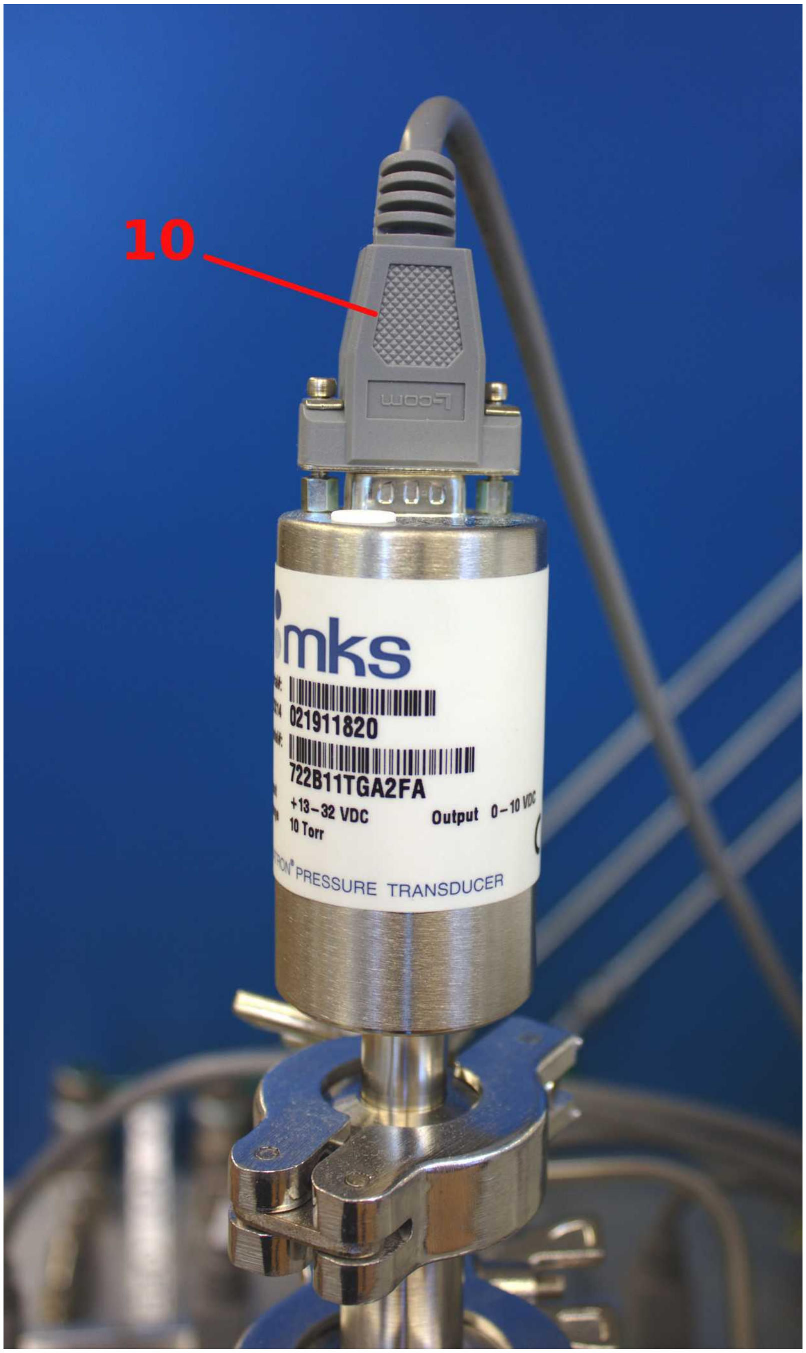

Comments:

- Pinout: *Appendix A: Pressure gauge cabless*

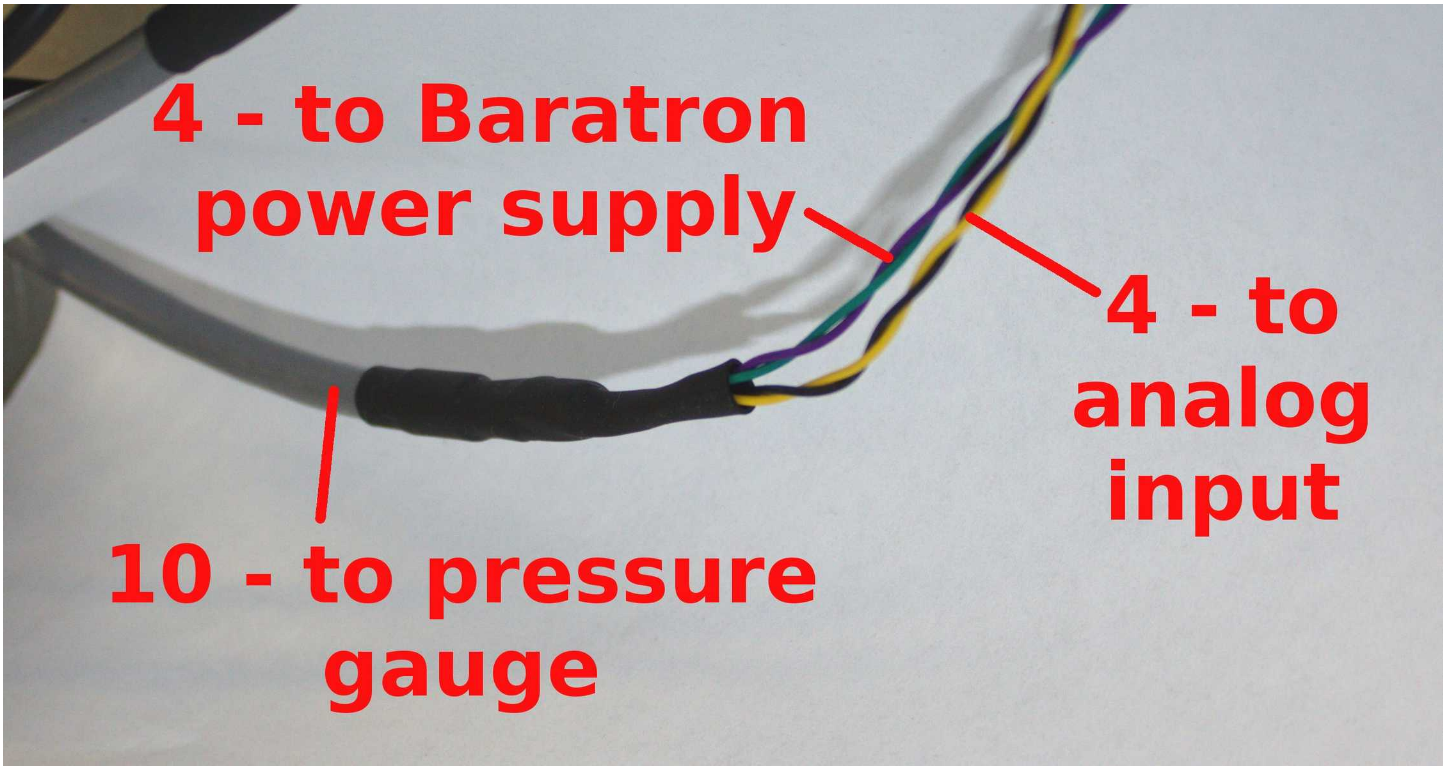
4 - to Baratron power supply
4 - to analog input
10 - to pressure gauge

Subsystem 16: Cables – Furnace

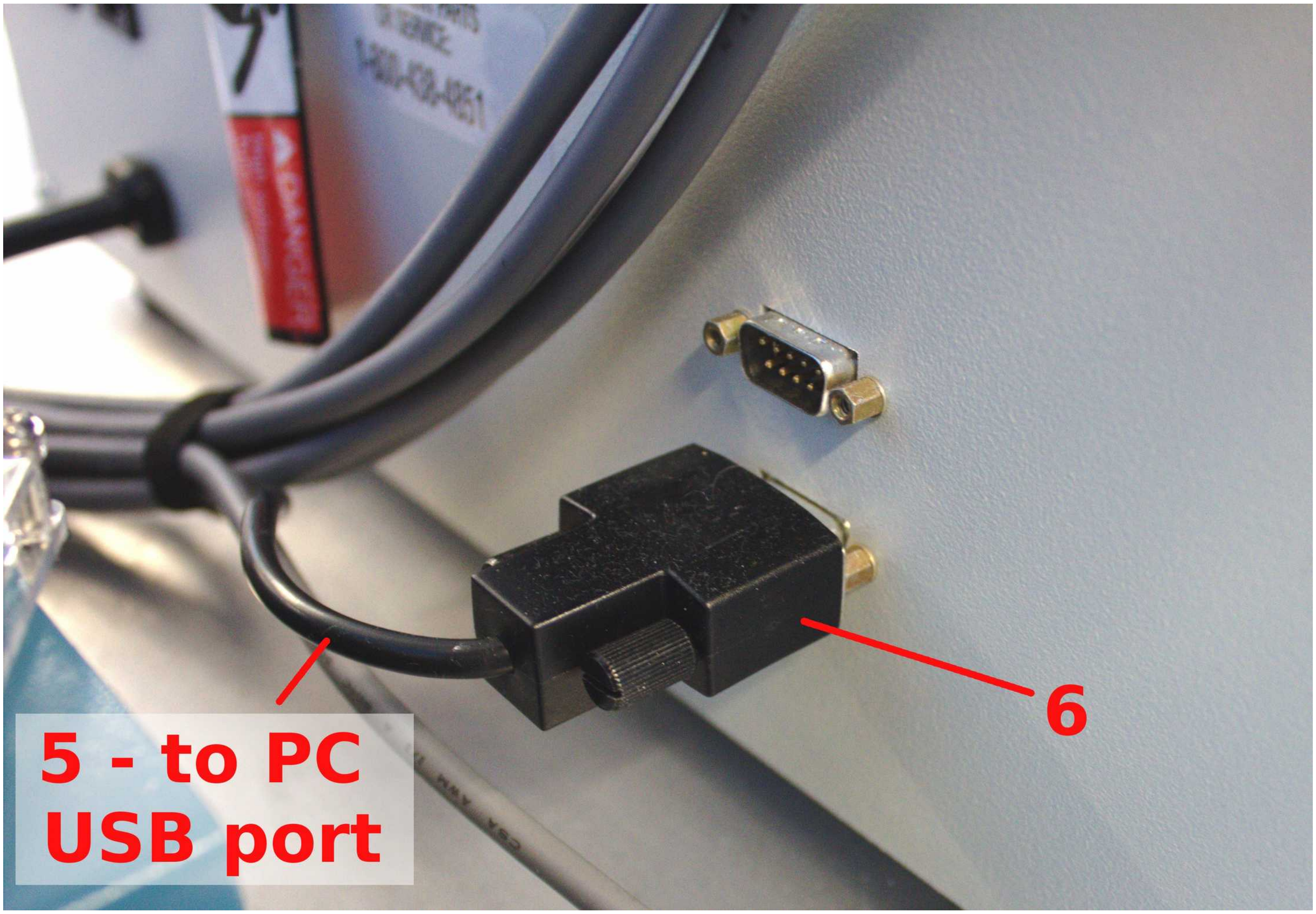

Comments:

- Pinout: Should be standard RS-485 pinout.

Subsystem 16: Cables – Butterfly valve

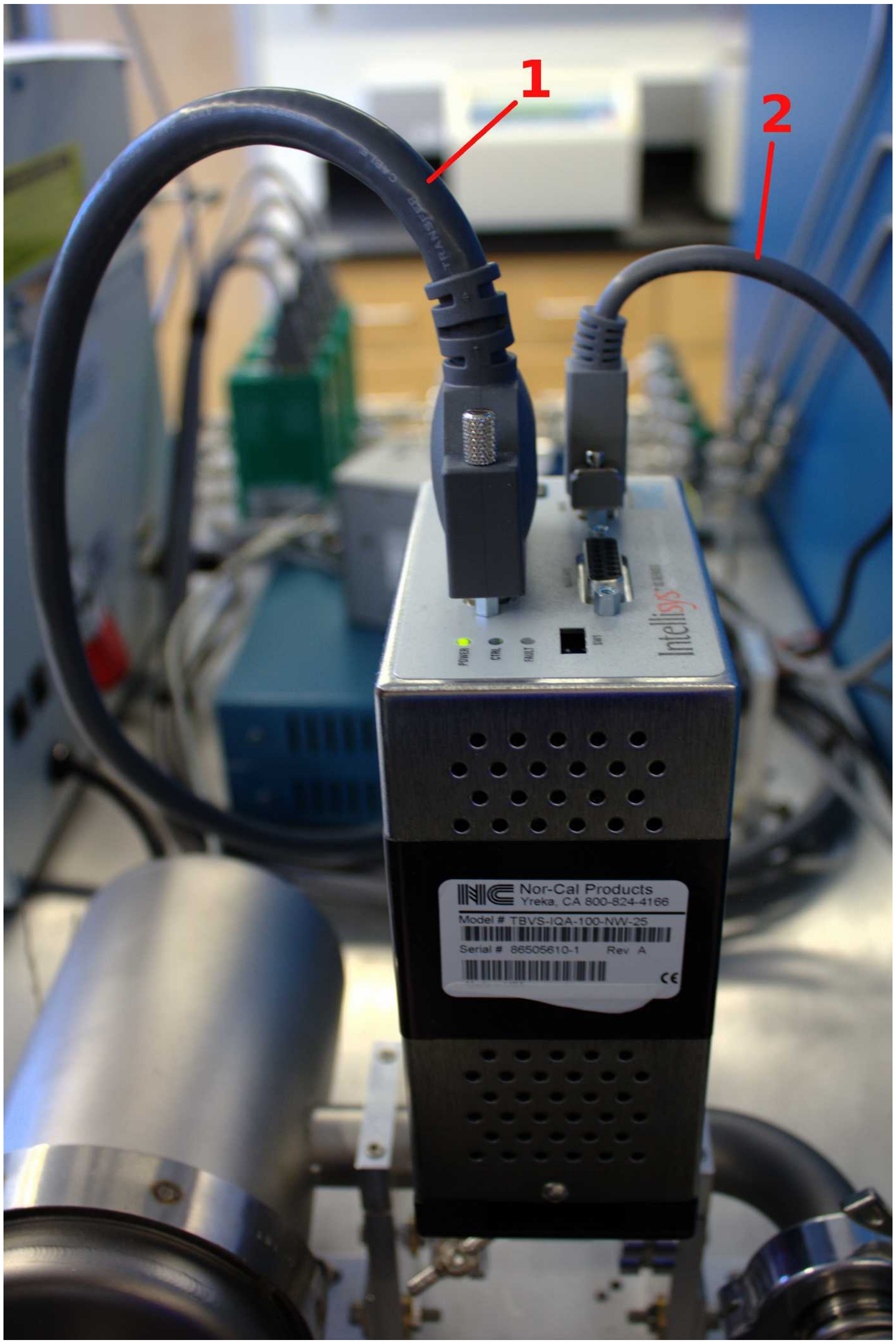

Comments:

- Pinout: *Appendix A: Butterfly valve cables*

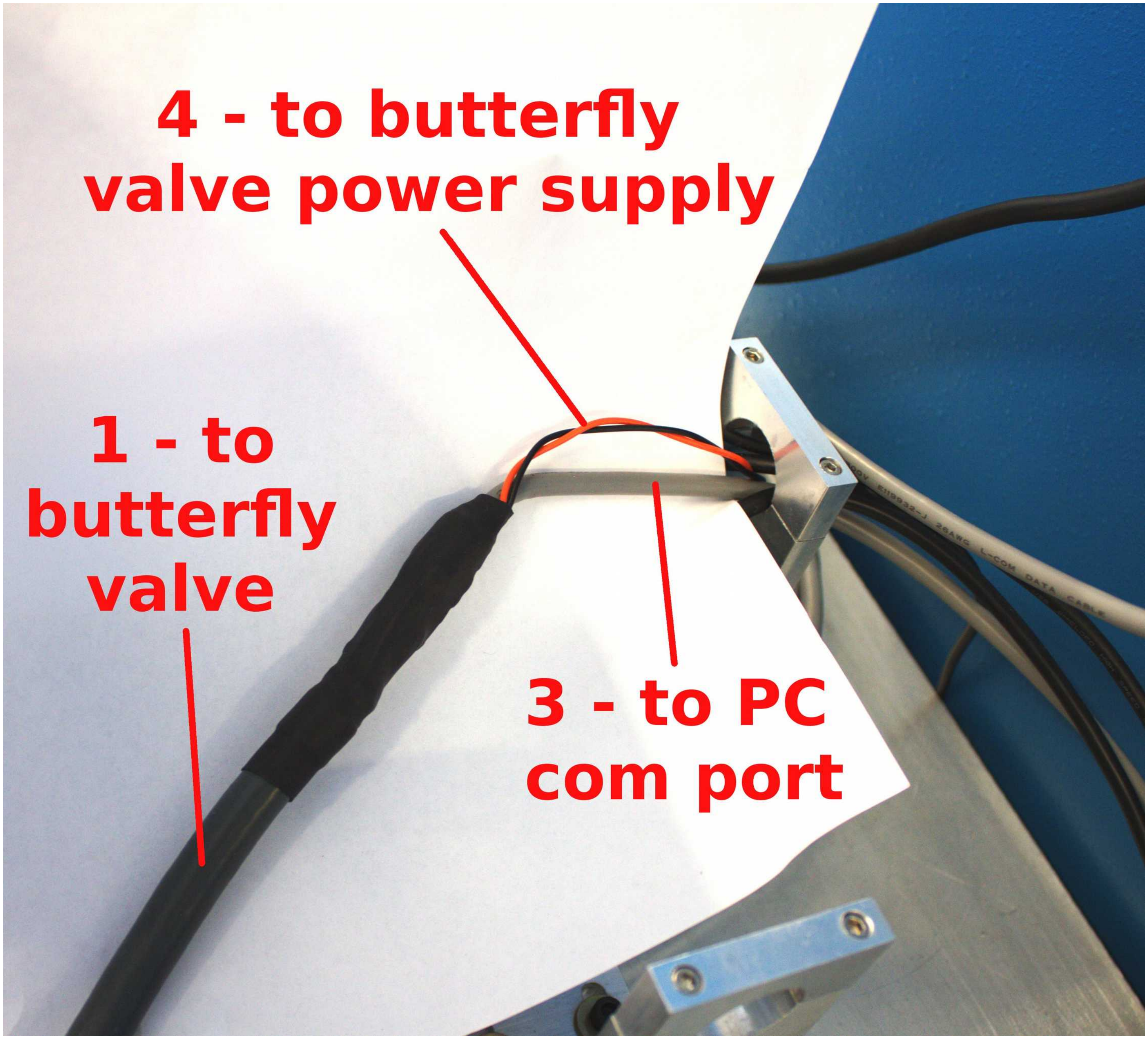
4 - to butterfly valve power supply
1 - to butterfly valve
3 - to PC com port

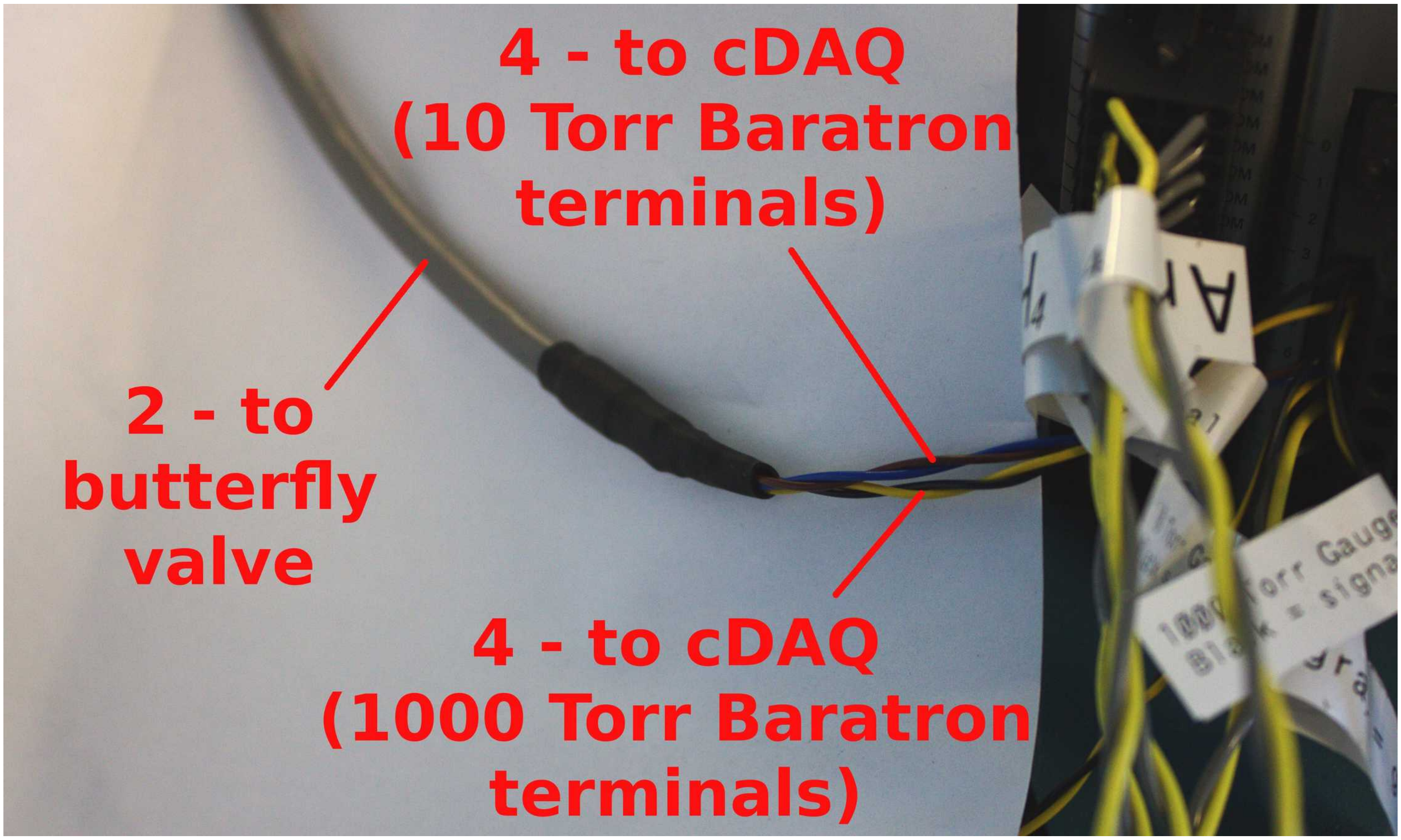
4 - to cDAQ
(10 Torr Baratron
terminals)
2 - to
butterfly
valve
4 - to cDAQ
(1000 Torr Baratron
terminals)

Subsystem 16: Cables – Cable management

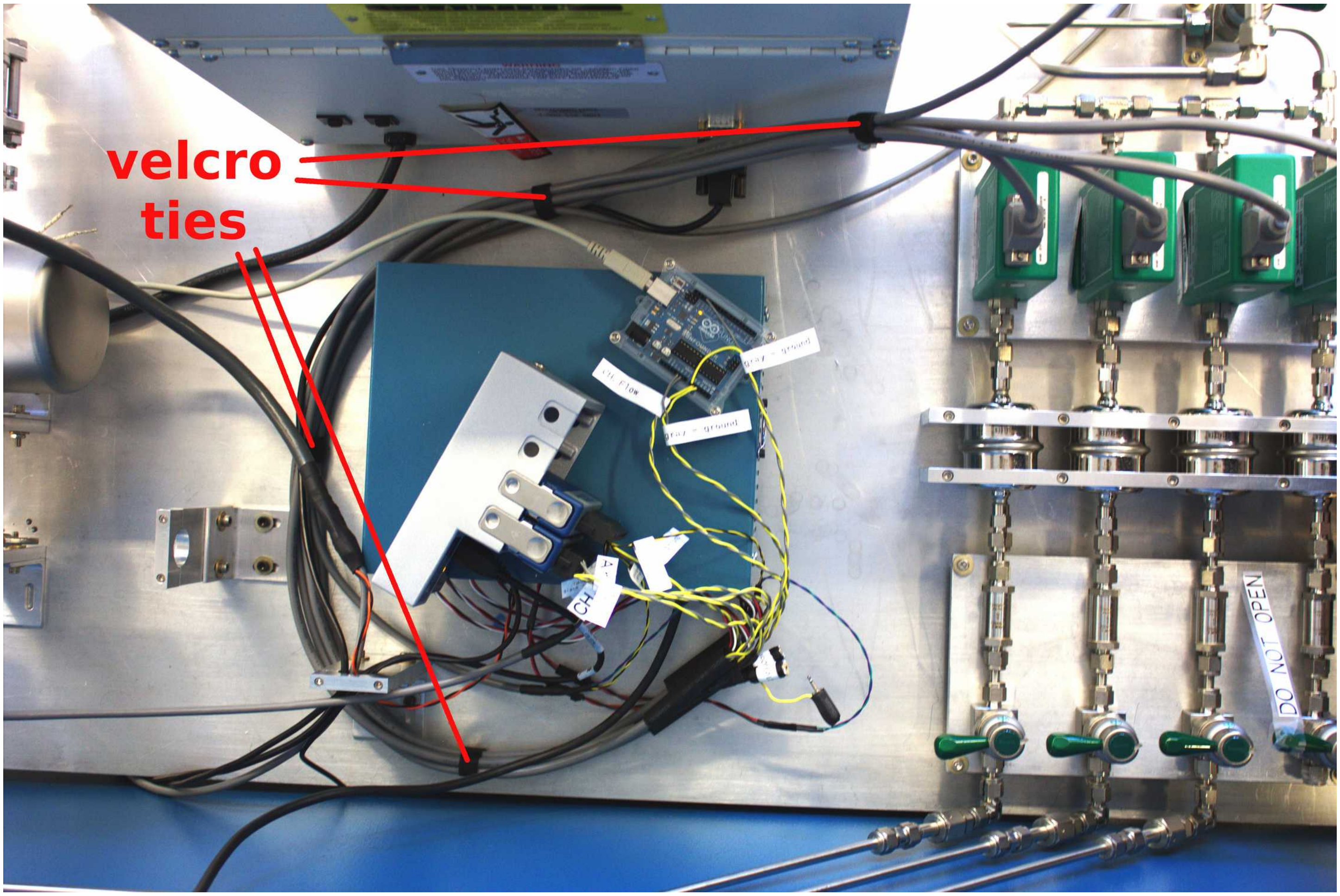

Comments:

- Cables given extra length for possible future modification of system.

Subsystem 17: Data acquisition & control

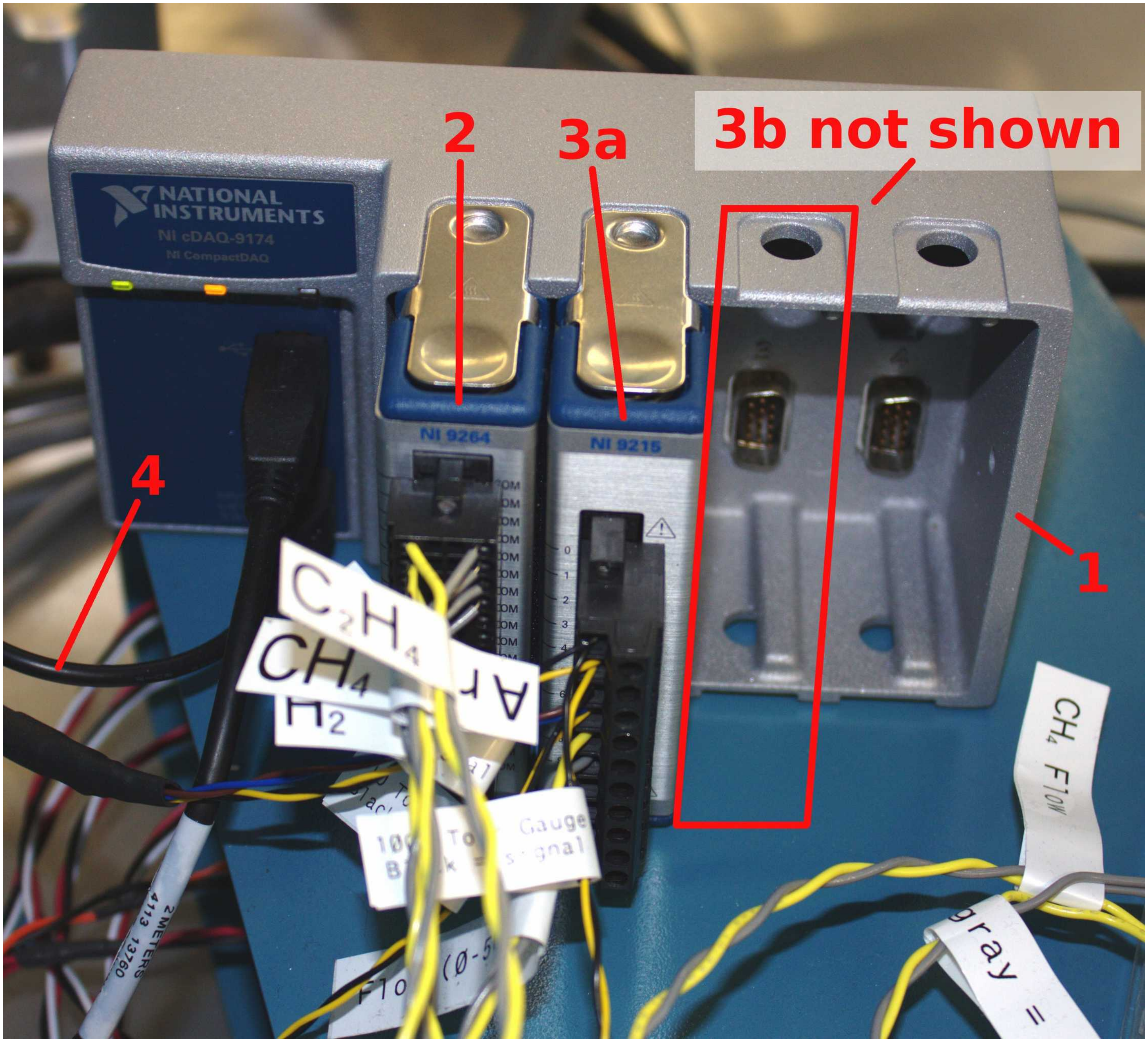

Comments:

- CVD shown uses an Arduino for 3 of the gas flow rate analog to digital conversions, but the Arduino's ADC must run outside of it's accepted voltage during MFC startup, so Arduino was not included in published design. Part 3b is an additional ADC to replace the Arduino.

Subsystem 18: Power supplies

Comments:

- Power supply used was a one-off made by our engineering staff. Consult your institution's engineering staff to discuss design and safety considerations.

Subsystem 19: Base and supports – Base plate

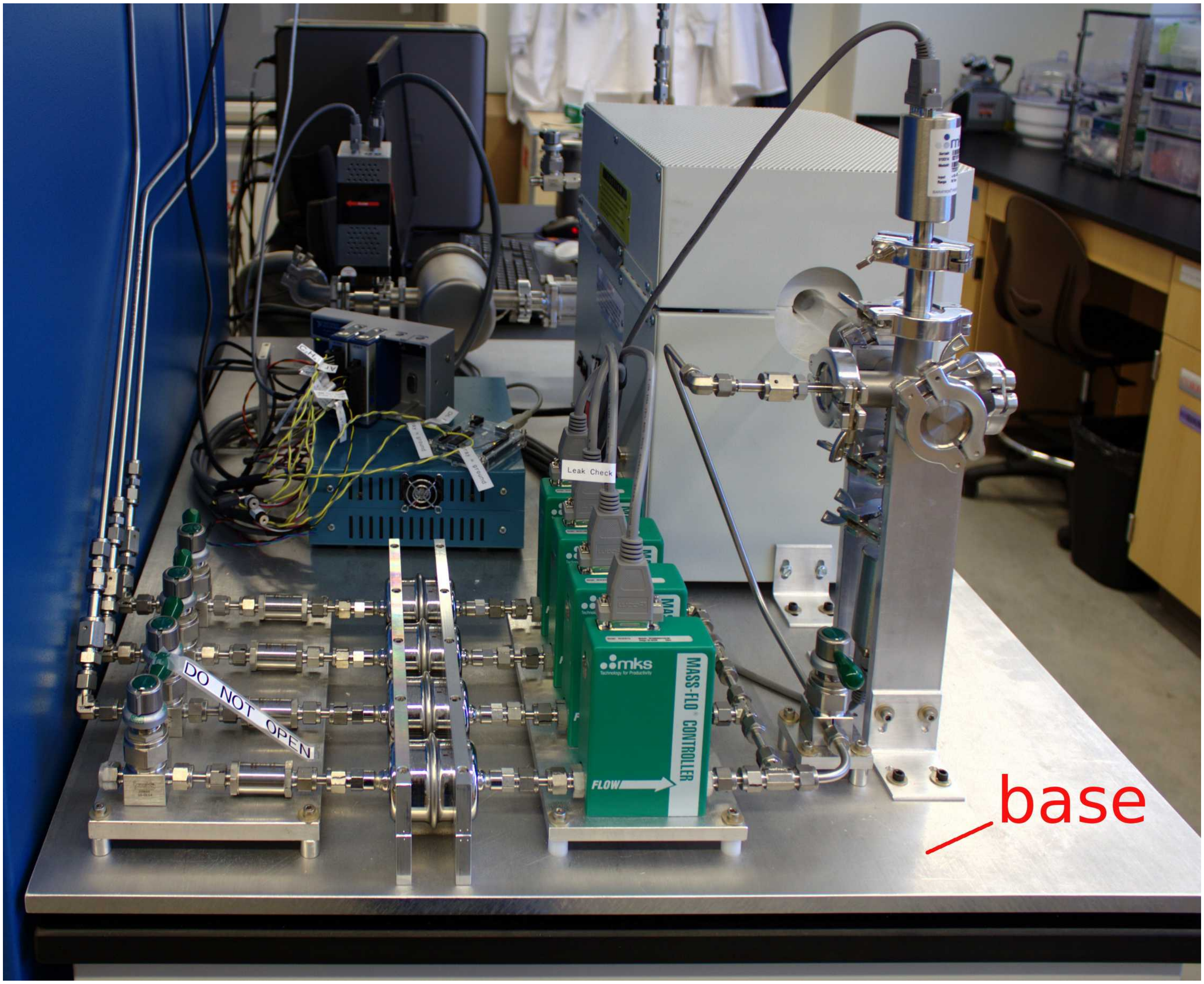

Comments:

- Rubber feet are attached to underside.

Subsystem 19: Base and supports – Gas filter lines

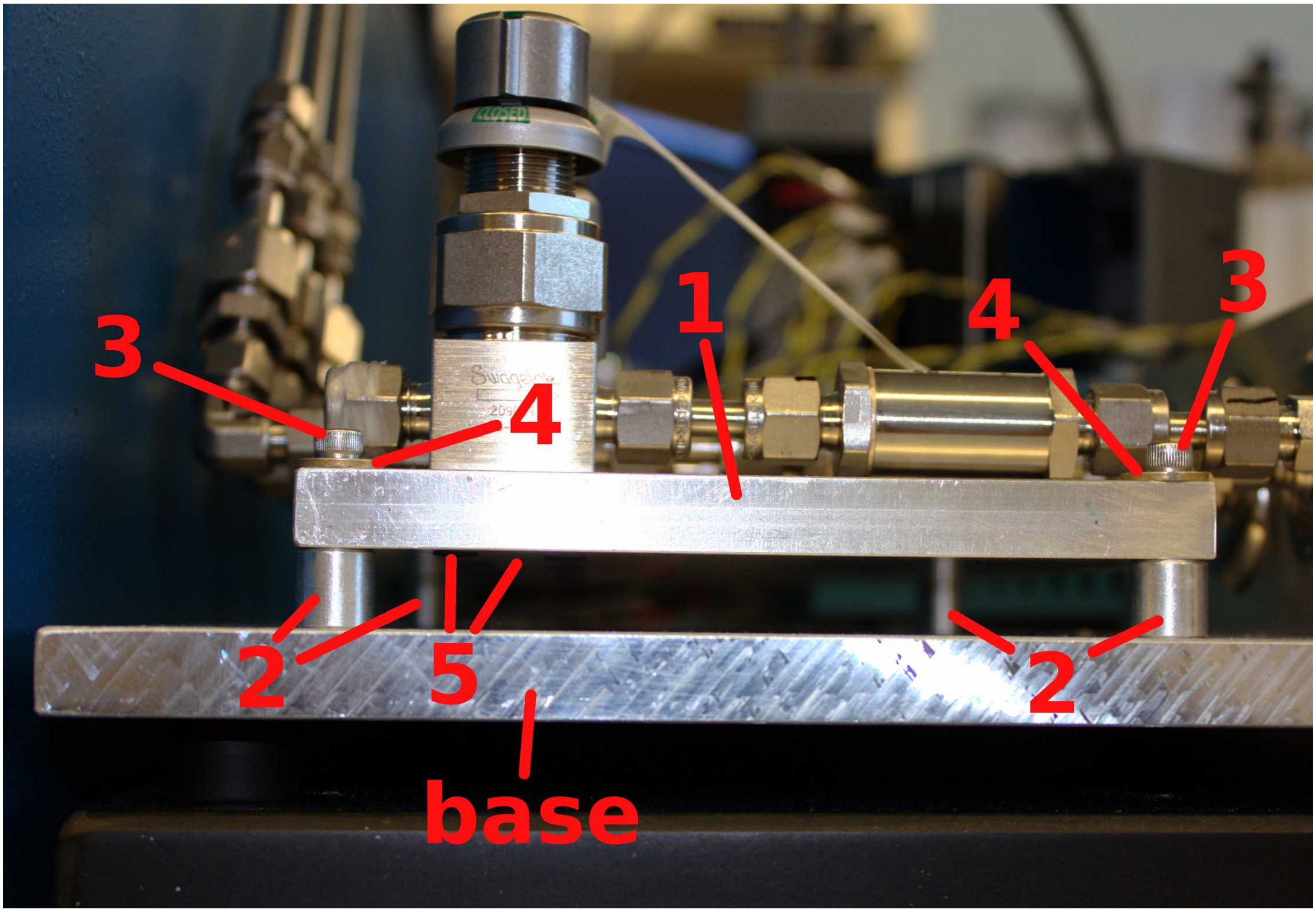

Comments:

- Use washers as spacers if mounting cap screws are too long.

Subsystem 19: Base and supports – Mass flow controllers

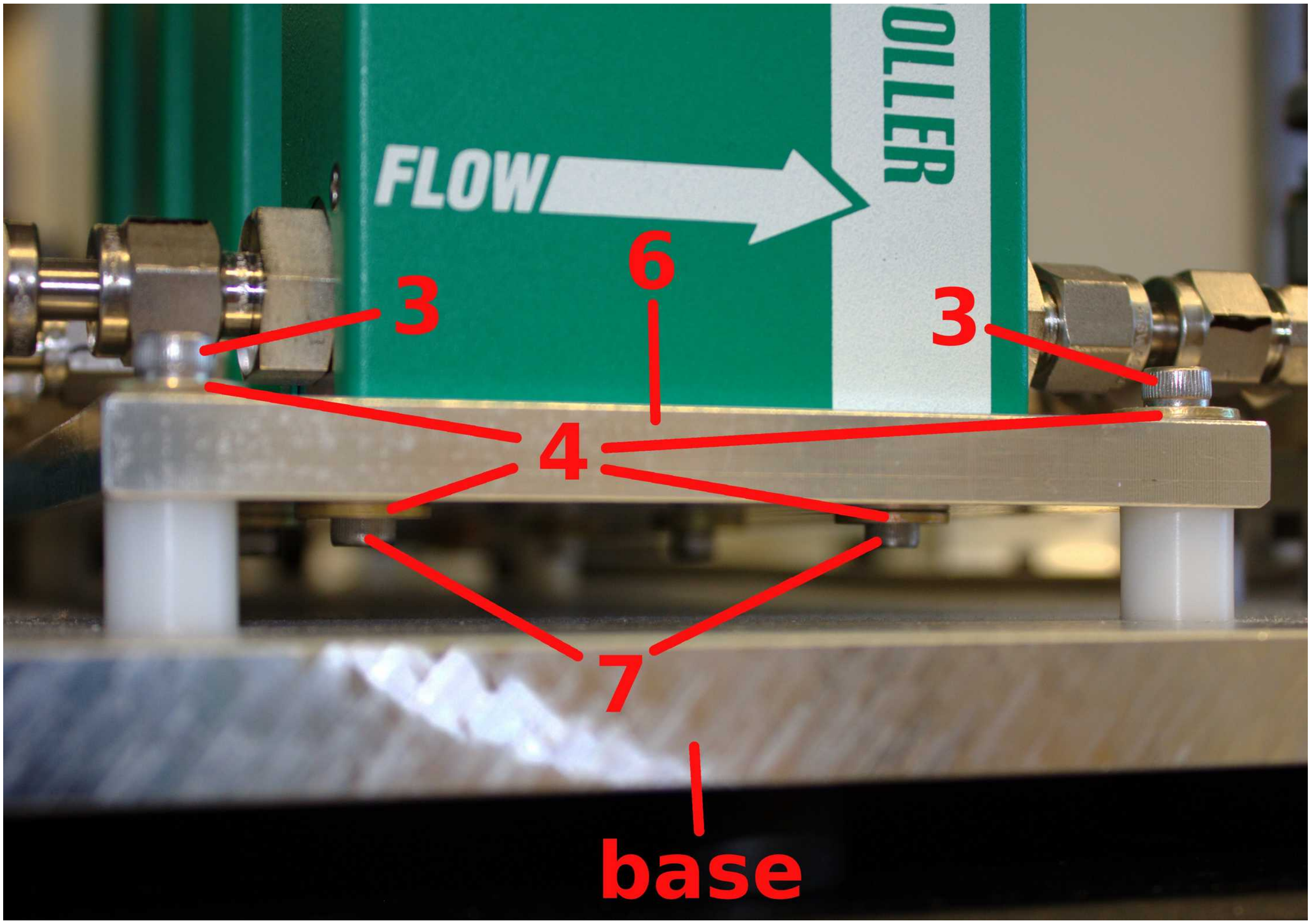

Comments:

- Use washers as spacers if mounting cap screws are too long.

Subsystem 19: Base and supports – Gas mixing line

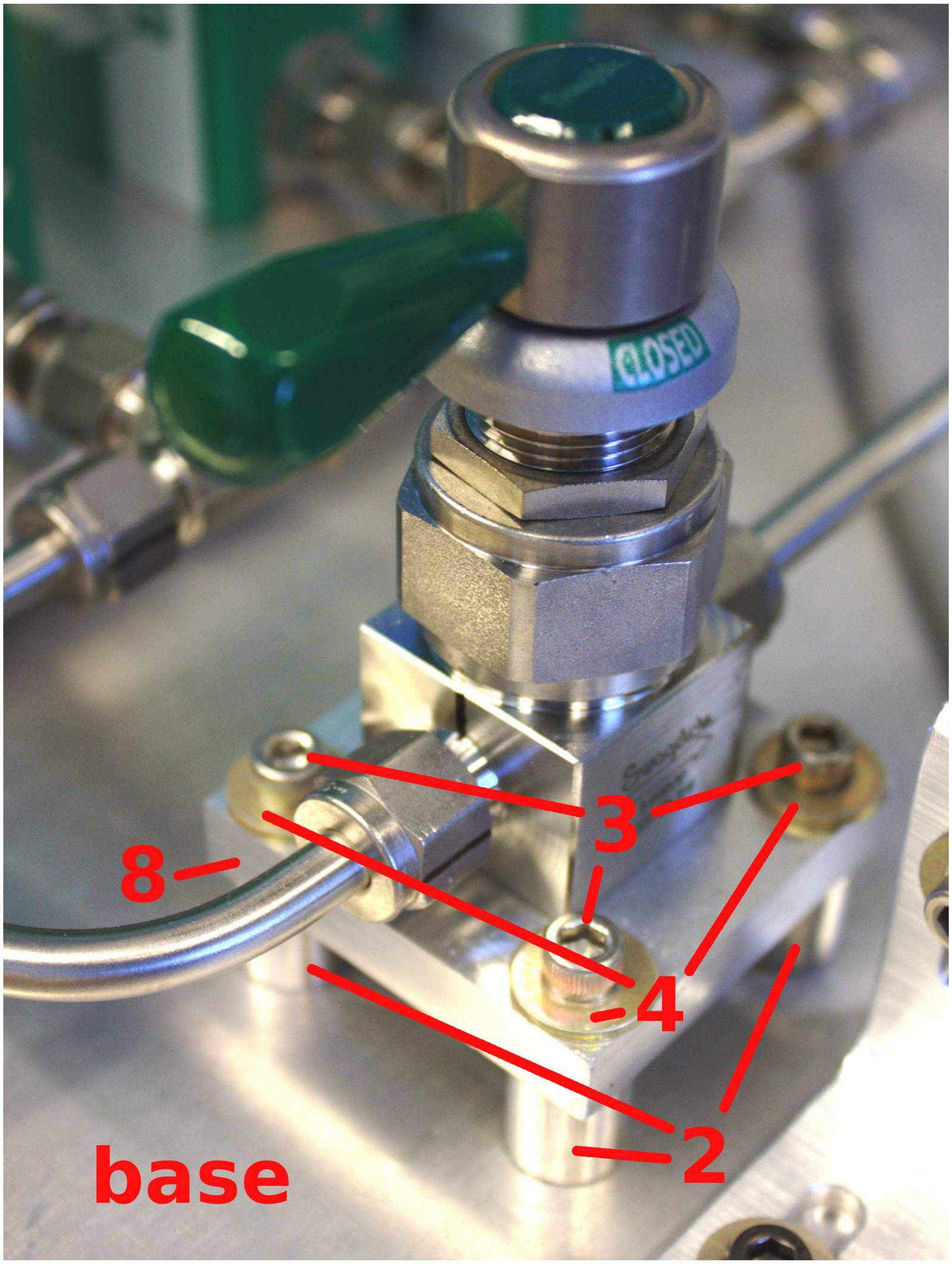

Comments:

- Use washers as spacers if mounting cap screws are too long.

Subsystem 19: Base and supports – Intake manifold

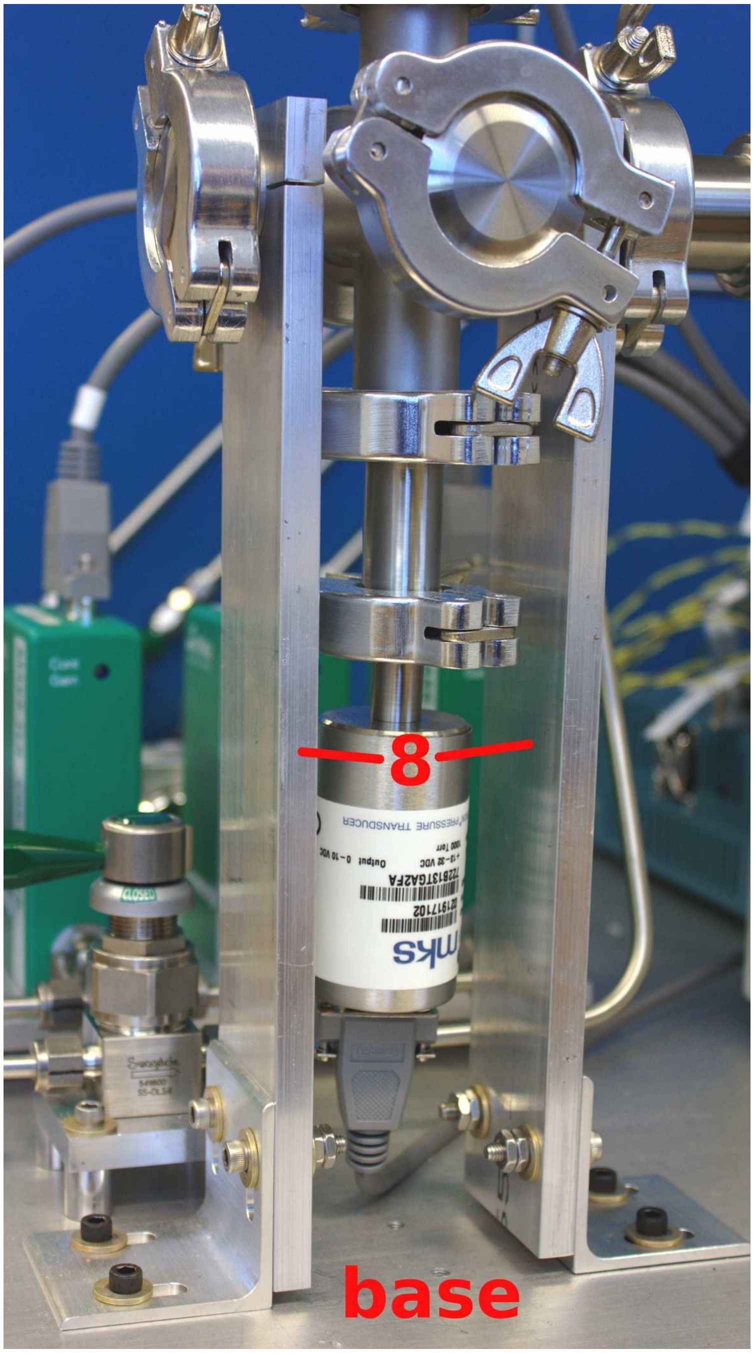

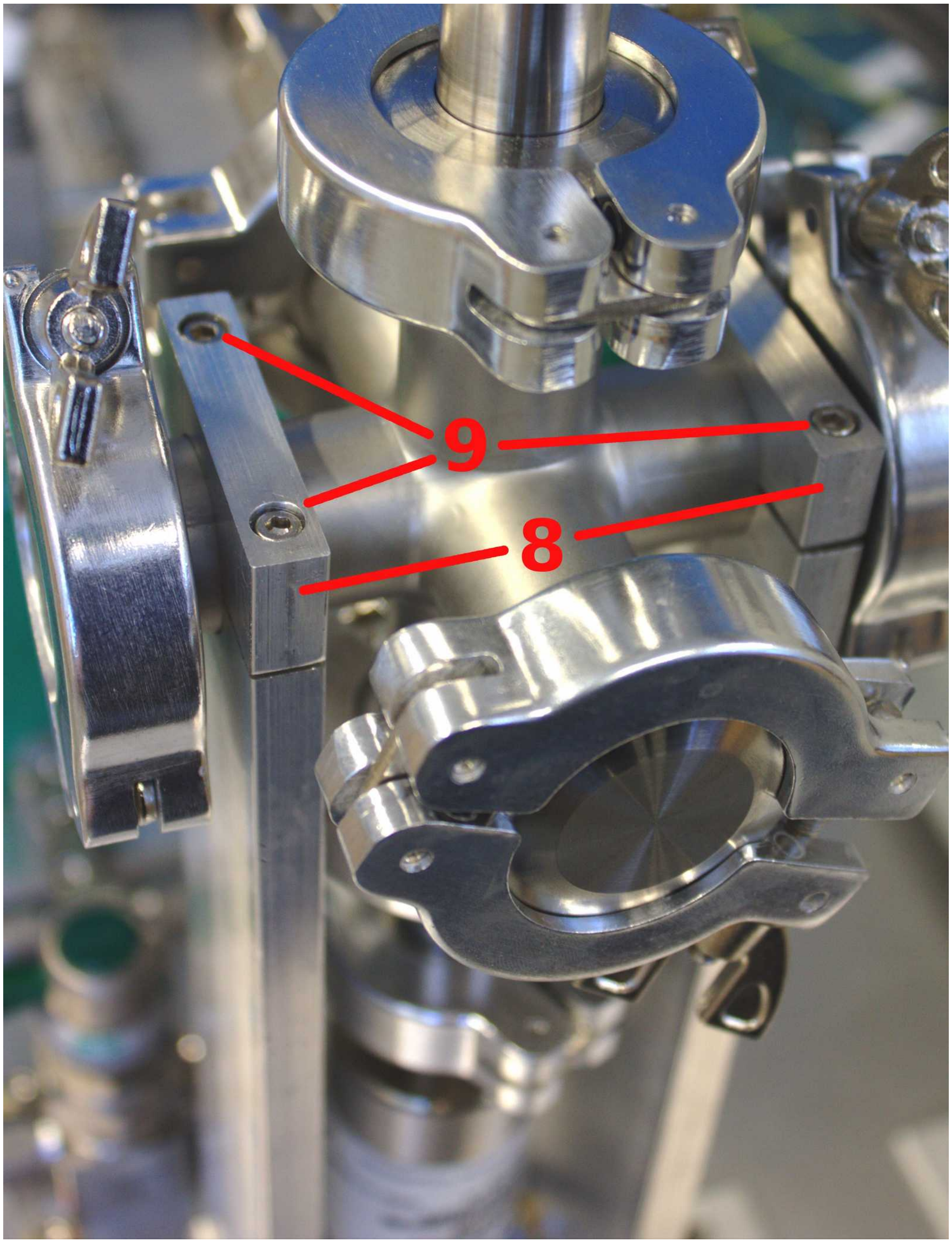
9
8

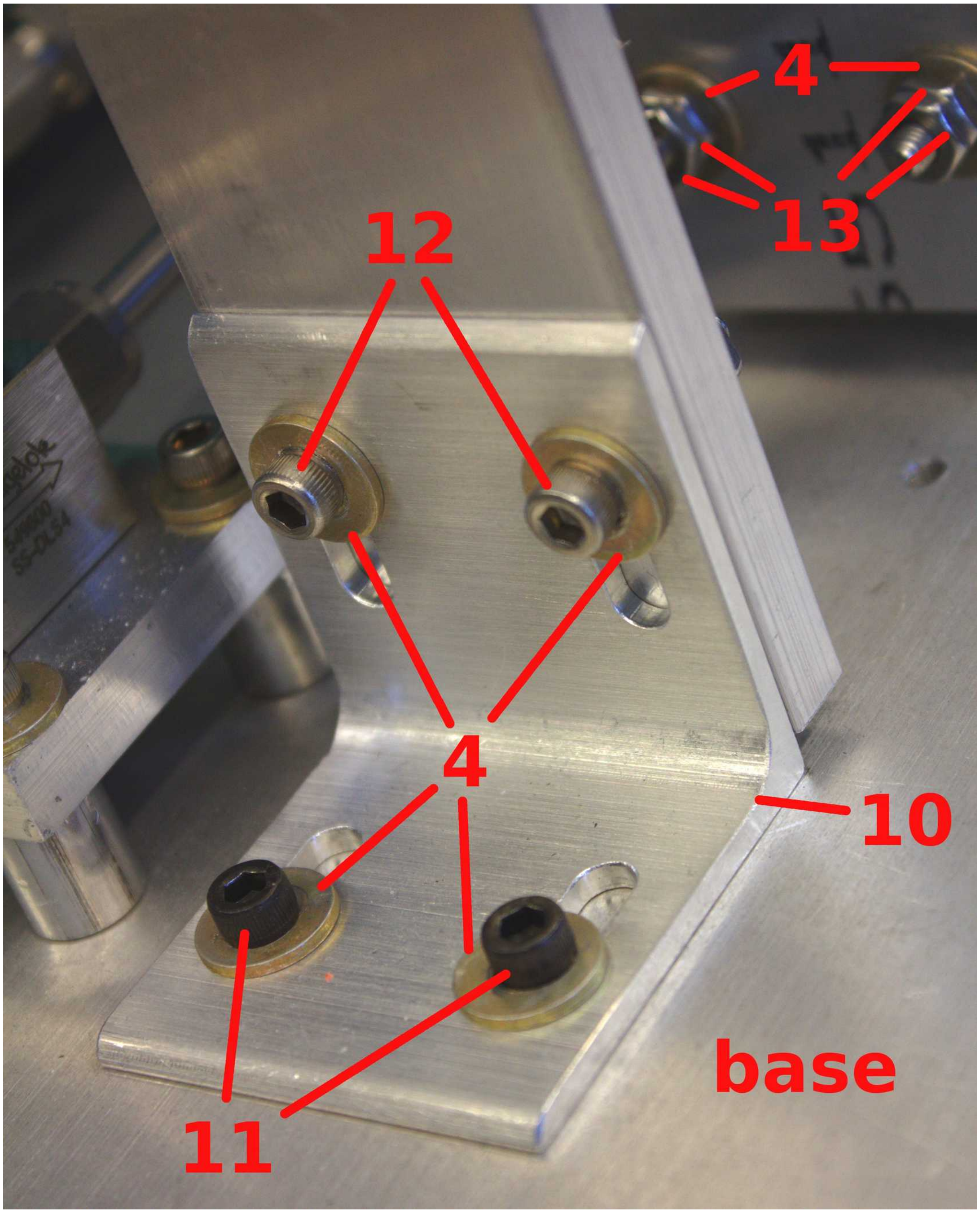

4
13
12
4
10
base
11

Subsystem 19: Base and supports – Furnace

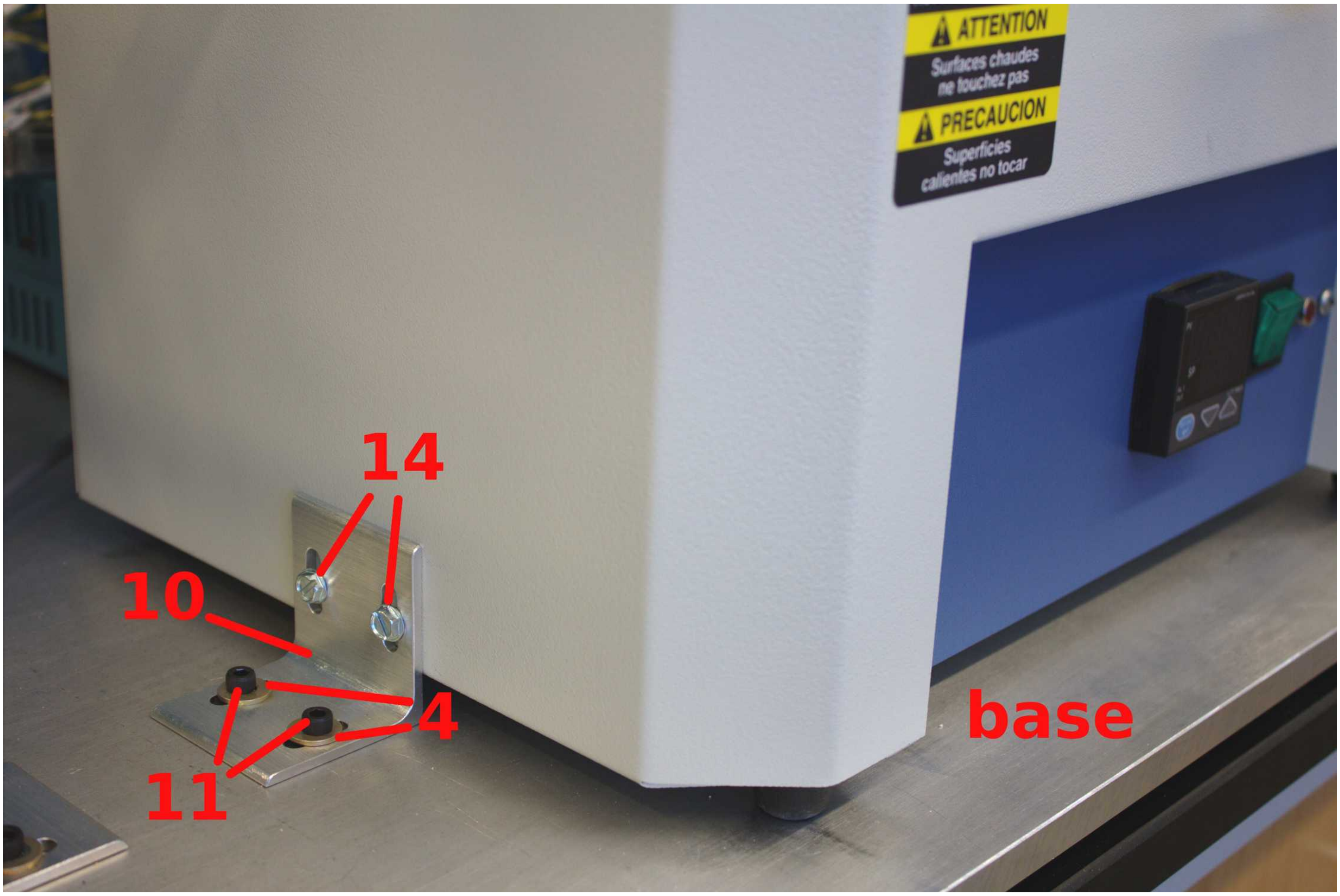

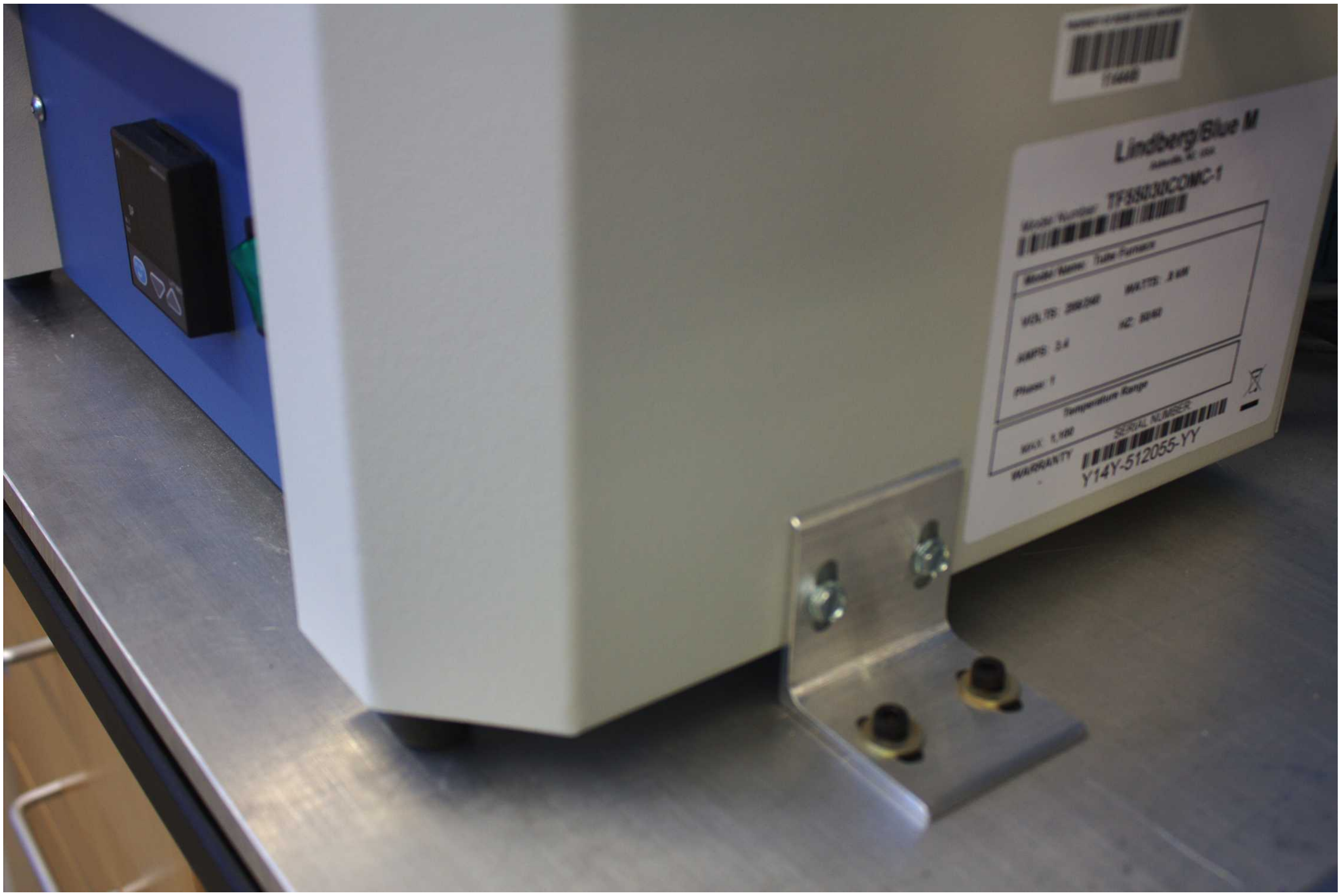
Lindberg/Blue M
TF55030COMC-1
SERIAL NUMBER
Y14Y-512055-YY
WARRANTY

Subsystem 19: Base and supports – Exhaust manifold

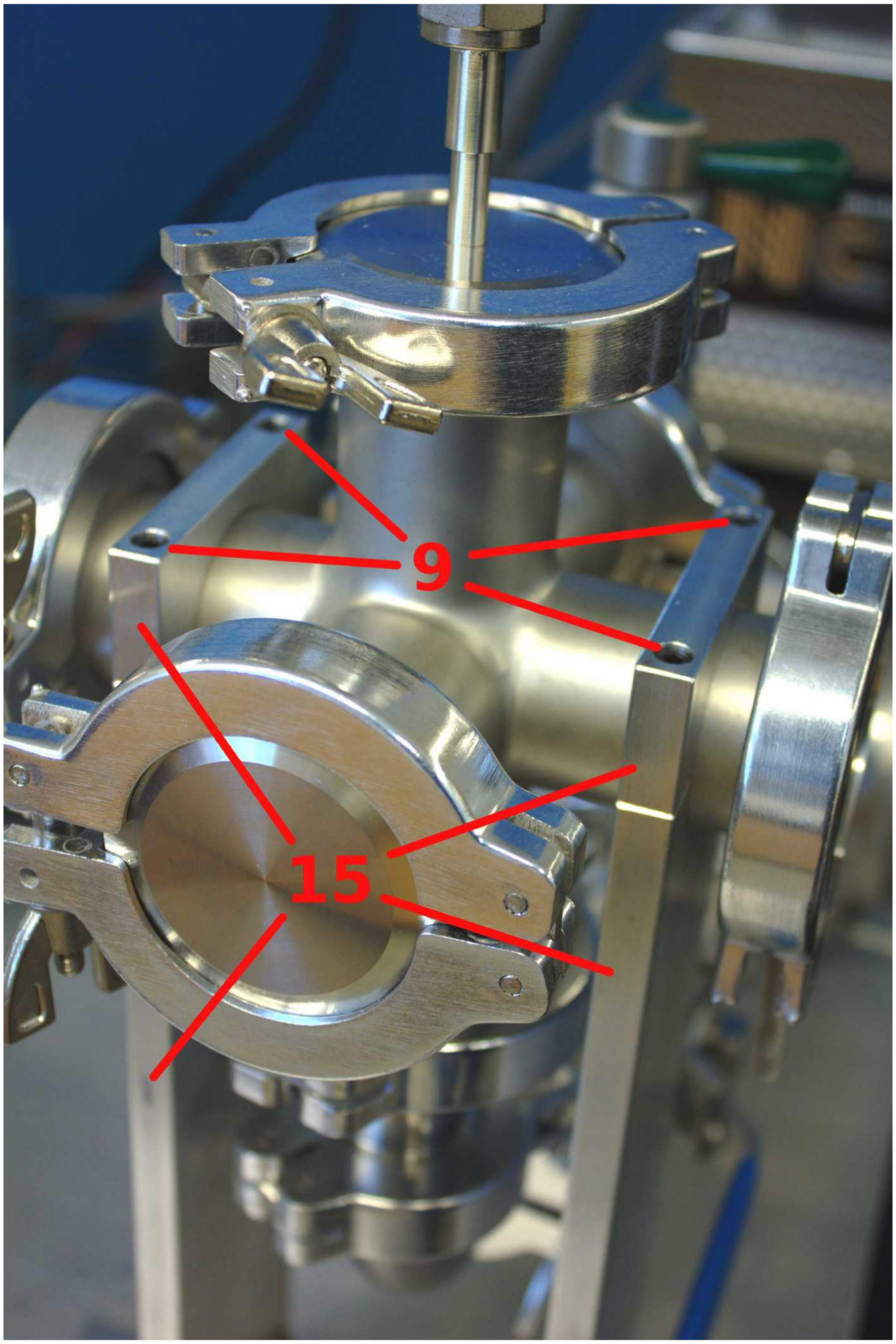

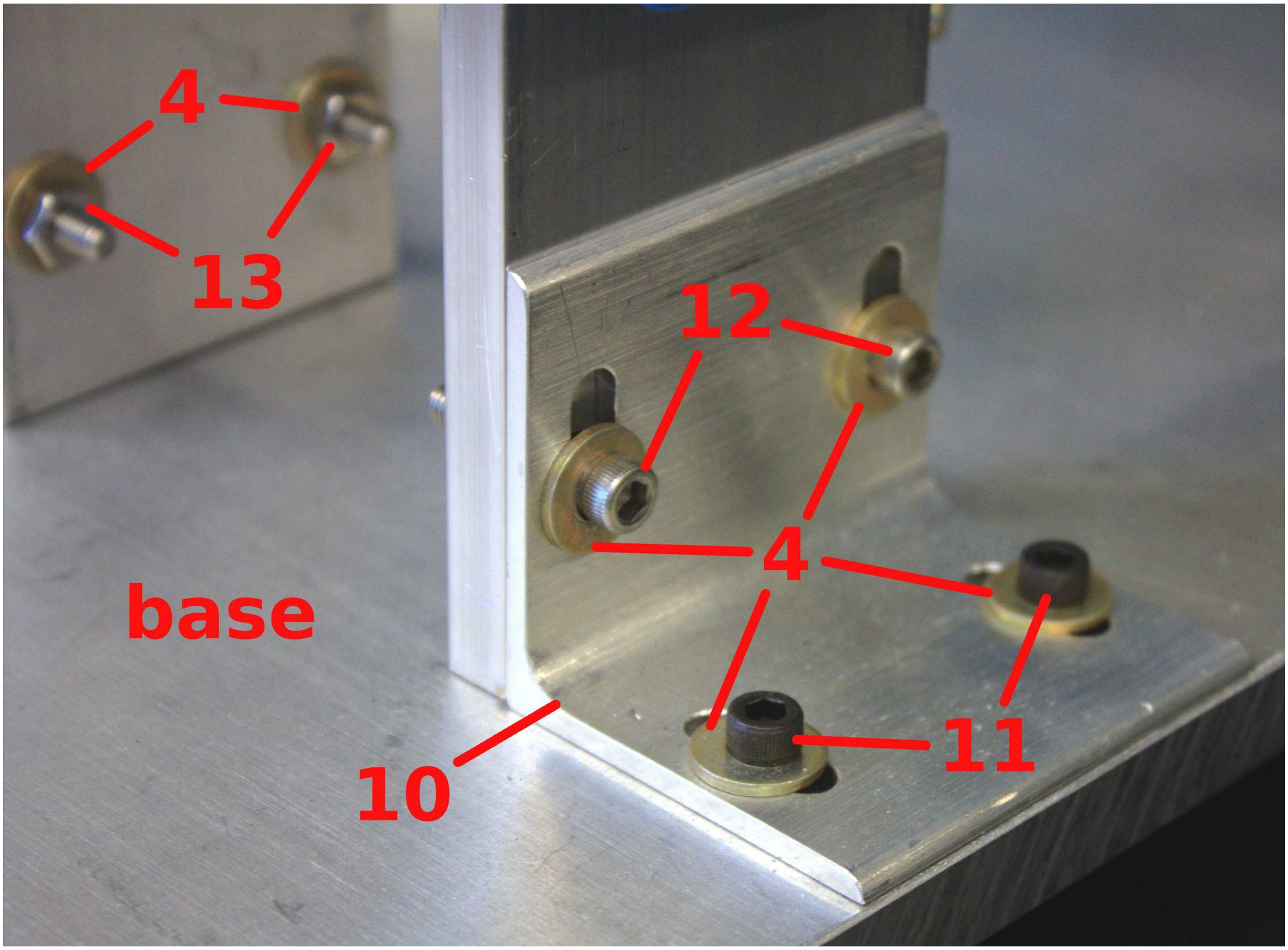

4
13
12
4
base
11
10

Subsystem 19: Base and supports – Exhaust line

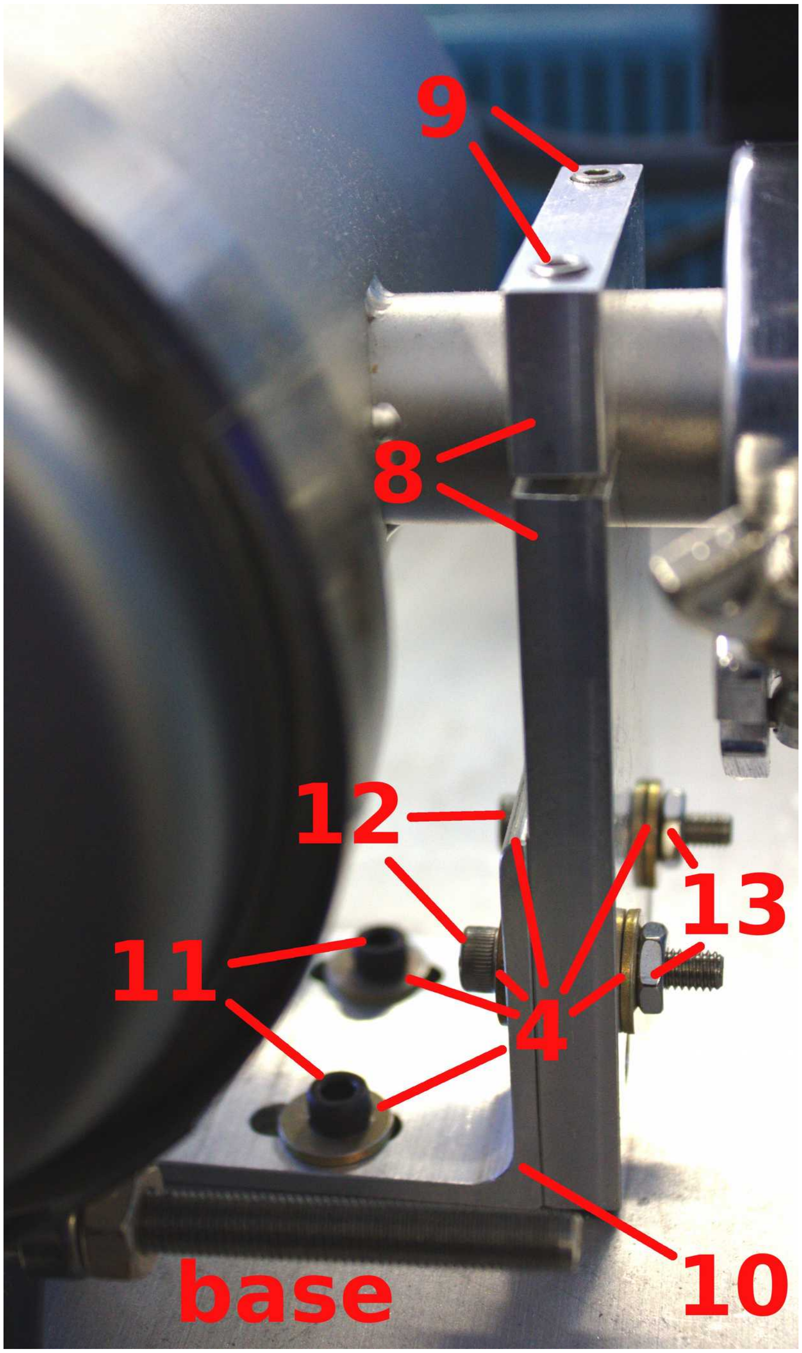

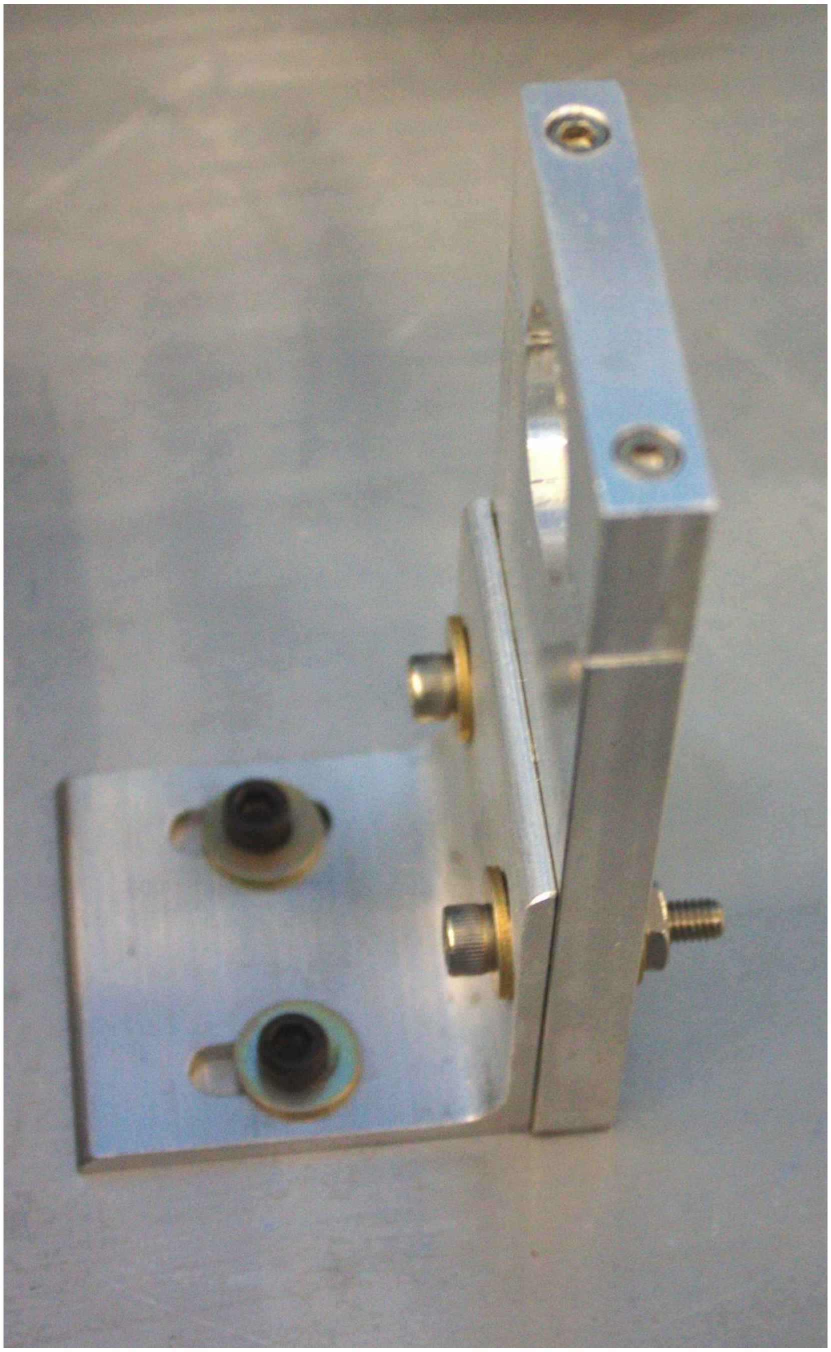

Appendix A: Cable pinouts

Note: Actual wire colors may be different from those shown. Verify pinout/color of cables before cable construction.

Also, the butterfly valve cable could be a DB-15 cable instead of a DB-25 cable. DB-25 cable was used on this system because we initially though we wanted control of the TTL pins.

Mass flow controller cables

pin	color(stripe)	name	function	
1	black	None		Not used
2	brown	Flow Output	Flow output signal (0-5VDC to signal common)	To ADC (flow reading signal)
3	red	Close Valve	Connect to signal common (pin 12) to close valve	Leak check cable only
4	orange	Open Valve	Connect to signal common (pin 12) to open valve	Leak check cable only
5	yellow	Power Ground	Power Ground	To power supply (ground)
6	green	-15VDC	-15VDC, 200mA	Not used
7	blue	+15VDC	+15VDC, 200mA	To power supply (15VDC, 200mA)
8	purple	Setpoint	Setpoint to signal common (0-5VDC = 0-100% of max range)	To DAC (flow setpoint signal)
9	gray	None		Not used
10	white	None		Not used
11	pink	Signal Common	Signal Common (for signal in, out, open, close)	To DAC (flow setpoint ground)
12	light blue	Signal Common	Signal Common (for signal in, out, open, close)	To ADC, (flow reading ground)
13	black(white)	None		Not used
14	brown(white)	None		Not used
15	red(white)	Chassis Ground	Chassis Ground	Not used

Pressure gauge cables

pin	color(stripe)	name	function	
1	black	Pressure out	0 – 10 VDC = 0 – 100% of full range	To ADC (signal)
2	gray	None		
3	red	None		
4	green	+ 15VDC	15 VDC, 10 mA max	To power supply (15 VDC, 10 mA)
5	brown	None		
6	blue	None		
7	orange	None		
8	yellow	Pressure gnd	Pressure signal ground	To ADC (ground)
9	purple	Power gnd	Power ground	To power supply (ground)

Butterfly valve cables

Butterfly valve main cable Connects to DB25 Receptacle on butterfly valve, to RS232 cable, to 24VDC power

pin	color (stripe)	name	function	
1	black	Chassis GND	This pin is directly connected to the controller chassis as well as to the A/C power Ground pin. Chassis Ground is decoupled from digital ground by a 100kΩ resistor and a 0.1μF capacitor in parallel with two 22μF capacitors in series.	Not used
2	brown	RS-232TX (data from IQ to host)	Connect to this pin for data transmitted from the APC controller to the host computer, whenever RS-232 communications are used.	To RS232 cable, pin 2
3	red	RS-232RX (data from host to IQ)	Used for data transmitted from the host to the APC controller, whenever RS-232 communications are used.	To RS232 cable, pin 3
4	orange	Analog CDG (Capacitance Diaphragm Gauge) Out	The signal from the CDG input can be monitored on this pin. It should be referenced to Analog Ground. The Analog CDG output is not a direct pass through of the pressure signal; rather it is a digitized output with a finite resolution of about 5mV with an accuracy of 10mV with respect to the input signal. At crossover from one gauge to the next, this output changes to track the active gauge's signal.	Not used
5	yellow	Analog GND	Analog Ground should be used as a reference to all analog output signals (i.e. CDG1, CDG2, Valve Position). It should NOT be used as the reference for the analog set-point input, nor should it be used for the power common connection. Analog Ground and Digital Ground are directly tied together.	Not used
6	green	Analog Valve Out	This signal should be referenced to the Analog Ground. This digitized output signal is normally a 0 to 10 VDC output, where 0 represents a fully closed valve and 10 represents a fully open valve. It has a finite resolution of about 5 mV with an accuracy of 25mV.	Not used
7	blue	Digital GND	This pin should be used as the reference to TTL input signals (i.e. \Analog SP Control Select\, \CDG2 Select\, \Position Control Select\, etc..) DO NOT use this pin as reference to the TTL output signals.	To RS232 cable, pin 5
8	purple	\Analog SP Control Select In\	Analog Set Point Control Select is an ACTIVE LOW TTL input that should be referenced to Digital Ground. This input must be pulled low whenever analog pressure- or position control mode is desired. This does not, however, disable the RS-232 serial port. In other words, a serial command or inquiry will be acted upon irrespective of the status of this input. If a serial command is issued such that the operating state or condition of the APC controller is changed, then the Analog Set Point Control Select pin must be toggled for a minimum of 200 msec in order to switch the controller back to analog control mode.	Not used
9	gray	Analog SP+ In	This is the positive terminal of the analog set point differential voltage input. When referenced to the negative terminal (Analog Set Point (-) Input), the differential voltage input should always be in the range from 0V to 10V. The voltage input across these terminals determines the analog set point value to which the APC controller will control, either in pressure or position mode.	Not used
10	white	Analog SP- In	This is the negative terminal of the analog set point differential voltage input. Use this as a reference to Analog Set Point (+)Input.	Not used
11	pink	Power RTN	This is the negative terminal of the device power differential voltage input on DC powered controllers. Use this as a reference to the positive +24V DC power input.	To power supply (ground)

12	light blue	Power +24V In	This is the positive terminal of the device power differential voltage input on DC powered controllers. When referenced to the negative terminal (Power RTN input), the differential voltage input should always be in the range of 24V ± 10%. The voltage input across these terminals provide the operating power for the device.	To power supply (24 VDC, 4A)
13	black (white)	\CDG2 Select In\	CDG2 Select is an ACTIVE LOW TTL input that should be referenced to the Digital Ground. This input must be pulled low whenever pressure control using CDG2 is desired. If left high, then pressure control will, by default, always be performed using the input from CDG1.	Not used
14	brown (white)	\Hold Valve Select In\	This function is an ACTIVE LOW TTL input that should be referenced to Digital Ground. The valve will stop in its present position when this pin is pulled low. This is for butterfly valves.	Not used
15	red (white)	\Position Control Select In\	Position Control Select is an ACTIVE LOW TTL input that should be referenced to Digital Ground. Pulling this pin low will cause the APC controller to use the analog set-point value as a position command, provided that \Analog Set Point Control Select\ is also pulled low. Leaving it high will by default cause the controller to perform pressure control.	Not used
16	orange (white)	No Connection		Not used
17	green (white)	Analog GND		Not used
18	blue (white)	\Close Valve In\	This function is an ACTIVE LOW TTL input that should be referenced to Digital Ground. The valve will close when this pin is pulled low. However, the pin must be kept low during the entire valve stroke. If the pin is allowed to go high mid-stroke of the valve, then the valve will stop in that position.	Not used
19	purple (white)	\Open Valve In\	This function is an ACTIVE LOW TTL input that should be referenced to Digital Ground. The valve will open when this pin is pulled low. However, the pin must be kept low during the entire valve stroke. If the pin is allowed to go high mid-stroke of the valve, then the valve will stop in that position.	Not used
20	red (black)	Fault Out	Fault Status Output is an ACTIVE HIGH TTL output that should be referenced to the TTL Output Common pin. If the controller is powered off, or if a FAULT condition is present, then this pin will be high. When the controller is operating normally, this pin will be low.	Not used
21	orange (black)	No Connection		Not used
22	yellow (black)	\Valve Open Out\	Valve Open Output is an ACTIVE LOW TTL output that should be referenced to TTL Output Common pin. This pin will be low only when the valve is open.	Not used
23	green (black)	\Valve Closed Out\	Valve Closed Output is an ACTIVE LOW TTL output that should be referenced to TTL Output Common pin. This pin will be low only when the valve is closed.	Not used
24	gray (black)	TTL Out Common	This pin should be used as a reference to all TTL outputs (i.e. Fault, Valve Open and Valve Closed).	Not used
25	pink (black)	Chassis GND		Not used

	**RS-232 Cable**		Goes from butterfly valve main cable to PC
1	Black		
2	Gray	Receive Data (into PC)	To butterfly valve main cable, pin 2
3	Red	Transmit Data (out of PC)	To butterfly valve main cable, pin 3
4	Green		
5	Brown	Signal Ground	To butterfly valve main cable, pin 7
6	Blue		
7	Orange		
8	Yellow		
9	Purple		

	**Gauge Connector Cable**		Goes from DB9 receptacle to pressure gauge pins on cDAQ ADC	
1	Black	Signal input from gauge 1	From 1000 Torr	To ADC (1000 Torr signal pin)
2	Gray	+15 V supply to gauge	Power (not used in this design)	Not used
3	Red	- 15 V supply to gauge	Power (not used in this design)	Not used
4	Green	Not used		Not used
5	Brown	Signal input from gauge 2	From 10 Torr	To ADC (10 Torr signal pin)
6	Blue	Signal common from gauge 2	From 10 Torr	To ADC (10 Torr ground pin)
7	Orange	No Connection		Not used
8	Yellow	Signal common from gauge 1	From 1000 Torr	To ADC (1000 Torr ground pin)
9	Purple	Power supply common	Power (not used in this design)	Not used

Appendix B: Control Program Setup

Butterfly valve sub-VIs:

sub-InitializeValve.vi

Choose correct com port, number of gauges, and full ranges of gauges. On running the VI, the reply for gauge 1 should be: “N1” followed immediately by the full range. For gauge 2 it will be “N2” followed by the full range.

For 1000 Torr and 10 Torr, the replies will be: “N11000.000” and “N210.000”. Mark down the butterfly valve's com port for future reference.

If this works, both sub-StartValveSerial.vi and sub-SendReceiveValve.vi are also working.

sub-OpenValve.vi, sub-CloseValve.vi

Use the butterfly valve com port found above. The valve movement should be seen at the base of the valve motor. Also, the open and closed lights at the top of the valve motor should change in response to running the open valve and close valve programs.

sub-ReadValveSetpoint.vi

Use the butterfly valve com port found above. The valve reply should be “S1+” followed by the pressure setpoint given as a percent of the full range of gauge 1.

sub-SetValvePressure.vi

Using butterfly valve com port found above, and the correct gauge settings, and the desired pressure, the valve reply should be “S1+” followed by the pressure setpoint given as a percent of the full range of gauge 1.

sub-ReadValvePosition.vi

Use the butterfly valve com port found above. The reply will be “V+” followed by the open state of the valve as a percent.

Furnace sub-VIs:

sub-ReadTemperature.vi

Use the VISA resource name to find the correct com port for the furnace. If using a RS-485 to USB cable, the driver must be installed. When the correct com port is used and the driver is

installed, the thermocouple temperature shown when running the program will match the value on the front of the furnace.

Mark down the furnace's com port for future reference.

sub-ReadTempSetpoint.vi

Using the furnace com port found above, running this program should return the furnace's current setpoint, as shown on the front of the furnace.

sub-ChangeSetpoint.vi

Using the furnace com port found above, running this program should update the furnace's setpoint, as shown in green on the front of the furnace.

Mass flow controller sub-VIs:

sub-CheckArFlowRate.vi

This VI will have to be set to read the ADC channel on your cDAQ which corresponds to the flow rate signal from the Ar MFC. If no flow rate has been set, and the MFC is powered, the response should be a value close to zero. This VI can be run continuously to monitor the Ar flow rate.

sub-CheckCH4FlowRate.vi, sub-CheckH2FlowRate.vi, sub-CheckC2H4FlowRate.vi

If using a second cDAQ ADC module, copy sub-CheckArFlowRate.vi and replace the VIs for each of the other gases, modifying the settings of the DAQ assistant in the block diagram of each to correspond to the channel of the ADC used for the gas.

If using an Arduino, install the code below on an Arduino Uno, then plug the flow out from the MFCs to the analog ports given in the program. Then run sub-Arduino.vi. Once correct com port is chosen, it will show a string of 4 numbers when are then scaled for each of the gases.

If using an Arduino, sub-CheckCH4FlowRate.vi, sub-CheckH2FlowRate.vi, and sub-CheckC2H4FlowRate.vi can be used as is.

Note: The output of the MFC flow wires is outside of the stated range of an Arduino's ADC. This could potentially destroy your Arduino and possibly even damage your MFCs. That said, we have used this setup without problem.

sub-SetFlowRate.vi

In the block diagram, set the values in the case structure on the very left to the analog output channels for each MFC. Then set Ar to 10% flow and see if the argon flow rate changes (using sub-CheckArFlowRate.vi). Set Ar back to 0%, then repeat for the other gases.

Pressure gauge sub-VIs:

sub-Baratron1000Torr.vi

Modify the DAQ Assistant in the block diagram to the 1000 Torr Baratron's ADC channel. Running the program will return the pressure in Torr. Room pressure will be about 700 Torr, depending on your altitude. Program can be run continuously for pressure monitoring.

sub-Baratron10Torr.vi

Do the same as above for the 10 Torr Baratron. At room pressure, the pressure will read a little over 10 Torr (the limit of the pressure gauge).

Log file sub-VIs:

sub-InitializeLogFile.vi

Should work without modification. Run program, verify that log file appears in indicated folder.

ManualControls.vi

When all of the sub-VIs above have been checked and are working, ManualControls.vi can be set up according to the following steps:

- Set max flow rates of gases at the far left of the block diagram to match MFCs.
- Do the same with the valve and furnace com ports and Baratron pressure max values.
- Adjust the max value of the Desired Pressure numeric control on the front panel. The max should be 10 Torr above room pressure. (right-click, Properties, Data Entry, Maximum)
- Adjust the max values of the MFC flow rates. (right-click, Properties, Data Entry, Maximum)
- Adjust the path to the log folder.
- Set current values to default. (Edit, Make Selected Values Default)
- Save ManualControls.vi

ManualControls.vi is now ready for use.

Use

To use ManualControls.vi, run the VI. If all instruments are powered and properly connected, the charts and numeric indicators will start indicating the CVD's current state.

To change the setpoints of a certain device, use the numeric controls to input your desired setpoint and the buttons to send the setpoint to the device.

Use the “Stop Program” at the end of each session rather than the “Abort Execution” button (in the top tool bar).

Notes:

- Ending the VI does not change any settings – MFC, valve, and furnace setpoints will remain unchanged on stopping the program.
- By closing the valve while gases are flowing, the pressure in the CVD can rise above room pressure. If the CVD does not have a mechanical overpressure relief, CVD pressure could reach unsafe levels.

Read&RunRecipe.vi

Read&RunRecipe.vi can be set up according to the following steps:

- Assign system variables in the top left side of the block diagram.
- Adjust the path to the run log folder on the “Run Recipe” tab of the front panel.
- Set current values to default. (Edit, Make Selected Values Default)

Read&RunRecipe.vi is now ready for use.

Use

To use Read&RunRecipe.vi, run the VI.

You will be prompted for a recipe file. Use Quick.csv.

If the recipe passes the check, this will be indicated in the top right of the “Check Recipe” tab. The simulated run parameters will be shown.

- Increases in pressure are shown as happening instantly, while in reality they will be gradual and depend on the volume of the CVD and the gas flow rates.
- All approximate times are rounded up to the the nearest minute.

To run the recipe, turn on all CVD devices, then click “Run Recipe”.

On the “System Check” tab, the progress of the CVD check can be monitored. This will take about a minute. In the case of system check failure, the CVD will be repressurized with argon and the program will stop.

In the case of system check success, user will be prompted for a log note. If the “Cancel Run” button is pushed, the CVD will be repressureized with argon. If the “OK” button is pushed, the recipe will be run.

To monitor the progress of a recipe, use the “Run Recipe” tab.

When the recipe is finished, the CVD will be repressurized with argon and the VI will stop.

To stop the run before the recipe is finished, press the “End Run” button. The program will then zero all MFC flow rates, set the furnace to 25ºC, repressurize with argon, then stop. The log file will still contain all data gathered before the “End Run” button was pushed.

Both the run log and a copy of the recipe will show up in the log folder.

Arduino Program

```
int MFC1 = 0;   // first analog sensor
int MFC2 = 0;   // second analog sensor
int MFC3 = 0;   // third analog sensor
int MFC4 = 0;   // fourth analog sensor
char inByte;         // incoming serial byte

void setup()
{
  // start serial port at 115200 bps:
  Serial.begin(9600);
}

void loop()
{
  // if we get a valid byte, read analog ins:
  if (Serial.available() > 0) {
    // get incoming byte:
    inByte = Serial.read();
    if (inByte == 'M')
    {
        MFC1 = analogRead(A0); // read analog input
        MFC2 = analogRead(A1); // read analog input
        MFC3 = analogRead(A2); // read analog input
        MFC4 = analogRead(A3); // read analog input
        Serial.print(MFC1);
        Serial.print(",");
        Serial.print(MFC2);
        Serial.print(",");
        Serial.print(MFC3);
        Serial.print(",");
        Serial.println(MFC4);
    }
```

```
  }
}
void establishContact() {
  while (Serial.available() <= 0) {
    Serial.println("0,0,0");   // send an initial string
    delay(300);
  }
}
```

Appendix C: CVD Log Graphing Excel 2013 Macro

```
Sub GraphCVD()
' GraphCVD Macro
' Keyboard Shortcut: Ctrl+g
    ActiveSheet.Shapes.AddChart2(240, xlXYScatterLinesNoMarkers).Select
    ActiveChart.SetSourceData Source:=Range("$C:$C,$D:$E")
    ActiveSheet.Shapes("Chart 1").IncrementLeft -440
    ActiveSheet.Shapes("Chart 1").IncrementTop -100
    ActiveChart.ChartTitle.Text = "Furnace Temperature, Setpoint"
    Selection.Format.TextFrame2.TextRange.Characters.Text = "Furnace Temperature, Setpoint"

    ActiveSheet.Shapes.AddChart2(240, xlXYScatterLinesNoMarkers).Select
    ActiveChart.SetSourceData Source:=Range("$C:$C,$F:$G")
    ActiveSheet.Shapes("Chart 2").IncrementLeft -80
    ActiveSheet.Shapes("Chart 2").IncrementTop -100
    ActiveChart.ChartTitle.Text = "Pressure, Setpoint"
    Selection.Format.TextFrame2.TextRange.Characters.Text = "Pressure, Setpoint"

    ActiveSheet.Shapes.AddChart2(240, xlXYScatterLinesNoMarkers).Select
    ActiveChart.SetSourceData Source:=Range("$C:$C,$H:$H,$J:$J,$L:$L,$N:$N")
    ActiveSheet.Shapes("Chart 3").IncrementLeft 280
    ActiveSheet.Shapes("Chart 3").IncrementTop -100
    ActiveChart.ChartTitle.Text = "Gas Flow Rates"
    Selection.Format.TextFrame2.TextRange.Characters.Text = "Gas Flow Rates"

    ActiveSheet.Shapes.AddChart2(240, xlXYScatterLinesNoMarkers).Select
    ActiveChart.SetSourceData Source:=Range("$C:$C,$I:$I,$K:$K,$M:$M,$O:$O")
    ActiveSheet.Shapes("Chart 4").IncrementLeft 280
    ActiveSheet.Shapes("Chart 4").IncrementTop 116
    ActiveChart.ChartTitle.Text = "Gas Flow Setpoints"
    Selection.Format.TextFrame2.TextRange.Characters.Text = "Gas Flow Setpoints"

    Range("A1").Select
End Sub
```
