# Supplementary material for: Open-source automated chemical vapor deposition system for the production of two- dimensional nanomaterials
Source: PLoS One. 2019 Jan 16;14(1):e0210817. doi: 10.1371/journal.pone.0210817 (PMC6334948; doi:10.1371/journal.pone.0210817)
Supplement: S3 Appendix — (PDF) [file pone.0210817.s003.pdf]

Open-source automated chemical vapor deposition system for the production of 2- dimensional materials

S3 Appendix: **Growth and CVD system details.**

1. Graphene growth by CVD
2. Graphene transfer process
3. WS_2 growth by CVD
4. Representative temperature ramp profile obtained from system during growth
5. CVD system temperature profile

1. Graphene growth by CVD

Copper foil (Alfa Aesar, 0.5mm thick, 99.99%) was cut into a 3.63 cm^2 square sample and placed into CVD chamber. The graphene growth was completed using the recipe in Table 1. After execution of the growth recipe, the sample was removed from the CVD system and coated with 495 PMMA A2 (MicroChem). The sample is coated by dropping PMMA on sample with a pipet, and then turning sample to the side to allow excess PMMA to drip off. The coated sample was then baked on a hot plate at 200 °C for 2 minutes. Next, the sample was similarly coated with 950 PMMA A4 (MicroChem) and also baked at 200 °C for 2 minutes. Samples were then stored in a desiccator until next step could be completed.

Table 1. Graphene growth recipe

**Stage**	**0**	**1**	**2**	**3**	**4**	**5**
Pressure setpoint (Torr)	1	1	1	1	1	690
Temperature Setpoint (°C)	25	1000	1000	1000	200	25
Temperature Ramp Rate (°C/min, 30°C/min max)	0					
Ar flow (sccm)	0	100	100	0	100	500
CH_4 flow (sccm)	0	0	0	850	0	0
H_2 flow (sccm)	0	0	100	50	0	0
C_2H_4 flow (sccm)	0	0	0	0	0	0
Dwell time (min)	0		90	90	0	0
Stage End Condition	Start	Temp	Time	Time	Temp	End

2. Graphene transfer process

The graphene was removed from the copper foil via an electrochemical delamination process. The delamination process is detailed in Table 2. In this experiment, the graphene film was transferred to single polished sapphire (C plane) substrates. The graphene on sapphire was baked overnight at 80°C. PMMA was then removed via an acetone vapor bath, on a hotplate at 45°C for ~24 hours. The samples were then placed into a 50ml tube with acetone which was placed into a water bath held at 80- 100°C for 3 hours, or until PMMA is dissolved.

Table 2. Electrochemical delamination process

Step	Description	Explanations
1	Setting up the electrolyte and securing the electrodes<br>• Fill a beaker with 0.5 M NaCl solution<br>• Secure the platinum mesh[1] and the Ag/AgCl electrode[2] to the side of the beaker using the parafilm	[1]The Platinum mesh is the Counter electrode<br>[2]The Ag/AgCl is the Reference electrode
2	Connecting the Potentiostat[3] to the electrodes<br>• The wires coming out from the potentiostat were labeled. The reference wire was connected to the Ag/AgCl electrode and the counter electrode wire was connected to the platinum mesh.	[3]A Biologic VSP-300 Potentiostat
3	Connecting the Potentiostat to the copper/graphene/PMMA substrate[4]<br>• The substrate was connected to the third wire (Working electrode) coming out of the potentiostat	[4]The copper/graphene/PMMA substrate is the working electrode
4	Beginning the electrochemical delamination process<br>• The working electrode substrate was then submerged in the electrolyte (only submerged about 2-4 mm) and held at a 45° angle[5]<br>• Once submerged, the BioLogic[6] was set to -2.1V<br>• Once the potentiostat is working, there should be bubbles coming from the sample	[5]In order to begin, the substrate needs to be submerged in the electrolyte; it completes the circuit.<br>[6] The BioLogic is what is used to operate the potentiostat
5	Film separating from the copper<br>• As the film starts to separate, submerge the substrate deeper into the electrolyte<br>• A toothpick[7] was used to loosen the film from the edge of the substrate<br>• This step took 30-40 minutes	[7]The toothpick was small enough and non-conductive making it a useful tool in this process
6	Film has separated from the copper	[8]Nanopure water was used throughout this process

	• Once the film has been separated, shut the BioLogic off<br>• Gently remove the film using a glass slide<br>• Place the film in a beaker of water[8] that is on a hotplate that has been heated to 80 °C[9]<br>• After being left on the water for 5-10 mins, transfer to another beaker of water and let sit for 5 mins. Then transfer the film again to another beaker of water.	[9]This is done to rinse the NaCl solution off the film
7	Transfer the film to Sapphire<br>• Once in the last beaker of water, gently transfer the film to the sapphire by submerging the sapphire in the water and placing it underneath the film<br>• Gently lift the sapphire up out of the water at a 45° angle[10]<br>• Let the Sapphire and film sit in the oven at 80 °C overnight[11]	[10]Be gentle. The film is delicate and can tear. Lifting the film up at a 45° angle also helps so that there is no water trapped between the sapphire and the film<br>[11]To help the film adhere to the sapphire better

3. WS_2 growth by CVD

The atmospheric CVD growth for WS_2 took place at 850 °C, with argon flowing at 70 sccm and hydrogen flowing at 15 sccm for 15 minutes. The growth recipe for WS_2 is given in Table 3. WS_2 was deposited on graphene/ sapphire substrates.

Table 3. WS_2 growth recipe

**Stage**	**0**	**1**	**2**	**3**	**4**	**5**	**6**
Pressure setpoint (Torr)	690	690	690	690	690	690	690
Temperature setpoint (°C)	25	250	625	850	850	300	25
Temperature Ramp Rate (°C/min, 30°C/min max)			20	10			
Ar flow (sccm)	0	500	70	70	70	70	70
CH_4 flow (sccm)	0	0	0	0	0	0	0
H_2 flow (sccm)	0	0	0	0	15	0	0
C_2H_4 flow (sccm)	0	0	0	0	0	0	0
Dwell time (min)	0	5	0	0	15	0	0
Stage End Condition	Start	Time	Temp	Temp	Time	Temp	End

4. Representative temperature profile obtained from system during growth

The following temperature profile is given as an example of a possible heat up and cool down cycle. The temperature ramp rate may be adjusted with the automated or manual controls.

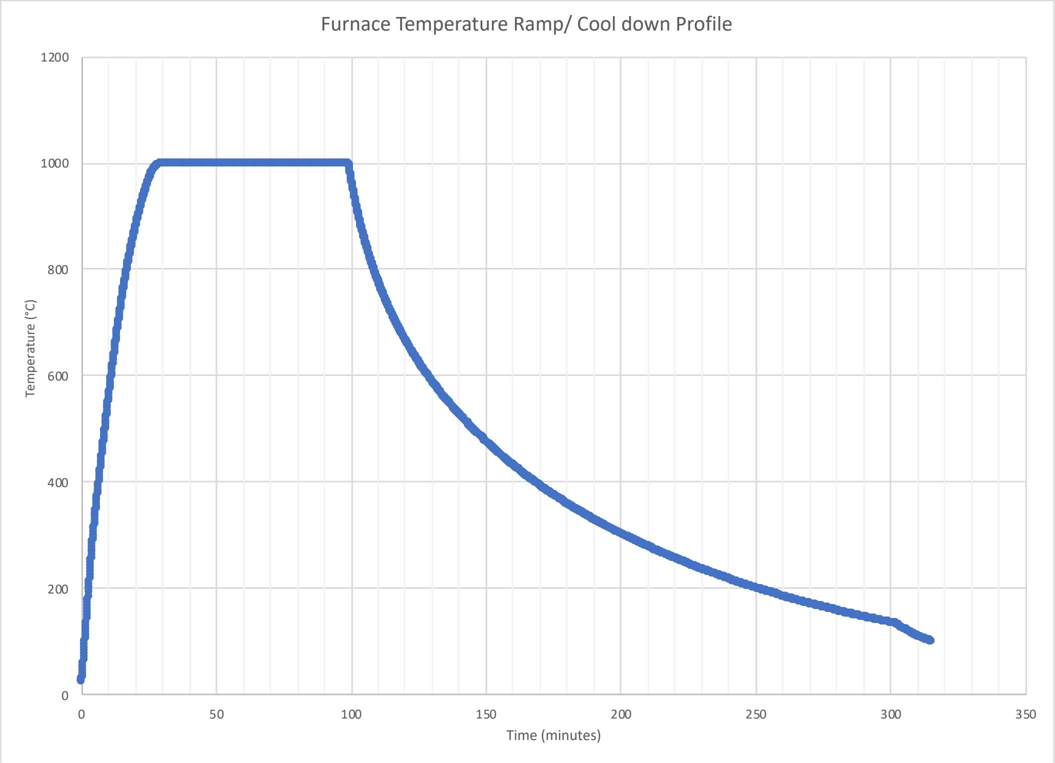

Figure 1. Profile of temperature ramp rate/ cool down obtained from CVD system.

4. CVD system temperature profile

Figure 2 shows the temperature profile for the reaction chamber, measured at a set point of 850 degrees Celsius. The distance is measured from the center to the edge of the chamber walls.

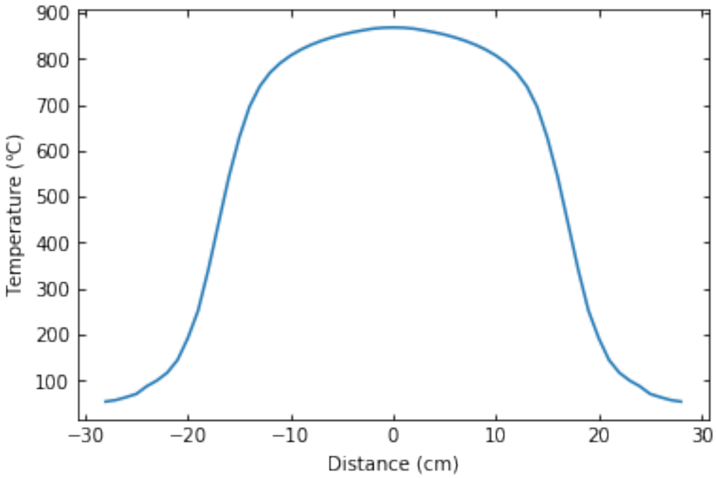

Figure 2. Temperature profile of reaction chamber.
