# Supplementary material for: Open-source automated chemical vapor deposition system for the production of two- dimensional nanomaterials
Source: PLoS One. 2019 Jan 16;14(1):e0210817. doi: 10.1371/journal.pone.0210817 (PMC6334948; doi:10.1371/journal.pone.0210817)
Supplement: S2 Folder — Folder contains construction drawings. (ZIP) [file pone.0210817.s005.zip › Support Drawings/Large Support Base.PDF]

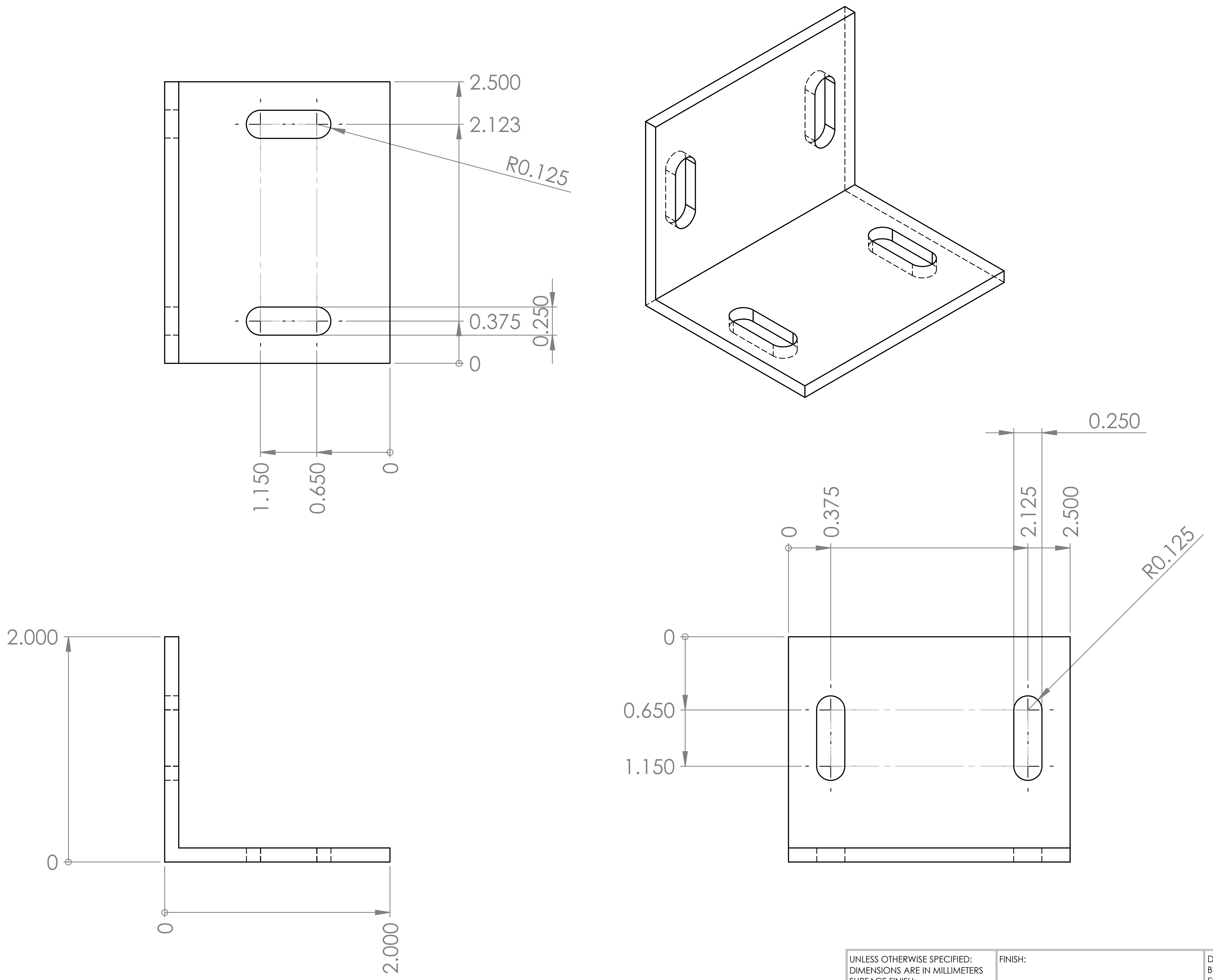

Tolerances X.XX +/- 0.015
X.XXX+/- 0.005

Quantity: 2

UNLESS OTHERWISE SPECIFIED:<br>DIMENSIONS ARE IN MILLIMETERS<br>SURFACE FINISH:<br>TOLERANCES:<br>LINEAR:<br>ANGULAR:	FINISH:				DEBUR AND<br>BREAK SHARP<br>EDGES	DO NOT SCALE DRAWING	REVISION
	NAME	SIGNATURE	DATE			TITLE:	
DRAWN							
CHK'D							
APPV'D							
MFG							
Q.A				MATERIAL:		DWG NO. Large Angle	A3
				WEIGHT:		SCALE:1:1	SHEET 1 OF 1
