# Supplementary material for: Open-source automated chemical vapor deposition system for the production of two- dimensional nanomaterials
Source: PLoS One. 2019 Jan 16;14(1):e0210817. doi: 10.1371/journal.pone.0210817 (PMC6334948; doi:10.1371/journal.pone.0210817)
Supplement: S2 Folder — Folder contains construction drawings. (ZIP) [file pone.0210817.s005.zip › Support Drawings/MFC Support.PDF]

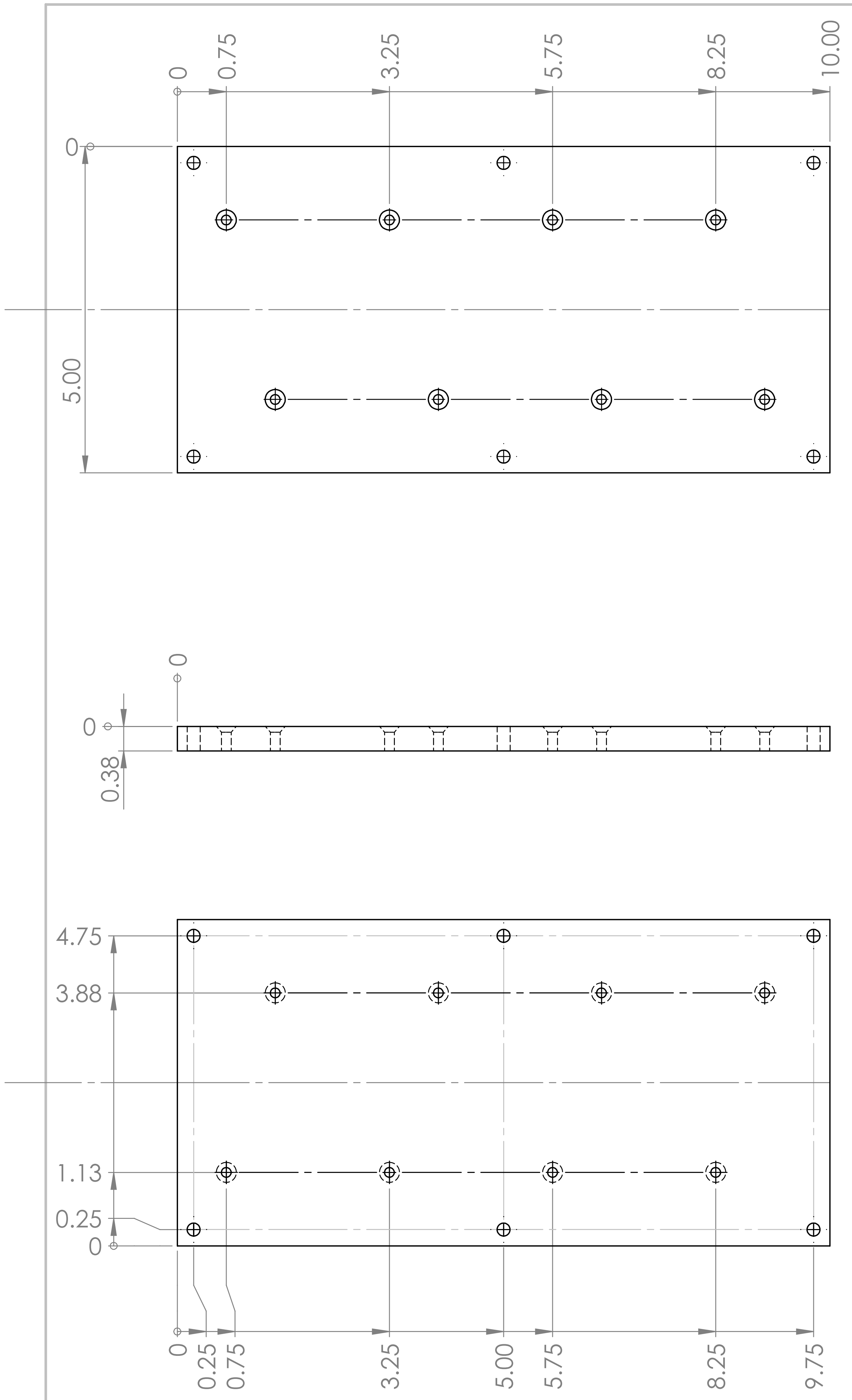

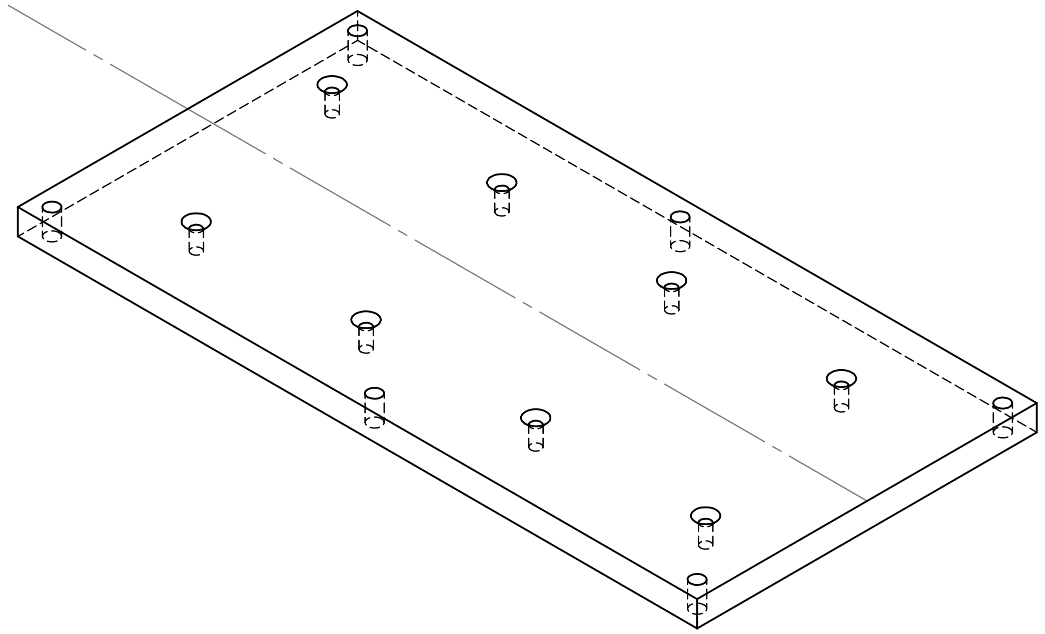

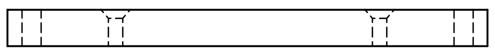

Bores Are 8-32
Tolerances X.XX +/- 0.015
X.XXX+/- 0.005

UNLESS OTHERWISE SPECIFIED: DIMENSIONS ARE IN MILLIMETERS SURFACE FINISH: TOLERANCES: LINEAR: ANGULAR:		FINISH:		DEBUR AND BREAK SHARP EDGES	DO NOT SCALE DRAWING	REVISION	
	NAME	SIGNATURE	DATE		TITLE:		
DRAWN							
CHK'D							
APPV'D							
MFG							
Q.A				MATERIAL:	DWG NO.	MAF Holder	A3
				WEIGHT:	SCALE:1:2	SHEET 1 OF 1	
