# Supplementary material for: Open-source automated chemical vapor deposition system for the production of two- dimensional nanomaterials
Source: PLoS One. 2019 Jan 16;14(1):e0210817. doi: 10.1371/journal.pone.0210817 (PMC6334948; doi:10.1371/journal.pone.0210817)
Supplement: S2 Folder — Folder contains construction drawings. (ZIP) [file pone.0210817.s005.zip › Support Drawings/Exhaust Line Support.PDF]

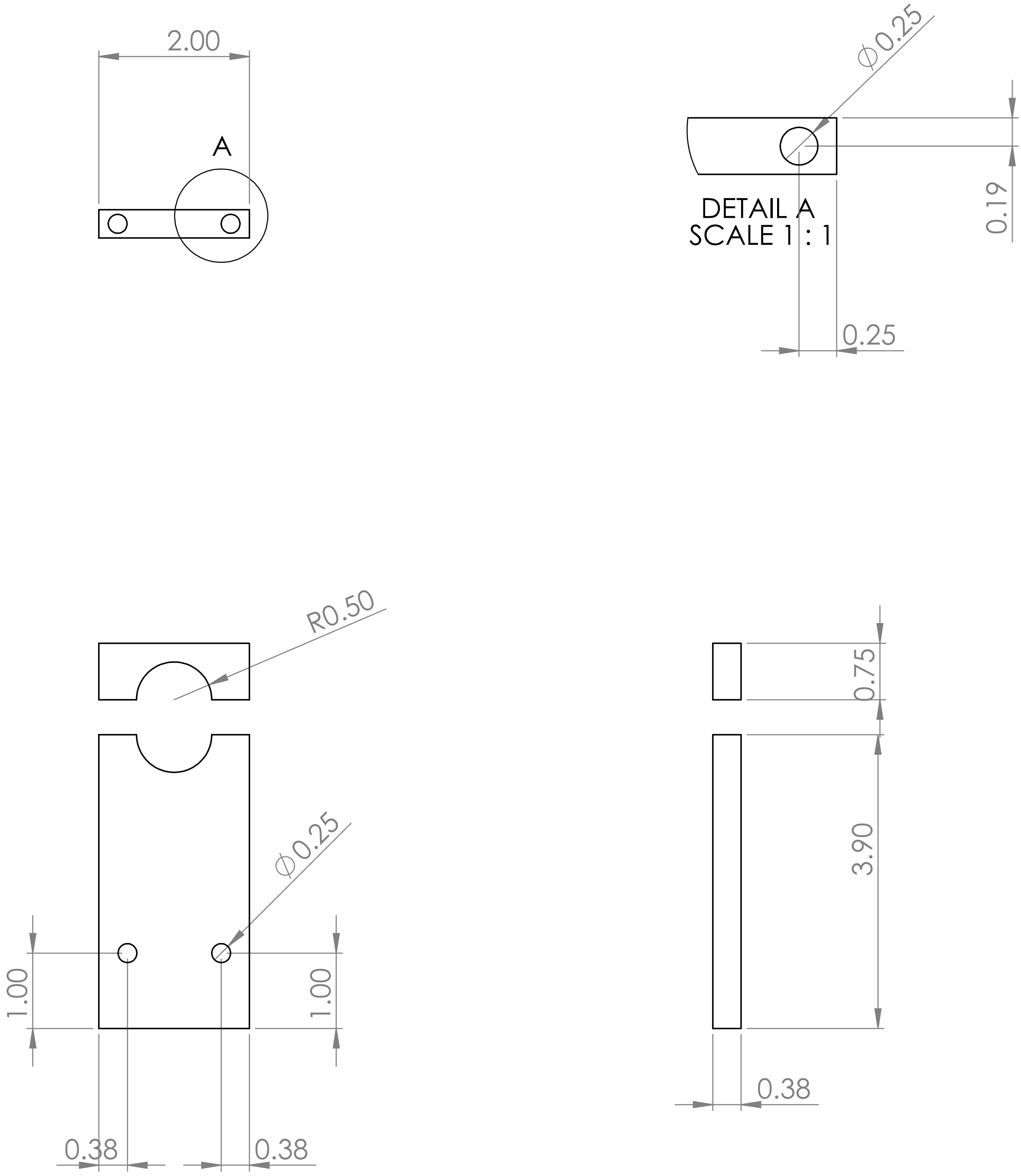

UNLESS OTHERWISE SPECIFIED:
DIMENSIONS ARE IN MILLIMETERS
SURFACE FINISH:
TOLERANCES:
LINEAR:
ANGULAR:

FINISH:

DEBUR AND BREAK SHARP EDGES

DO NOT SCALE DRAWING

REVISION

	NAME	SIGNATURE	DATE
DRAWN			
CHK'D			
APPV'D			
MFG			
Q.A			

TITLE:

MATERIAL:

WEIGHT:

DWG NO. Intake-25=short

A4

SCALE:1:2

SHEET 1 OF 1
